# Polyketides from the Mangrove-Derived Endophytic Fungus *Cladosporium cladosporioides*

**DOI:** 10.3390/md17050296

**Published:** 2019-05-17

**Authors:** Fan-Zhong Zhang, Xiao-Ming Li, Xin Li, Sui-Qun Yang, Ling-Hong Meng, Bin-Gui Wang

**Affiliations:** 1 Key Laboratory of Experimental Marine Biology, Center for Ocean Mega-Science, Institute of Oceanology, Chinese Academy of Sciences, Nanhai Road 7, Qingdao 266071, China; fancyzfz@163.com (F.-Z.Z.); lixmqdio@126.com (X.-M.L.); lixin871014@163.com (X.L.); suiqunyang@163.com (S.-Q.Y.); 2 Laboratory of Marine Biology and Biotechnology, Qingdao National Laboratory for Marine Science and Technology, Wenhai Road 1, Qingdao 266237, China; 3 University of Chinese Academy of Sciences, Yuquan Road 19A, Beijing 100049, China

**Keywords:** mangrove plant, endophytic fungus, *Cladosporium cladosporioides*, polyketides, antimicrobial activity, acetylcholinesterase, enzymatic inhibitory activity

## Abstract

Five new polyketides, namely, 5*R*-hydroxyrecifeiolide (**1**), 5*S*-hydroxyrecifeiolide (**2**), *ent*-cladospolide F (**3**), cladospolide G (**4**), and cladospolide H (**5**), along with two known compounds (**6** and **7**), were isolated from the endophytic fungal strain *Cladosporium cladosporioides* MA-299 that was obtained from the leaves of the mangrove plant *Bruguiera gymnorrhiza.* The structures of these compounds were established by extensive analysis of 1D/2D NMR data, mass spectrometric data, ECDs and optical rotations, and modified Mosher’s method. The structures of **3** and **6** were confirmed by single-crystal X-ray diffraction analysis and this is the first time for reporting the crystal structures of these two compounds. All of the isolated compounds were examined for antimicrobial activities against human and aquatic bacteria and plant pathogenic fungi as well as enzymatic inhibitory activities against acetylcholinesterase. Compounds **1**–**4**, **6**, and **7** exhibited antimicrobial activity against some of the tested strains with MIC values ranging from 1.0 to 64 μg/mL, while **3** exhibited enzymatic inhibitory activity against acetylcholinesterase with the IC_50_ value of 40.26 μM.

## 1. Introduction

**Figure 1:**
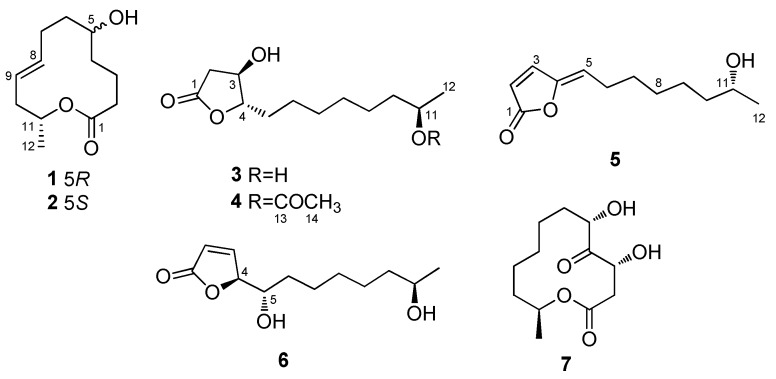
Structures of the isolated compounds **1**–**7**.

The *Cladosporium* fungi, one of the largest genera of dematiaceous hyphomycetes, have attracted considerable attention of natural products researchers in recent years [1,2]. Versatile bioactive metabolites, such as cladosporin [3], macrolide [4], sulfur-containing diketopiperazines [5], indole alkaloids [6], hybrid polyketides [7], and diterpenes with 5-8-5 ring system [8], have been isolated from the *Cladosporium* strains. As part of our research on discovering structurally novel and biologically active natural products, a series of interesting metabolites have been obtained from marine-derived fungal strains [9,10], including those from *Cladosporium* species [11]. Our current chemical investigation on *C. cladosporioides* MA-299, an endophytic fungus obtained from the fresh inner leaves of the marine mangrove plant *Bruguiera gymnorrhiza*, led to the discovery of five new polyketides, namely, 5*R*-hydroxyrecifeiolide (**1**), 5*S*-hydroxyrecifeiolide (**2**), *ent*-cladospolide F (**3**) [12], cladospolide G (**4**), and cladospolide H (**5**) (Figure 1), as well as two known analogues, including *iso*-cladospolide B (**6**) [13,14], and pandangolide 1 (**7**) [13,15] (Figure 1). Herein, we report the isolation, structure assignment, and biological evaluation of the isolated compounds.

## 2. Results and Discussion

### 2.1. Structure Elucidation of the New Compounds

**Figure 2:**
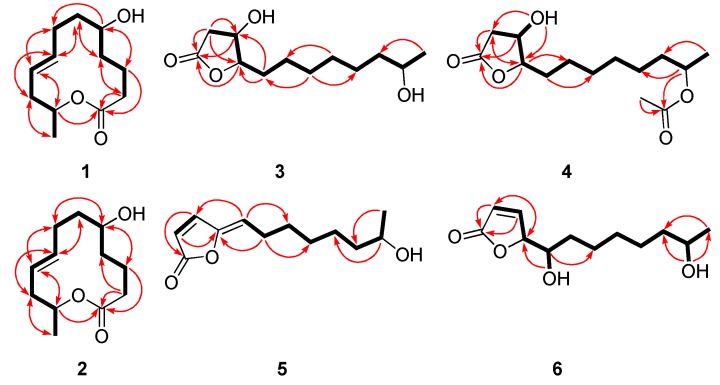
Key COSY (bold lines) and HMBC (red arrows) correlations for **1**–**6**.

**Figure 3:**
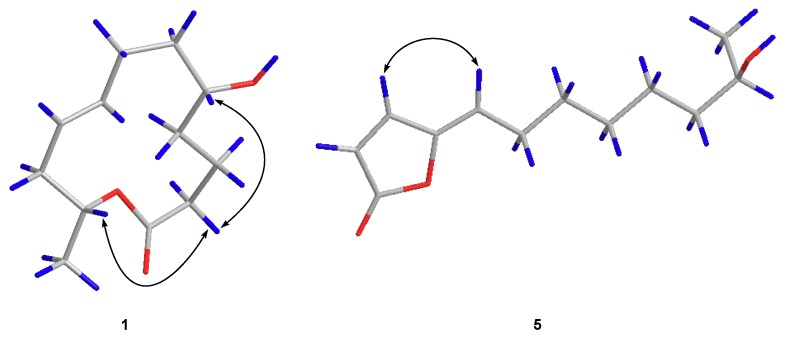
Key NOESY correlations for **1** and **5**.

**Figure 4:**
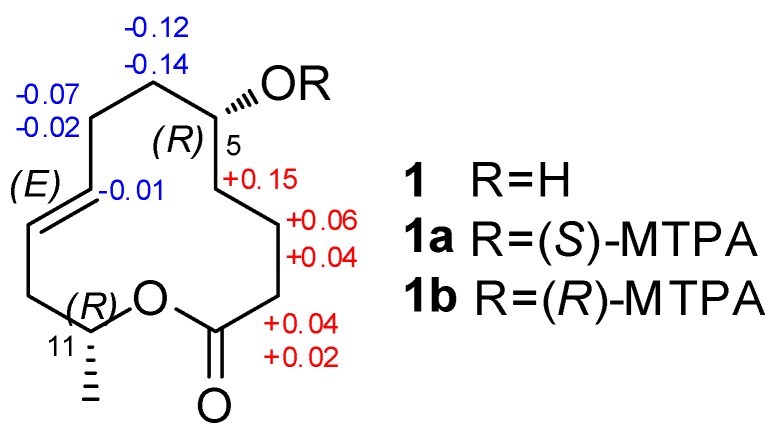
∆*δ* values (∆*δ* (in ppm) = *δ_S_* − *δ_R_*) obtained for the (*S*)-and (*R*)-MTPA esters (**1a** and **1b**, respectively) of **1**.

**Figure 5:**
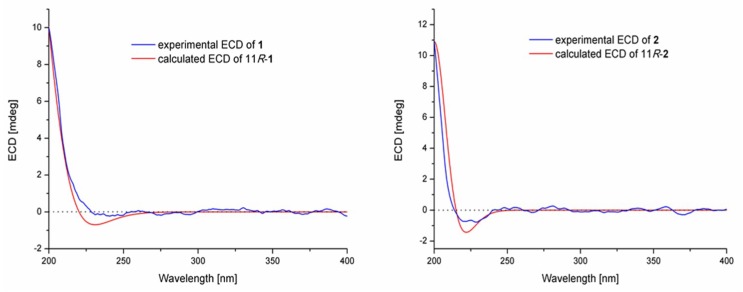
Comparison of experimental and calculated ECD spectra of **1** and **2**.

5*R*-Hydroxyrecifeiolide (**1**) was isolated as a colorless oil and the molecular formula C_12_H_20_O_3_ was deduced from the (+)-HRESIMS data, indicating three degrees of unsaturation. The ^1^H and ^13^C NMR spectra of **1** showed the signals for one ester/lactone carbonyl, two olefinic and two oxygenated sp^3^ methines, six sp^3^ methylenes, and one methyl group (Table 1). In addition, the ^1^H NMR data of **1** were quite similar to those of recifeiolide (11-hydroxy-*trans*-8-dodecenoic acid lactone) [16,17], except that one methylene (*δ*_H_ 1.5–2.3 ppm) in recifeiolide was replaced by an oxygenated methine (*δ*_H_ 3.51 ppm) in **1**. The key COSY correlations elucidated the connectivity from H-2 through H-12 (Figure 2). Key HMBC correlations from H-2 to C-1 and C-4, from H-3 to C-1 and C-5, and from H-11 to C-1, connected C-1 and C-2 and determined the 12-membered macrolide skeleton of **1** (Figure 2). The relative configuration at C-5 and C-11 for **1** was established by the NOESY experiment (Figure S8). The NOESY correlations (Figure 3) from H-2*β* to H-5 and H-11 revealed a *β* orientation of these protons [18]. The coupling constants between H-8 and H-9 (*J*_H-8/H-9_ = 15.3 Hz) suggested the *E*-configuration of the C-8/C-9 double bond. The absolute configuration of C-5 of **1** was assigned by application of the modified Mosher’s method [19]. The ∆*δ* values obtained for the (*S*)- and (*R*)-MTPA esters (**1a** and **1b**, respectively) of **1** (Figure 4) suggested that the absolute configuration of C-5 is *R*. Furthermore, the electronic circular dichroism (ECD) spectrum of **1** was recorded and then computed with the time-dependent density function theory (TD-DFT) method at the gas-phase B3LYP/6-31G (d) level [20,21]. The calculated ECD spectra were produced by SpecDis software [22]. The experimental ECD spectrum for **1** matched well with the calculated spectrum for 11*R* (Figure 5). Therefore, the 5*R*, 11*R* configuration of **1** was established, and the trivial name 5*R*-hydroxyrecifeiolide was assigned.

The molecular formula of **2** was determined as C_12_H_20_O_3_, which was the same as that of **1,** according to its (+)-HRESIMS data. The ^1^H and ^13^C NMR spectra (Table 1) of **2** were similar to those of **1**, except for the different ^13^C chemical shifts at C-5 (*δ*_C_ 70.2 in **1**, and *δ*_C_ 65.4 in **2**) and its adjacent positions (C-2–C-4 and C-6–C-8), which indicated that compound **2** was the 5-epimer of **1**. The chemical shifts at C-2 and C-8 (*γ*-position of C-5) exhibited obvious difference in **2** and **1** probably due to the space effect. As expected, the experimental ECD spectrum of **2** matched well with the calculated spectrum of 11*R* (Figure 5). The trivial name 5*S*-hydroxyrecifeiolide was assigned to **2**.

**Figure 6:**
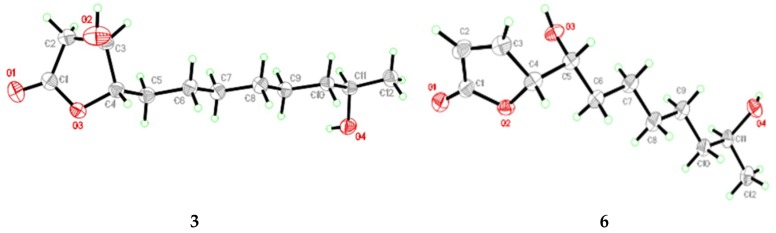
Ortep diagrams of *ent*-cladospolide F (**3**) and *iso*-cladospolide B (**6**).

Compound **3** was initially obtained as pale yellow powder and possessed a molecular formula C_12_H_22_O_4_ by (+)-HRESIMS, implying two degrees of unsaturation. The ^1^H and ^13^C NMR data (Table 2) exhibited signals attributed to one ester carbonyl, three oxygenated sp^3^ methines, seven sp^3^ methylenes, and one methyl group. These data were very similar to those of cladospolide F [12], suggesting that they had the same planar structure, which was also confirmed by the COSY and HMBC correlations (Figure 2). However, the signs of the optical rotations of **3** (−29.41, MeOH) and cladospolide F (+15.7, MeOH) were opposite, indicating that the absolute configurations of their stereogenic carbons were different. The relative configuration at C-3, C-4, and C-11 could not be concluded by NOESY experiment. Nevertheless, suitable crystals were obtained for X-ray diffraction analysis using Cu Kα radiation which confirmed the absolute configuration of C-3, C-4 and C-11 as 3*R*, 4*S*, and 11*R* (Figure 6). The *ent*-cladospolide F was therefore assigned as a trivial name for **3**.

Compound **4** was obtained as a pale yellow oil and its molecular formula was determined as C_14_H_24_O_5_ on the basis of (+)-HRESIMS, requiring three degrees of unsaturation. The ^1^H and ^13^C NMR data for **4** (Table 2) were quite similar to those of **3,** except for the presence of additional ester carbonyl (C-13) and methyl (C-14) groups, which indicated the replacement of 11-OH group in **3** by an OAc group in **4**, and thus caused the down-field shift of 11-H from *δ*_H_ 3.55 in **3** to *δ*_H_ 4.78 in **4**. Detailed interpretation of the COSY and HMBC spectra revealed that **4** was an analogue of **3**, with the hydroxyl group at C-11 in **3** being replaced by an acetoxyl group in **4**. The HMBC correlation from H-11 to C-13 established the presence of an acetoxyl group at C-11, and the planar structure of **4** was hence confirmed as shown (Figure 2). In a biogenetic perspective, it was tentatively assigned the same relative configuration as that of **3**. The similar optical rotations of **4** (−24.56, MeOH) and **3** (−29.41, MeOH) also supported that the absolute configurations of the stereogenic carbons in **4** were the same as those in **3**. Therefore, the absolute configurations of the stereogenic carbons in **4** were tentatively assigned as 3*R*, 4*S*, and 11*R*, and the trivial name cladospolide G was assigned. Acetylation of compounds **3** and **4** using acetyl chloride yielded the same diacetylated derivative, which further correlated the structure relationship of compounds **3** and **4**.

Compound **6** was isolated as colorless crystals and gave ion peaks at *m/z* 229.1432 [M + H]^+^ and 246.1699 [M + NH_4_]^+^ in the (+)-HRESIMS, corresponding to a molecular formula C_12_H_20_O_4_, indicating three degrees of unsaturation. All the ^1^H and ^13^C NMR data of **6** were quite similar to those of the previously reported polyketide metabolite *iso*-cladospolide B [13]. The COSY and HMBC correlations (Figure 2) confirmed that the planar structure of **6** was the same as that of *iso*-cladospolide B. The high similarity of specific rotations of **6** (${\left[\alpha \right]}_{D}^{25}$ = −90.91 (*c* 0.11, MeOH) ) and *iso*-cladospolide B (${\left[\alpha \right]}_{D}^{25}$= −90 (*c* 0.23, MeOH)) [13] suggested that they may have the same relative and absolute stereochemistry. However, neither the relative nor the absolute configuration was determined [13]. In 2001, Franck et al. carried out the first synthesis of *iso*-cladospolide B and proposed that it has the 4*S*, 5*S*, and 11*R* configuration [14]. Later, in 2005, the absolute configuration of *iso*-cladospolide B, isolated from *Cladosporium sp*. isolated from the Red Sea sponge *Niphates rowi*, was assigned to be 4*S*, 5*S*, and 11*S* (${\left[\alpha \right]}_{D}^{28}$ = −61 (*c* 16.6, MeOH)) [15]. It was later stated that both diastereomers appear to be natural products and (4*S*, 5*S*, 11*S*)-isomer referred to as 11-*epi*-*iso*-cladospolide B [23,24]. The relative configuration of **6** could not be assigned by NOESY experiments but the coupling constant for C-4 (*J* = 1.5 Hz) confirmed the *threo* relative configuration [14]. Upon slow evaporation of the solvent (MeOH-H_2_O), compound **6** was crystallized and the X-ray analysis was carried out, which was first reported for *iso*-cladospolide B (Figure 4). The Cu Kα Flack parameter 0.5 (7) allowed preliminary confirmation of the relative configurations of **6** as 4S*, 5S*, 11R*.

Compound **5** was obtained as a pale yellow oil and possessed a molecular formula of C_12_H_18_O_3_ by (+)-HRESIMS, implying four degrees of unsaturation. The 1D NMR data (Table 2) and HSQC spectrum (Figure S38) suggested signals attributed to one ester and one olefinic quaternary carbons, one oxygenated and three olefinic methines, five sp^3^ methylenes, and one methyl group. These NMR data were similar to those of *iso*-cladospolide B (**6**) [13]. However, resonances for two oxygenated methines (C-4 and C-5) in **6** were not detected in the NMR spectra of **5**. Instead, two additional olefinic signals including one quaternary sp^2^ (C-4, *δ*_C_ 149.4) and one methine sp^2^ (C-5, *δ*_C_ 117.1/*δ*_H_ 5.53) carbons were observed in the NMR spectra of **5** (Table 2). These data indicated that **5** was a reduced analogue of **6**, and this deduction was supported by the molecular formula. The COSY and HMBC spectra established the structure of **5** as shown in Figure 1. In the NOESY experiment, the correlation between H-3 and H-5 indicated the *Z*-conformation of the double bond between C-4 and C-5 (Figure 3). The absolute stereochemistry of **5** could not be determined by Mosher’s method because of the limited amount of material available. From a biogenetic point of view, **5** was putatively produced by reduction of **6**. Therefore, it was tentatively assigned the absolute configuration of C-11 of **5** as 11*R*. From these data, the name cladospolide H was assigned for **5**.

Compound **7** was acquired as white powder and showed ion peaks at *m/z* 267.1197 [M + Na]^+^ in the positive HRESIMS, corresponding to a molecular formula of C_12_H_20_O_5_. A literature search indicated that all the ^1^H and ^13^C NMR data of **7** were almost the same as those of previously reported compound pandangolide 1 [13,15]. The almost exactly the same specific rotations of **7** (${\left[\alpha \right]}_{D}^{25}$ = −30.16 (c 1.22, MeOH)) and pandangolide 1 (${\left[\alpha \right]}_{D}^{25}$= −30 (c 2.3, MeOH)) [15] revealed that they may have the same relative and absolute configurations.

### 2.2. Biological Activities of the Isolated Compounds

Compounds **1**–**7** were tested for antimicrobial activities against two human pathogens (*Escherichia coli*, *Staphylococcus aureus*), ten aquatic bacteria (*Aeromonas hydrophilia*, *Edwardsiella ictarda*, *E. tarda*, *Micrococcus luteus*, *Pseudomonas aeruginosa*, *Vibrio alginolyticus*, *V. anguillarum*, *V. harveyi*, *V. parahaemolyticus*, and *V. vulnificus*), and 15 plant pathogenic fungi (*Alternaria solani*, *Bipolaris sorokiniana*, *Ceratobasidium cornigerum*, *Colletotrichum glecosporioides*, *Coniothyrium diplodiella*, *Fusarium graminearum*, *F. oxysporum* f. sp. *cucumerinum*, *F. oxysporum* f. sp. *momodicae*, *F. oxysporum* f. sp. *radicis lycopersici*, *F. solani*, *Glomerella cingulate*, *Helminthosporium maydis*, *Penicillium digitatum*, *Physalospora piricola Nose*, and *Valsa mali*). As shown in Table 3, **3** exhibited moderate inhibitory activities against human pathogenic bacteria S. aureus with MIC value of 8.0 μg/mL. Compound **4** showed potent inhibitory activities against plant-pathogenic fungi (*G. cingulate* and *F. oxysporum* f. sp. *cucumerinum*), each with an MIC value of 1.0 μg/mL, while **7** showed activity against aquatic bacterium (E. ictarda) and plant-pathogenic fungus (*G. cingulate*), with MIC values of 4.0 and 1.0 μg/mL, respectively.

Compounds **1**–**7** were also evaluated for acetylcholinesterase inhibitory activity. Compound **3** exhibited potent activity against acetylcholinesterase with the IC_50_ value of 40.26 *μ*M. The other compounds have a weak activity (IC_50_ > 50 μM).

## 3. Experimental Section

### 3.1. General Experimental Procedures

Melting points were determined by an SGW X-4 micro-melting-point apparatus (Shanghai Shenguang Instrument Co. Ltd, Shanghai, China). Optical rotations were measured on an Optical Activity AA-55 polarimeter (Optical Activity Ltd., Cambridgeshire, UK). UV spectra were measured on a PuXi TU-1810 UV-visible spectrophotometer (Shanghai Lengguang Technology Co. Ltd., Shanghai, China). ECD spectra were acquired on a Chirascan spectropolarimeter (Applied Photophysics Ltd., Leatherhead, UK). The ^1^H, ^13^C, and 2D NMR spectra were acquired using a Bruker Avance 500 or 600 M spectrometer (Bruker Biospin Group, Karlsruhe, Germany). Chemical shifts (*δ*) were expressed in ppm with reference to the solvent peaks (^13^C, CDCl_3_: 77.16 ppm, DMSO-*d*_6_: 39.52 ppm; ^1^H, CDCl_3_: 7.26 ppm, DMSO-*d*_6_: 2.50 ppm). Mass spectra were obtained from an API QSTAR Pulsar 1 mass spectrometer (Applied Biosystems, Foster, Waltham, MA, USA). Analytical HPLC analyses were performed using a Dionex HPLC system (Dionex, Sunnyvale, CA, USA) equipped with P680 pump, ASI-100 automated sample injector, and UVD340U multiple wavelength detector controlled by Chromeleon software (version 6.80). Column chromatography (CC) was performed with silica gel (200–300 mesh, Qingdao Haiyang Chemical Factory, Qingdao, China), Lobar LiChroprep RP-18 (40–60 *μ*m, Merck, Darmstadt, Germany), and Sephadex LH-20 (18–110 μm, Merck).

### 3.2. Fungal Material

The fungal strain *Cladosporium cladosporioides* MA-299 was isolated from the leaves of the mangrove plant *Bruguiera gymnorrhiza*, collected in Hainan Island, China, in March 2015. The strain was identified as *Cladosporium cladosporioides* by analysis of its ITS region of the rDNA, which is the same (100%) as that of *C. cladosporioides* DCF-1 (accession no. MG208055). The sequence data were deposited in GenBank with the accession number MH822624. The strain is preserved at Key Laboratory of Experimental Marine Biology, Institute of Oceanology of the Chinese Academy of Sciences (IOCAS).

### 3.3. Fermentation

For chemical investigations, the strain of *C. cladosporioides* MA-299 was cultured on PDA (Potato Dextrose Agar) medium at 28 °C for six days and then inoculated into 100 × 1 L flasks, each containing 70 g of rice, 0.1 g corn syrup, 0.3 g peptone, 0.1 g methionine and 100 mL seawater that was obtained from the Huiquan Gulf of the Yellow Sea near the campus of IOCAS, statically cultured for 48 days at room temperature.

### 3.4. Extraction and Isolation

After 48 days, the fermented rice substrate was mechanically fragmented and then extracted three times with 300 mL EtOAc every flask. All of the EtOAc extracts were filtered and evaporated under reduced pressure to yield a crude extract (52.3 g).

The crude extract was subjected to a silica gel vacuum liquid chromatography (VLC), eluting with different solvents of increasing polarity from petroleum ether (PE) to MeOH to yield ten fractions (Frs. 1–10) based on TLC and HPLC analysis. Fr. 5 (2.1 g) was further purified by reversed-phase column chromatography (CC) over Lobar LiChroprep RP-18 with a MeOH-H_2_O gradient (from 10: 90 to 100: 0) to afford four subfractions (Frs. 5.1–5.4). Fr. 5.2 was further purified by CC on Sephadex LH–20 (MeOH) and then by preparative TLC (plate: 20 × 20 cm, developing solvents: CH_2_Cl_2_/MeOH, 30: 1) to obtain **5** (2.6 mg). Fr. 5.3 was subjected to CC on silica gel eluted with CH_2_Cl_2_-MeOH (100:1 to 5:1) to obtain **1** (4.1 mg) and **2** (3.0 mg). Fr. 6 (1.7 g) was further fractionated by CC over Lobar LiChroprep RP-18 with a MeOH/H_2_O gradient (from 10:90 to 100:0) to yield six subfractions (Frs. 6.1–6.6). Fr. 6.1 (112.4 mg) was further purified by prep. TLC (plate: 20 × 20 cm, developing solvents: petroleum ether/acetone, 2:1) and then on Sephadex LH–20 (MeOH) to obtain **7** (3.2 mg). Fr. 6.5 was subjected to CC on silica gel eluted with CH_2_Cl_2_-MeOH (150:1 to 70:1) to obtain **4** (5.7 mg). Further purification of Fr. 7 (3.6 g) by CC over Lobar LiChroprep RP-18 with a MeOH/H_2_O gradient (from 10:90 to 100:0) yielded seven subfractions (Frs. 7.1–7.7). Fr. 7.1 (736.2 mg) was purified by CC on silica gel eluting with a petroleum ether-acetone gradient (from 10:1 to 2:1), and further fractionated by Sephadex LH-20 (MeOH) to afford **3** (91.6 mg). Fr. 7.2 (960.7 mg) was further separated by CC on silica gel eluting with a petroleum ether-acetone gradient (from 10:1 to 1:1) purification, to afford **6** (23.4 mg).

5*R*-Hydroxyrecifeiolide (**1**): Colourless oil; ${\left[\alpha \right]}_{D}^{25}$ +33.33 (*c* 0.09, MeOH); UV (MeOH) *λ*_max_ (log *ε*) 205 (3.06), 220 (3.01); ECD (7.55 mM, MeOH) *λ*_max_ (Δ*ε*) 233 (−0.01) nm; ^1^H and ^13^C NMR data, see Table 1; ESIMS *m/z* 235 [M + Na]^+^; (+)-HRESIMS at *m/z* 235.1298 [M + Na]^+^ (calcd for C_12_H_20_O_3_Na, 235.1305).

5*S*-hydroxyrecifeiolide (**2**): Colourless oil; ${\left[\alpha \right]}_{D}^{25}$ +23.07 (*c* 0.13, MeOH); UV (MeOH) *λ*_max_ (log *ε*) 205 (3.01), 220 (3.06); ECD (8.49 mM, MeOH) *λ*_max_ (Δ*ε*) 230 (−0.03) nm; ^1^H and ^13^C NMR data, see Table 1; ESIMS *m/z* 235 [M + Na]^+^; (+)-HRESIMS at *m/z* 235.1299 [M + Na]^+^ (calcd for C_12_H_20_O_3_Na, 235.1305).

*ent*-Cladospolide F (**3**): Colorless crystal (MeOH); mp 59–62 °C; ${\left[\alpha \right]}_{D}^{25}$ −29.41 (*c* 0.17, MeOH); UV (MeOH) *λ*_max_ (log *ε*) 206 (3.42); ECD (7.82 mM, MeOH) *λ*_max_ (Δ*ε*) 210 (+0.28) nm, 267 (+0.03) nm; ^1^H and ^13^C NMR data, see Table 2; ESIMS *m/z* 231 [M + H]^+^, *m/z* 253 [M + Na]^+^; (+)-HRESIMS at *m/z* 231.1589 [M + H]^+^ (calcd for C_12_H_23_O_4_, *m/z* 231.1591), at *m/z* 253.1407 [M + Na]^+^ (calcd for C_12_H_22_O_4_Na, *m/z* 253.1410).

Cladospolide G (**4**): Yellow oil; ${\left[\alpha \right]}_{D}^{25}$ –24.56 (*c* 0.57, MeOH); UV (MeOH) *λ*_max_ (log *ε*) 206 (3.49), 220 (3.27), 275 (2.62); ECD (4.04 mM, MeOH) *λ*_max_ (Δ*ε*) 207 (+0.80) nm, 323 (−0.10) nm; ^1^H and ^13^C NMR data, see Table 2; ESIMS *m/z* 273 [M + H]^+^, *m/z* 295 [M + Na]^+^; (+)-HRESIMS at *m/z* 273.1700 [M + H]^+^ (calcd for C_14_H_25_O_5_, *m/z* 273.1697), at *m/z* 290.1970 [M + NH_4_]^+^ (calcd for C_14_H_28_O_5_N, *m/z* 290.1962), at *m/z* 295.1515 [M + Na]^+^ (calcd for C_14_H_24_O_5_Na, *m/z* 295.1516).

Cladospolide H (**5**): pale yellow oil; ^1^H and ^13^C NMR data, see Table 2; ESIMS *m/z* 233 [M + Na]^+^; (+)-HRESIMS at *m/z* 233.1151 [M + Na]^+^ (calcd for C_12_H_18_O_3_Na, *m/z* 233.1148). (The optical rotation and ECD of **5** could not be detected due to the limited quantity).

*Iso*-cladospolide B (**6**): colorless crystal (MeOH); mp 105–112 °C; ${\left[\alpha \right]}_{D}^{25}$ −90.91 (*c* 0.11, MeOH); UV (MeOH) *λ*_max_ (log *ε*); ECD (9.21 mM, MeOH) *λ*_max_ (Δε) 213 (−5.69) nm; ^1^H and ^13^C NMR data, see Table 2; (+)-HRESIMS at *m/z* 229.1432 [M + H]^+^ (calcd for C_12_H_21_O_4_, 229.1434), at *m/z* 246.1699 [M + NH_4_]^+^ (calcd for C_12_H_24_O_4_N, 246.1700). 

### 3.5. X-Ray Crystallographic Analysis of Compounds ***3*** and ***6***

All crystallographic data were collected on an Agilent Xcalibur Eos Gemini CCD plate diffractometer, using graphite monochromatized Cu/K*α* radiation (λ= 1.54178 Å) [25]. The data were corrected for absorption by using the program SADABS [26]. The structures were solved by direct methods with the SHELXTL software package [27]. All nonhydrogen atoms were refined anisotropically. The H atoms were located by geometrical calculations, and their positions and thermal parameters were fixed during the structure refinement. The structure was refined by full-matrix least-squares techniques [28].

*Crystal data for compound***3***:* C_12_H_22_O_4_, F.W. = 230.30, Orthorhombic space group P2(1)2(1)2(1), unit cell dimensions *a* = 5.4655(4) Å, *b* = 5.5812(6) Å, *c* = 41.275(3) Å, *V* = 1259.06(19) Å^3^, *α* =*β* =*γ* = 90°, *Z* = 4, *d*_calcd_ = 1.215 mg/m^3^, crystal dimensions 0.40 × 0.28 × 0.10 mm^3^, *μ* = 0.734 mm^–^^1^, *F*(000) = 504. The 2385 measurements yielded 1827 independent reflections after equivalent data were averaged, and Lorentz and polarization corrections were applied. The final refinement gave *R*_1_ = 0.0487 and w*R*_2_ = 0.0970 (*I* > 2*σ*(*I*)). The Flack parameter was 0.0 (5) in the final refinement for all 1827 reflections with 147 Friedel pairs.

*Crystal data for compound***6***:* C_12_H_20_O_4_, F.W. = 228.13, Orthorhombic space group P2(1)2(1)2(1), unit cell dimensions *a* = 5.5217(5) Å, *b* = 7.6778(7) Å, *c* = 28.947(2) Å, *V* = 1227.17(19) Å^3^, *α* =*β* =*γ* = 90°, *Z* = 6, *d*_calcd_ = 1.236 mg/m^3^, crystal dimensions 0.35 × 0.24 × 0.16 mm^3^, *μ* = 0.752 mm^–^^1^, *F*(000) = 496. The 5282 measurements yielded 2081 independent reflections after equivalent data were averaged, and Lorentz and polarization corrections were applied. The final refinement gave *R*_1_ = 0.0727 and w*R*_2_ = 0.1620 (*I* > 2*σ*(*I*)). The Flack parameter was 0.5 (7) in the final refinement for all 2081 reflections with 150 Friedel pairs.

### 3.6. Acetylation of Compounds ***3*** and ***4***

To 5 μmol samples of compound **3** or **4** in glass-stoppered flask were added 400 μL dichloromethane, then excess amount of triethylamine was added. Drip 20 μmol of acetylchloride slowly into the flask in ice bath and keeping the reaction for 12 h. Then stop the reaction by adding 20 μL of water into the flask. The progress of the reaction was monitored by TLC analysis. The resulting reaction mixture was extracted with dichloromethane (2 × 400 μL), dried with Na_2_SO_4_, and concentrated in vacuo to obtain the product.

### 3.7. Antimicrobial Assay

Antimicrobial evaluation against two human pathogens (*Escherichia coli* EMBLC-1, *Staphylococcus aureus* EMBLC-2) and ten aquatic pathogens (*Aeromonas hydrophilia* QDIO-1, *Edwardsiella ictarda* QDIO-9, *E. tarda* QDIO-2, *Micrococcus luteus* QDIO-3, *Pseudomonas aeruginosa* QDIO-4, *Vibrio alginolyticus* QDIO-5, *V*. *anguillarum* QDIO-6, *V*. *harveyi* QDIO-7, *V*. *parahaemolyticus* QDIO-8, and *V*. *vulnificus* QDIO-10), as well as 15 plant-pathogenic fungi (*Alternaria solani* QDAU-1, *Bipolaris sorokiniana* QDAU-5, *Ceratobasidium cornigerum* QDAU-6, *Colletotrichum glecosporioides* QDAU-2, *Coniothyrium diplodiella* QDAU-7, *Fusarium graminearum* QDAU-4, *F. oxysporum* f. sp. *cucumerinum* QDAU-8, *F*. *oxysporum* f. sp. *momodicae* QDAU-9, *F*. *oxysporum* f. sp. *radicis lycopersici* QDAU-10, *F*. *solani* QDAU-11, *Glomerella cingulate* QDAU-12, *Helminthosporium maydis* QDAU-15, *Penicillium digitatum* QDAU-14, *Physalospora piricola* Nose QDAU-15, and *Valsa mali* QDAU-16), was carried out by the 96-well microtiter plates assay [29]. The pathogens were obtained from the Institute of Oceanology, Chinese Academy of Sciences. Chloramphenicol and amphotericin were used as positive controls for bacteria and fungi, respectively. All of the tested compounds and controls were dissolved in DMSO.

### 3.8. Enzyme inhibitory Assay

A modified Ellman’s method [30] was used to evaluate AChE inhibitory activities of compounds **1**–**7** in 96-well microplates. Tacrine was used as the standard inhibitor, and control test was performed without the presence of AChE inhibitors. All the inhibitors, solubilized in MeOH, were diluted stepwise from initial concentration of 32 μM. Every experiment was performed in triplicate. 5 μL inhibitor was added to each well and dried, then 50 μL phosphate buffer (PBS, 10 × 0.01 M, pH 7.2–7.4) was dispensed followed by 10 μL AChE (2 U/mL) and 20 μL 5,5-dithiobis 2-nitrobenzoic acid (DTNB, 5 mM). After 10 min culturing at 37 °C, 20 μL acetylthiocholine iodide (ATCh, 10 mM) was added and then OD was read at 405 nm over another period of 10 min culturing at 37 °C. The enzymatic inhibitory activity was calculated according to the following equation: Inhibition % = ((C − C_backgroud_) − (A − A_backgroud_))/(C − C_backgroud_) ×100%, where C is the OD value of the control and A is the OD value in the presence of the inhibitor. As for the background, ATCh was replaced by PBS in A and C and bovine albumin (BSA, 1mg/mL) took the place of AChE in C.

## 4. Conclusions

In summary, five new compounds (**1**–**5**) and two previously reported metabolites (**6** and **7**) were isolated from the mangrove-derived endophytic fungus *C. cladosporioides* MA-299. The structures of **3** and **6** were confirmed by single-crystal X-ray diffraction analysis and this is the first time for reporting the crystal structures of the two compounds. Compound **4** showed potent inhibitory activity against plant-pathogenic fungi (*G*. *cingulate* and *F.oxysporum* f. sp. *cucumerinum*), each with MIC value of 1.0 μg/mL, while **7** showed potent inhibitory activity against aquatic bacterium (*E. ictarda*) and plant-pathogenic fungus (*G*. *cingulate*), with MIC values of 4.0 and 1.0 μg/mL respectively. Compound **3** exhibited moderate inhibitory activity against human pathogenic bacterium *S. aureus* with MIC value of 8.0 μg/mL and acetylcholinesterase inhibitory activity with IC_50_ value of 40.26 μM.

## Figures and Tables

**Table 1:** ^1^H and ^13^C NMR data of **1**, **2**, and **7** (*δ* in ppm).

Pos.	1			2		7	
	*^a^ δ*_H_ (*J* in Hz)	*^b^ δ*_H_ (*J* in Hz)	*^d^ δ*_C_	*^b^ δ*_H_ (*J* in Hz)	*^e^ δ*_C_	*^c^ δ*_H_ (*J* in Hz)	*^f^ δ*_C_
1			172.3, C		172.2, C		174.7, C
2	*α* 2.40, ddd (13.0, 5.6, 3.9) *β* 1.93, ddd (13.1, 11.0, 4.4)	*α* 2.40, ddd (13.0, 5.6, 3.9) *β* 1.93, ddd (13.1, 11.0, 4.4)	35.2, CH_2_	*α* 2.25, m (overlap) *β* 2.16, m	31.8, CH_2_	*α* 3.23, dd (18.5, 8.5) *β* 2.90, dd (18.5, 1.7)	43.0, CH_2_
3	1.56, m (overlap)	1.56, m (overlap)	18.2, CH_2_	*α* 1.67, m (overlap) *β* 1.54, m	19.9, CH_2_	4.72, dd (8.5, 1.7)	65.7, CH
4	1.43, m (overlap)	1.43, m (overlap)	32.9, CH_2_	*α* 1.41, m *β* 0.97, m	30.0, CH_2_		209.3, C
5	3.51, m	3.51, m	70.2, CH	3.48, m	65.4, CH	4.31, d (5.4)	76.3, CH
6	*α* 1.60, m (overlap) *β* 1.35, m (overlap)	*α* 1.60, m (overlap) *β* 1.35, m (overlap)	32.6, CH_2_	*α* 1.63, m (overlap) *β* 1.30, m	33.7, CH_2_	*α* 1.97, m *β* 1.76, m	30.7, CH_2_
7	*α* 2.17, m *β* 1.72, m	*α* 2.17, m *β* 1.72, m	28.6, CH_2_	*α* 2.05, m *β* 1.91, m	27.7, CH_2_	*α* 1.45, m (overlap) *β* 1.18, m (overlap)	20.8, CH_2_
8	5.34, ddd (15.3, 10.7, 2.8)	5.34, ddd (15.3, 10.7, 2.8)	135.2, CH	5.26, m	132.7, CH	*α* 1.60, m (overlap) *β* 1.31, m (overlap)	26.9, CH_2_
9	5.20, ddd (15.3, 10.1, 4.1)	5.20, ddd (15.3, 10.1, 4.1)	125.6, CH	5.11, m	126.7, CH	*α* 1.51, m (overlap) *β* 1.10, m (overlap)	22.7, CH_2_
10	*α* 2.25, m *β* 2.06, m	*α* 2.25, m *β* 2.06, m	40.3, CH_2_	*α* 2.29, m (overlap) *β* 1.99, m (overlap)	40.3, CH_2_	*α* 1.69, m (overlap) *β* 1.39, m (overlap)	33.5, CH_2_
11	4.84, m	4.84, m	68.3, CH	5.02, m	67.8, CH	4.88, m	75.2, CH
12	1.17, d (6.3)	1.17, d (6.3)	20.4, CH_3_	1.15, d (6.3)	20.2, CH_3_	1.25, d (6.2)	19.3, CH_3_
3-OH						3.19, s	
5-OH	4.39, brs	4.39, brs		4.28, brs		3.03, s	

*^a^* Measured at 600 MHz in DMSO-*d*_6_. *^b^* Measured at 500 MHz in DMSO-*d*_6_. *^c^* Measured at 500 MHz in CDCl_3_. *^d^* Measured at 150 MHz in DMSO-*d*_6_. *^e^* Measured at 125 MHz in DMSO-*d*_6_. *^f^* Measured at 125 MHz in CDCl_3_.

**Table 2:** ^1^H and ^13^C NMR data of **3**–**6** (*δ* in ppm).

Pos.	3				4		5		6	
	*^a^ δ*_H_ (*J* in Hz)	*^b^ δ*_H_ (*J* in Hz)	*^d^ δ*_C_	*^e^ δ*_C_	*^c^ δ*_H_ (*J* in Hz)	*^f^ δ*_C_	*^a^ δ*_H_ (*J* in Hz)	*^d^ δ*_C_	*^a^ δ*_H_ (*J* in Hz)	*^d^ δ*_C_
1			175.5, C	175.8, C		175.5, C		169.7, C		173.2, C
2	*α* 2.85, dd (17.7, 6.4) *β* 2.25, dd (17.7, 3.2)	*α* 2.78, dd (18.0, 6.7) *β* 2.47, dd (18.0, 3.7)	37.0, CH_2_	37.8, CH_2_	*α* 2.85, dd (17.7, 6.4) *β* 2.25, dd (17.7, 3.2)	37.0, CH_2_	6.37, d (5.4)	118.4, CH	6.21, d (5.6)	121.1, CH
3	4.09, m	4.22, m	70.1, CH	71.7, CH	4.09, m	70.1, CH	7.80, d (5.4)	145.1, CH	7.71, d (5.6)	156.6, CH
4	4.18, m	4.31, ddd (8.3, 5.3, 3.0)	87.5, CH	88.2, CH	4.18, ddd (8.1, 5.5, 2.5)	87.5, CH		149.4, C	5.04, d (1.5)	86.2, CH
5	*α* 1.56, m *β* 1.48, m	1.57, m	32.1, CH_2_	33.2, CH_2_	*α* 1.57, m *β* 1.48, m (overlap)	32.0, CH_2_	5.53, t (7.9)	117.1, CH	3.66, m	69.4, CH
6	1.26, m (overlap)	1.34, m (overlap)	25.2, CH_2_	25.7, CH_2_	1.25, m (overlap)	24.7, CH_2_	2.30, m	25.8, CH_2_	1.44, m (overlap)	40.0, CH_2_
7	1.33, m (overlap)	1.41, m (overlap)	29.0, CH_2_	29.5, CH_2_	1.33, m (overlap)	28.6, CH_2_	1.44, m	28.3, CH_2_	1.34, m (overlap)	32.8, CH_2_
8	1.31, m (overlap)	1.28, m (overlap)	28.8, CH_2_	29.3, CH_2_	1.31, m (overlap)	28.5, CH_2_	1.23, m (overlap)	28.7, CH_2_	1.29, m (overlap)	29.1, CH_2_
9	1.24, m (overlap)	1.26, m (overlap)	24.8, CH_2_	25.3, CH_2_	1.24, m (overlap)	24.6, CH_2_	1.27, m (overlap)	25.0, CH_2_	1.25, m (overlap)	25.3, CH_2_
10	1.36, m (overlap)	1.45, m (overlap)	39.0, CH_2_	39.3, CH_2_	1.45, m (overlap)	35.2, CH_2_	1.32, m (overlap)	38.9, CH_2_	1.39, m (overlap)	39.0, CH_2_
11	3.55, m	3.75, m	65.7, CH	68.3, CH	4.78, m	70.1, CH	3.55, m (overlap)	65.6, CH	3.55, m	65.7, CH
12	1.02, d (6.1)	1.15, d (6.2)	23.6, CH_3_	23.7, CH_3_	1.15, d (6.3)	19.7, CH_3_	1.02, d (6.1)	23.6, CH_3_	1.02, d (6.2)	23.6, CH_3_
13						169.9, C				
14					1.97, s	21.0, CH_3_				
3-OH	5.49, s				5.50, d (4.0)					
5-OH									4.95, d (6.3)	
11-OH	4.27, d (4.2)						3.41, brs		4.29, d (4.7)	

*^a^* Measured at 500 MHz in DMSO-*d*_6_. *^b^* Measured at 500 MHz in CDCl_3_. *^c^* Measured at 600 MHz in DMSO-*d*_6_. *^d^* Measured at 125 MHz in DMSO-*d*_6_. *^e^* Measured at 125 MHz in CDCl_3_. *^f^* Measured at 150 MHz in DMSO-*d*_6_.

**Table 3:** Antimicrobial Activities of **1**–**7** (MIC, μg/mL) ^a^.

Strains	Compounds						
	1	2	3	4	6	7	Positive control
***E. coli*^b^**	–	–	–	32	32	–	2.0
***S. aureus*^b^**	–	–	8.0	–	–	32	1.0
***E. tarda*^b^**	–	–	–	–	32	–	0.5
***E. ictarda*^b^**	32	–	16	–	16	4.0	0.5
***G. cingulate*^c^**	–	16	–	1.0	64	1.0	0.5
***B. sorokiniana*^c^**	–	–	–	32	–	–	0.5
***P. aeruginosa*^c^**	32	–	64	–	–	32	2.0
***F.oxysporum* f. sp. *Cucumerinum*^c^**	–	–	–	1.0	–	–	0.5

*^a^* (–) = MIC > 64 *μ*g/mL, *^b^* Chloramphenicol as positive control, *^c^* Amphotericin B as positive control.

## Supplementary Materials

The following are available online at https://www.mdpi.com/1660-3397/17/5/296/s1, 1D and 2D NMR spectra and ECDs of compounds **1**–**5** as well as crystal packing of compounds **3** and **6**.
