# Supplementary material for: Polyketides from the Mangrove-Derived Endophytic Fungus Cladosporium cladosporioides
Source: Mar Drugs. 2019 May 17;17(5):296. doi: 10.3390/md17050296 (PMC6563031; doi:10.3390/md17050296)

Supplementary Material

Polyketides from the Mangrove-Derived Endophytic Fungus *Cladosporium cladosporioides*

Fan-Zhong Zhang [1,2,3], Xiao-Ming Li [1,2], Xin Li [1,2], Sui-qunYang [1,2], Ling-Hong Meng [*,1,2], and Bin-Gui Wang [*,1,2,3]

[1] Key Laboratory of Experimental Marine Biology, Center for Ocean Mega-Science, Institute of Oceanology, Chinese Academy of Sciences, Nanhai Road 7, Qingdao 266071, People's Republic of China; E-Mails: fancyzfz@163.com (F.-Z.Z.); lixmqdio@126.com (X.-M.L.); lixin871014@163.com (X.L.); suiqunyang@163.com (S.-Q.Y.)

[2] Laboratory of Marine Biology and Biotechnology, Qingdao National Laboratory for Marine Science and Technology, Wenhai Road 1, Qingdao 266237, People's Republic of China

[3] University of Chinese Academy of Sciences, Yuquan Road 19A, Beijing 100049, People's Republic of China

Corresponding Author

* Phone (B.-G. Wang): +86-532-82898553. E-mail: m8545303@163.com (L.-H.M.); wangbg@ms.qdio.ac.cn. (B.-G. Wang);

Content

Figure S1. HRESI mass spectrum of compound **1**;

Figure S2. ^{1}H NMR (600 MHz, DMSO-d_6) spectrum of compound **1**;

Figure S3. ^{1}H NMR (500 MHz, DMSO-d_6) spectrum of compound **1**;

Figure S4. ^{13}C NMR (125 MHz, DMSO-d_6) and DEPT spectra of compound **1**;

Figure S5. COSY spectrum of compound **1**;

Figure S6. HSQC spectrum of compound **1**;

Figure S7. HMBC spectrum of compound **1**;

Figure S8. NOESY spectrum of compound **1**;

Figure S9. ^{1}H NMR (500 MHz, DMSO-d_6) spectrum of (*S*)-MTPA ester (**1a**);

Figure S10. ^{1}H NMR (500 MHz, DMSO-d_6) spectrum of (*R*)-MTPA ester (**1b**);

Figure S11. HRESI mass spectrum of compound **2**;

Figure S12. ^{1}H NMR (500 MHz, DMSO-d_6) spectrum of compound **2**;

Figure S13. ^{13}C NMR (125 MHz, DMSO-d_6) and DEPT spectra of compound **2**;

Figure S14. COSY spectrum of compound **2**;

Figure S15. HSQC spectrum of compound **2**;

Figure S16. HMBC spectrum of compound **2**;

Figure S17. NOESY spectrum of compound **2**;

Figure S18. HRESI mass spectrum of compound **3**;

Figure S19. ^{1}H NMR (500 MHz, DMSO-d_6) spectrum of compound **3**;

Figure S20. ^{1}H NMR (500 MHz, $CDCl_3$) spectrum of compound **3**;

Figure S21. ^{13}C NMR (125 MHz, DMSO-d_6) and DEPT spectra of compound **3**;

Figure S22. ^{13}C NMR (125 MHz, $CDCl_3$) and DEPT spectra of compound **3**;

Figure S23. COSY spectrum of compound **3**;

Figure S24. HSQC spectrum of compound **3**;

Figure S25. HMBC spectrum of compound **3**;

Figure S26. ECD spectrum of compound **3**;

Figure S27. HRESI mass spectrum of compound **4**;

Figure S28. ^{1}H NMR (500 MHz, DMSO-d_6) spectrum of compound **4**;

Figure S29. ^{13}C NMR (125 MHz, DMSO-d_6) and DEPT spectra of compound **4**;

Figure S30. COSY spectrum of compound **4**;

Figure S31. HSQC spectrum of compound **4**;

Figure S32. HMBC spectrum of compound **4**;

Figure S33. ECD spectrum of compound **4**;
Figure S34. HRESI mass spectrum of compound **5**;
Figure S35. ^{1}H NMR (500 MHz, DMSO-d_6) spectrum of compound **5**;
Figure S36. ^{13}C NMR (125 MHz, DMSO-d_6) and DEPT spectra of compound **5**;
Figure S37. COSY spectrum of compound **5**;
Figure S38. HSQC spectrum of compound **5**;
Figure S39. HMBC spectrum of compound **5**;
Figure S40. NOESY spectrum of compound **5**;
Figure S41. HRESI mass spectrum of compound **6**;
Figure S42. ^{1}H NMR (500 MHz, DMSO-d_6) spectrum of compound **6**;
Figure S43. ^{13}C NMR (125 MHz, DMSO-d_6) and DEPT spectra of compound **6**;
Figure S44. COSY spectrum of compound **6**;
Figure S45. HMBC spectrum of compound **6**;
Figure S46.Crystal packing of compound **3**；
Figure S47.Crystal packing of compound **6**.

Figure S1. HRESI mass spectrum of compound **1**;

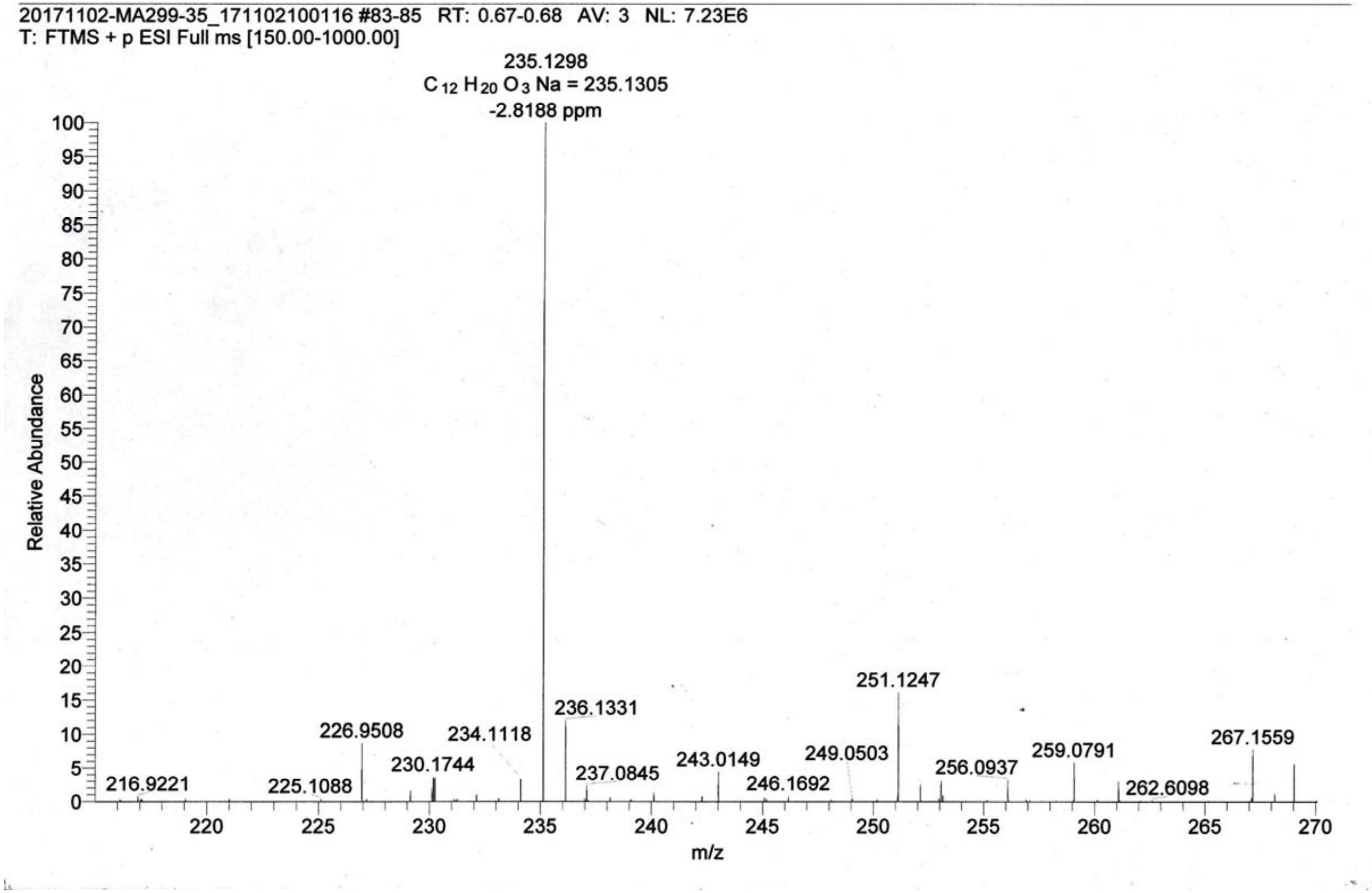

Figure S2. ^{1}H NMR (600 MHz, DMSO-d_6) spectrum of compound **1**;

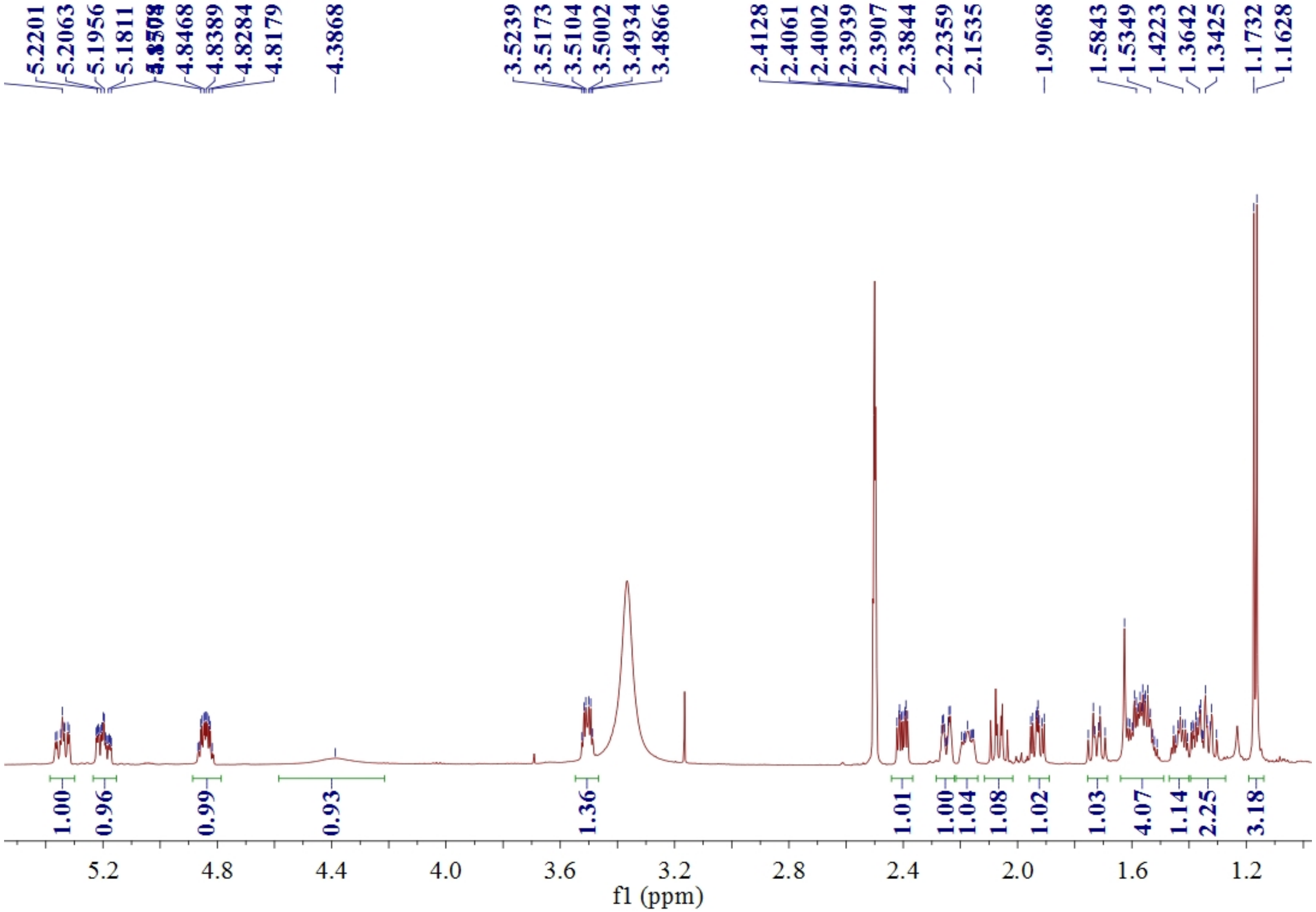

Figure S3. ^{1}H NMR (500 MHz, DMSO-d_6) spectrum of compound **1**;

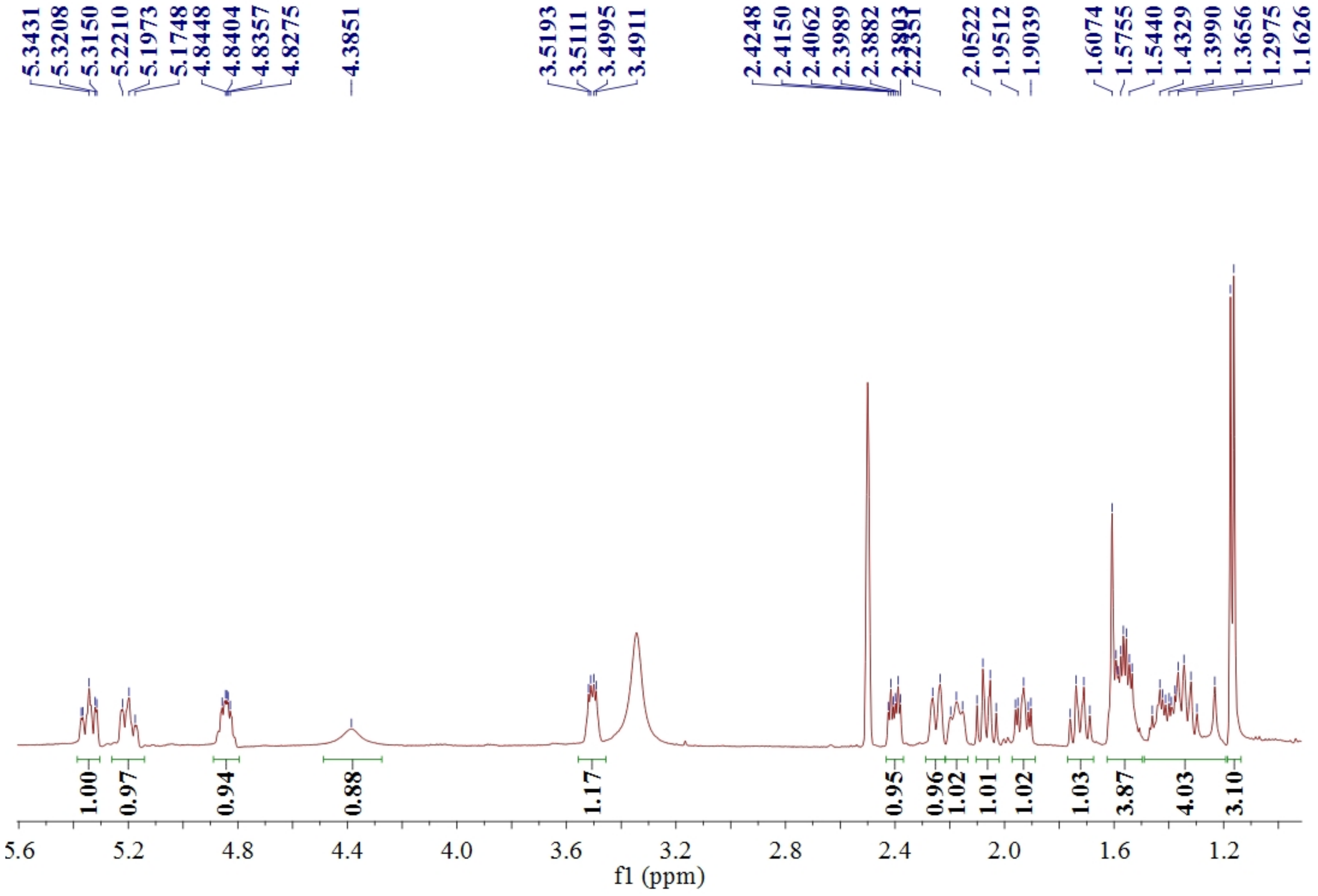

Figure S4. ^{13}C NMR (125 MHz, DMSO-d_6) and DEPT spectra of compound **1**;

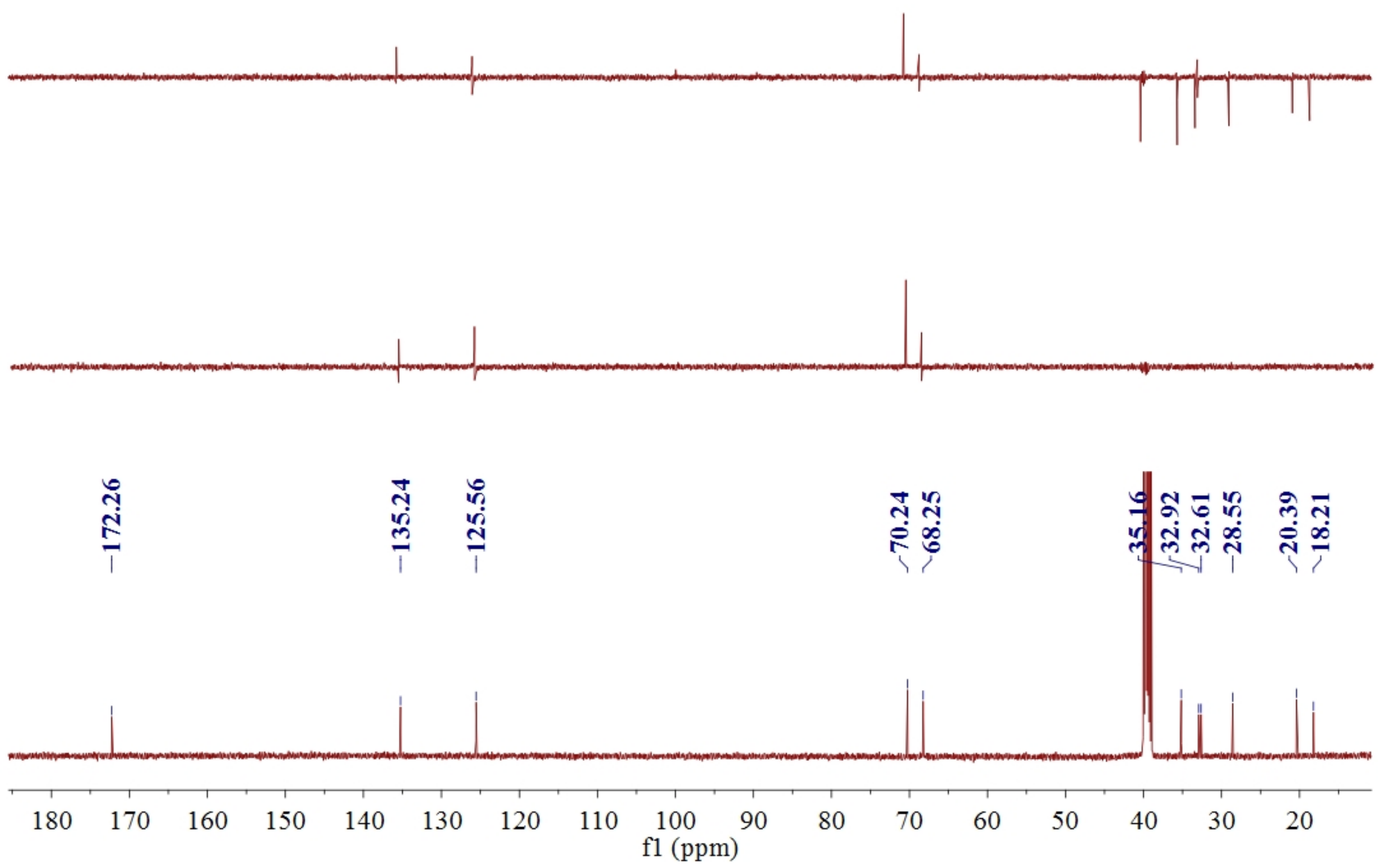

Figure S5. COSY spectrum of compound **1**;

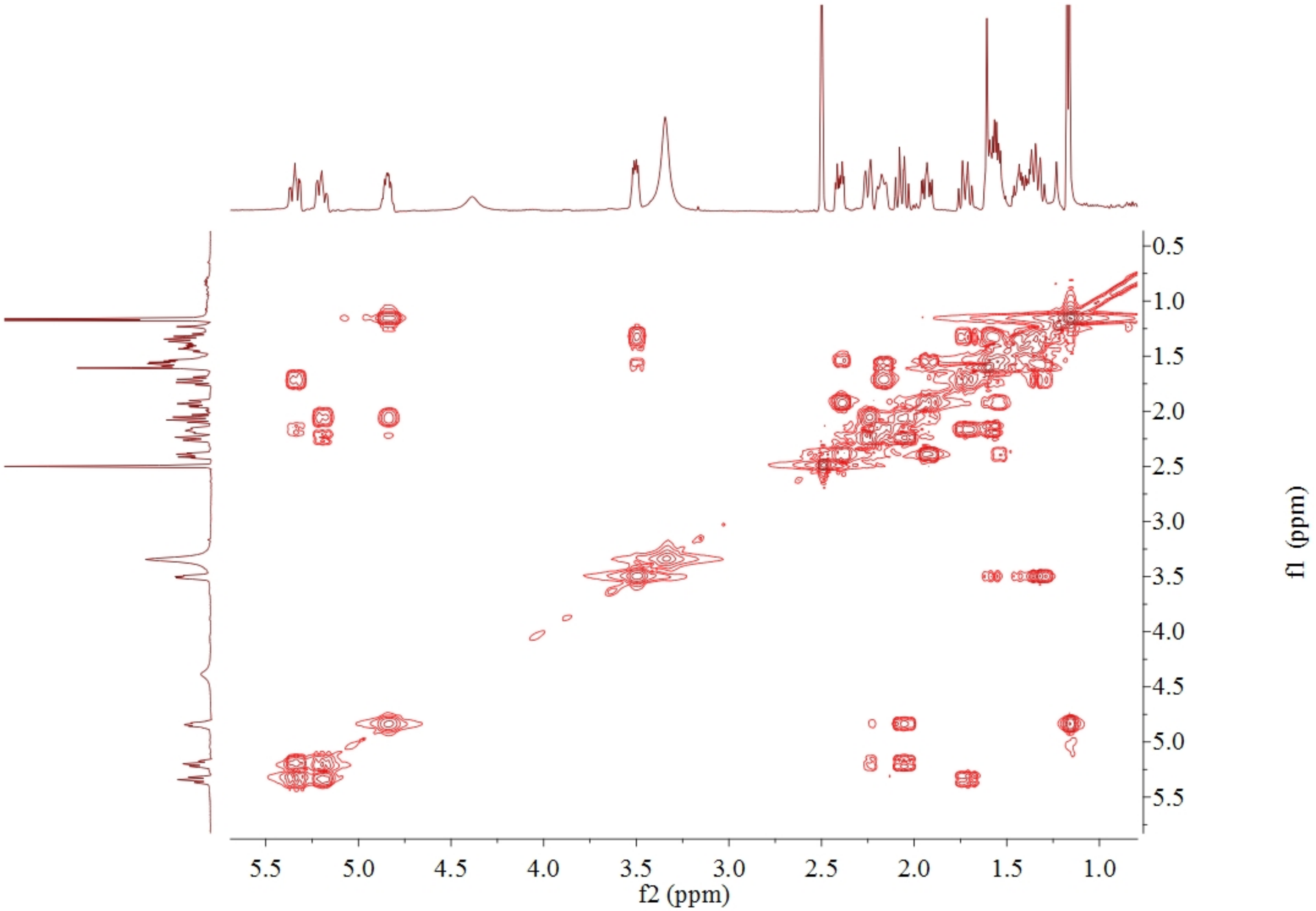

Figure S6. HSQC spectrum of compound **1**;

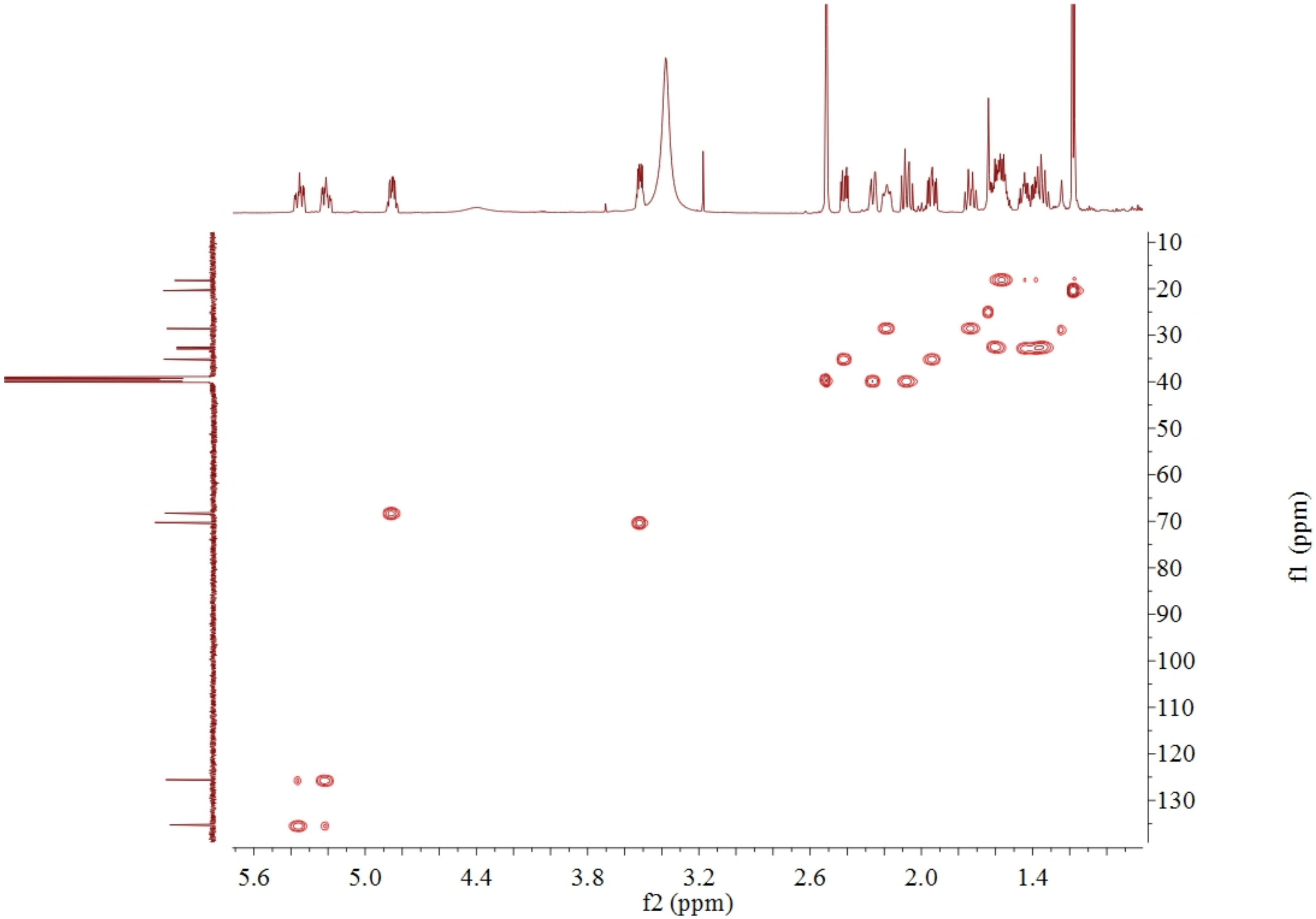

Figure S7. HMBC spectrum of compound **1**;

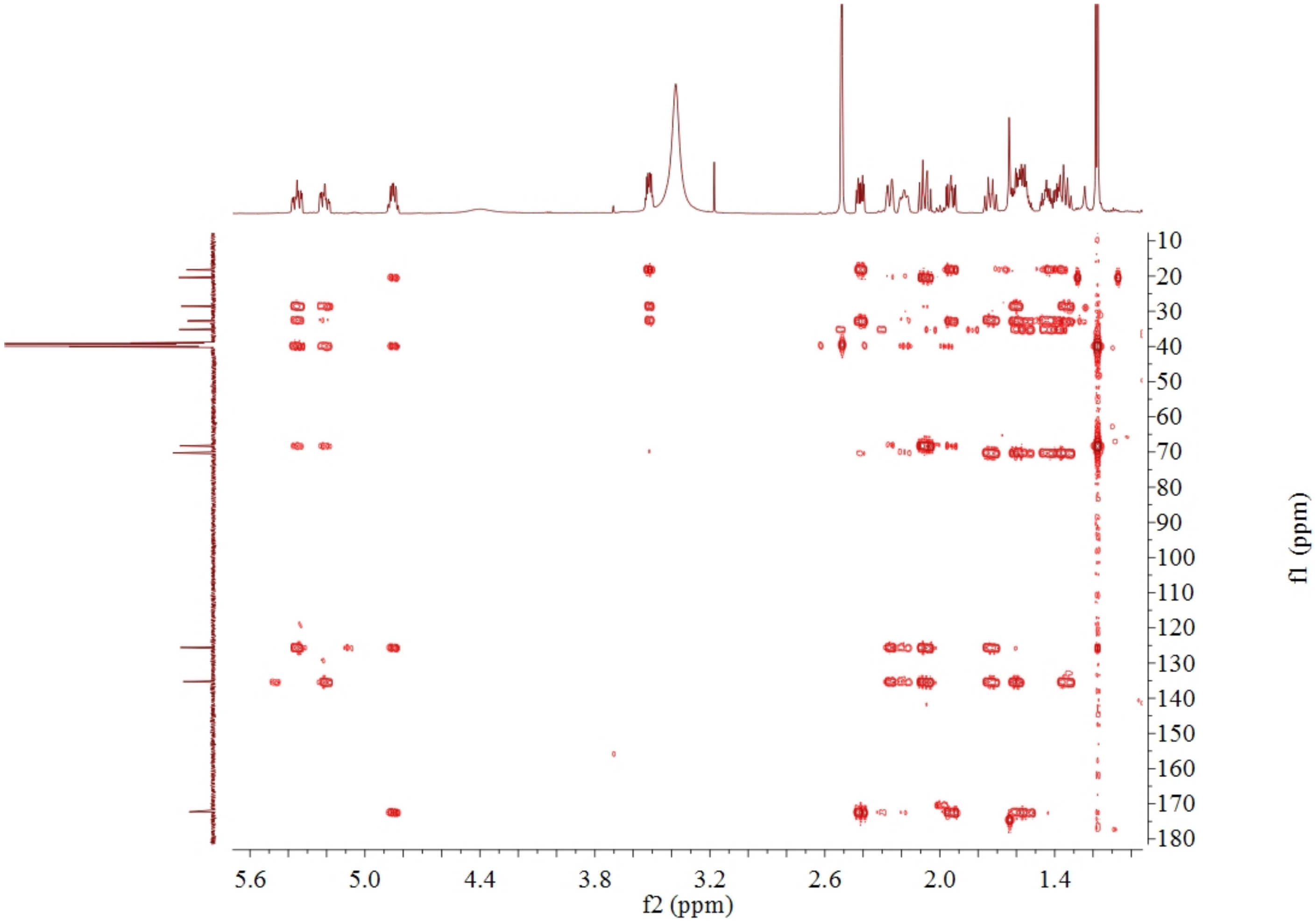

Figure S8. NOESY spectrum of compound **1**;

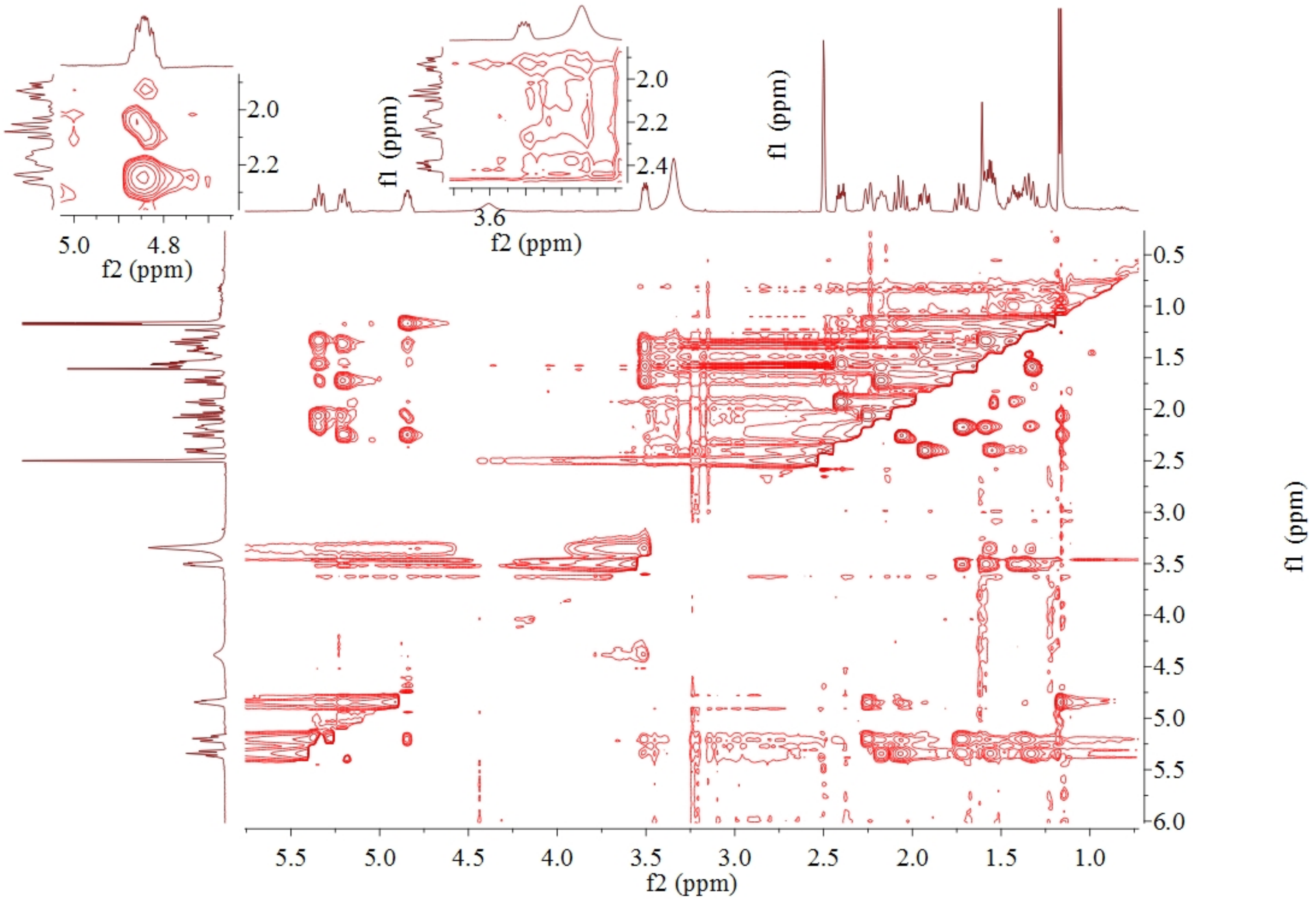

Figure S9. ^{1}H NMR (500 MHz, DMSO-d_6) spectrum of (*S*)-MTPA ester (**1a**);

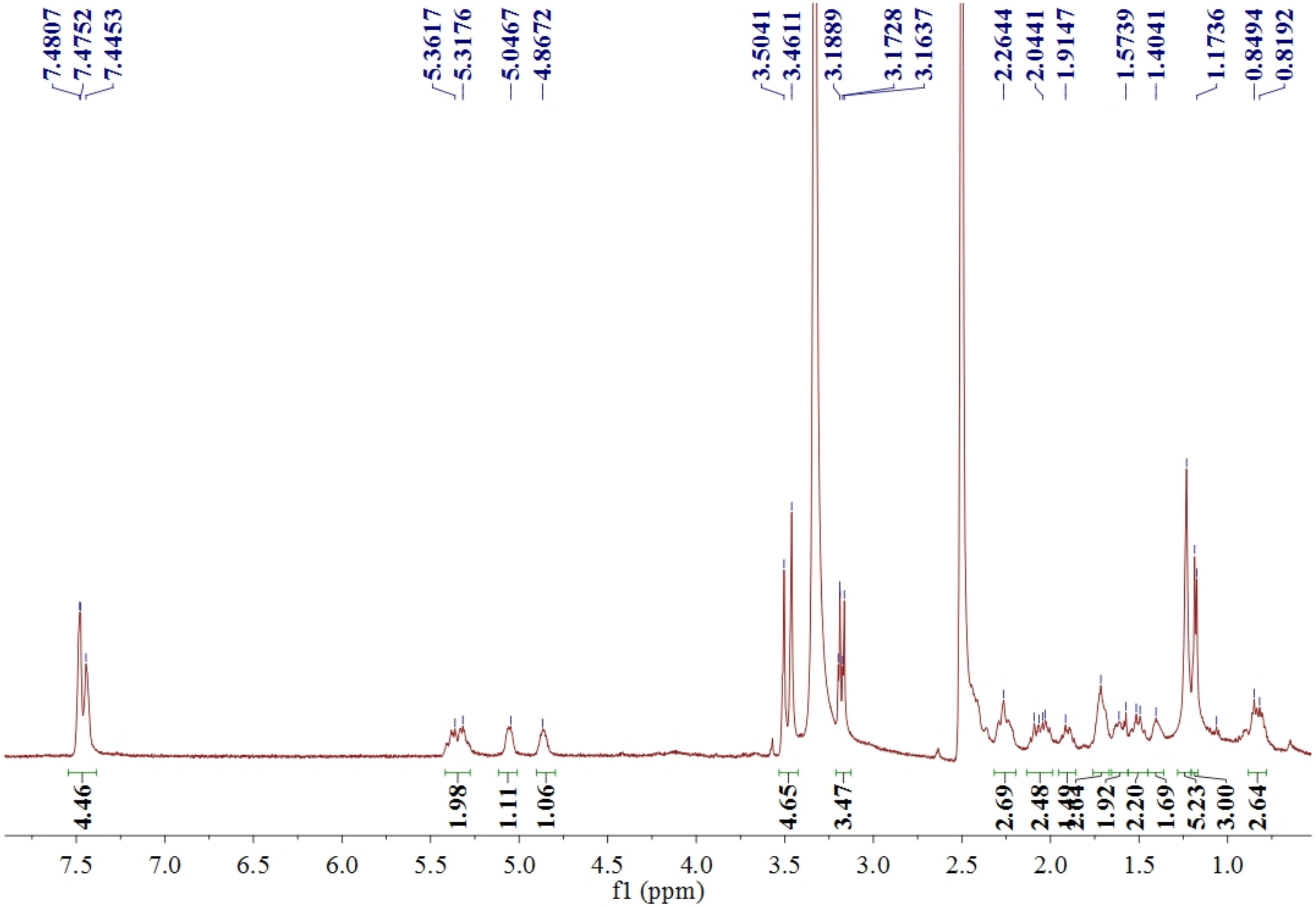

Figure S10. ^{1}H NMR (500 MHz, DMSO-d_6) spectrum of (*R*)-MTPA ester (**1b**);

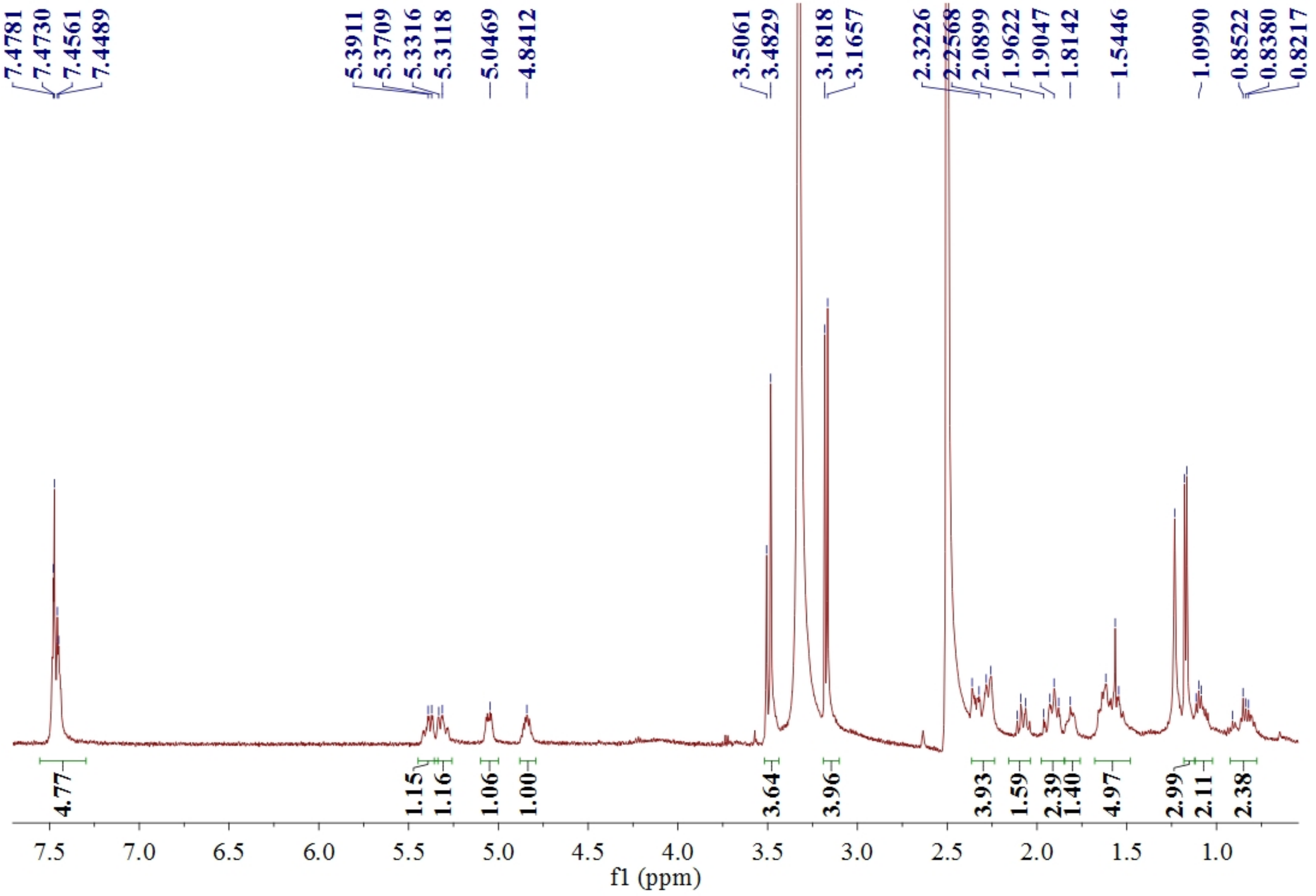

Figure S11. HRESI mass spectrum of compound **2**;

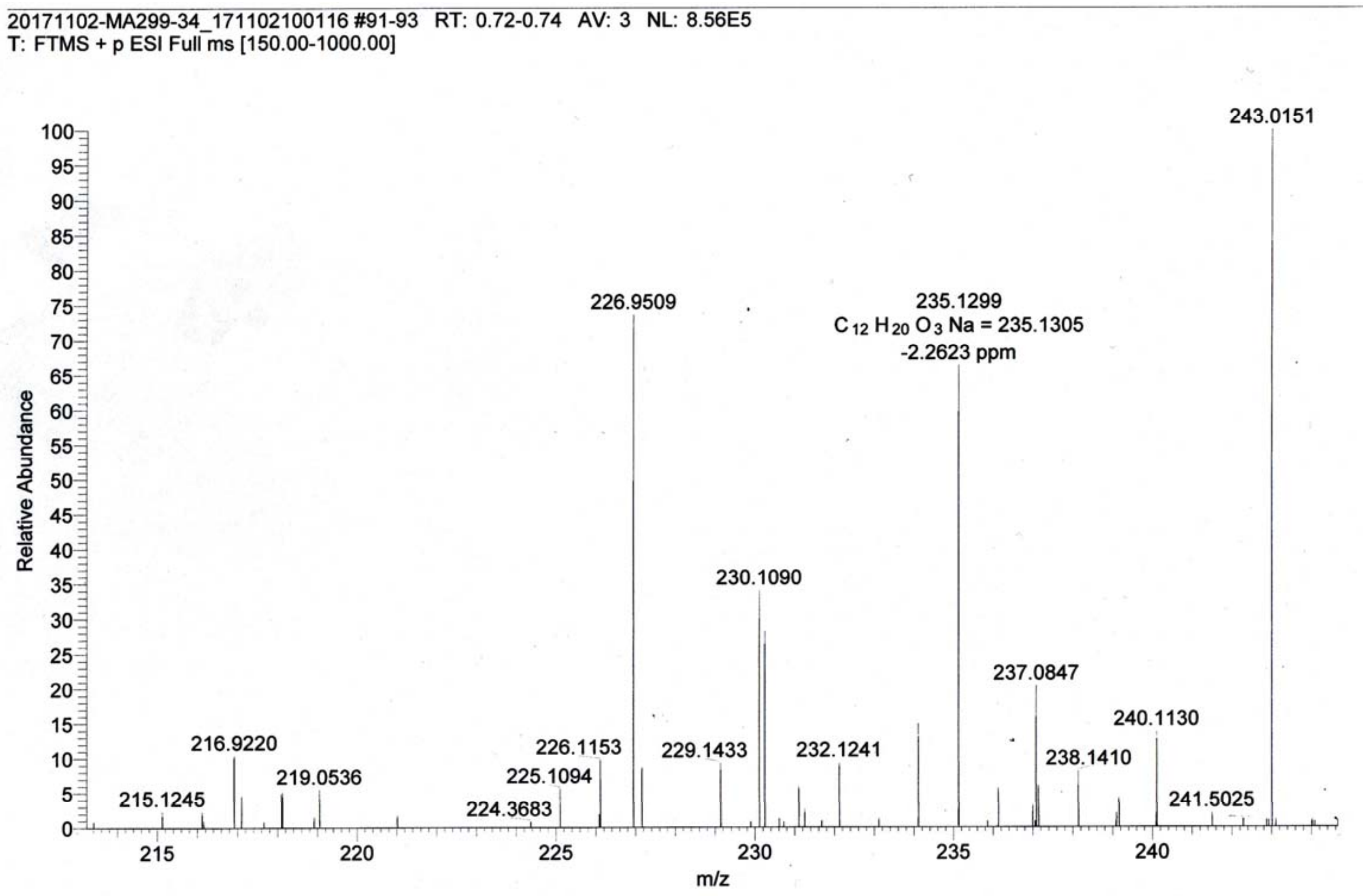

Figure S12. ^{1}H NMR (500 MHz, DMSO-d_6) spectrum of compound **2**;

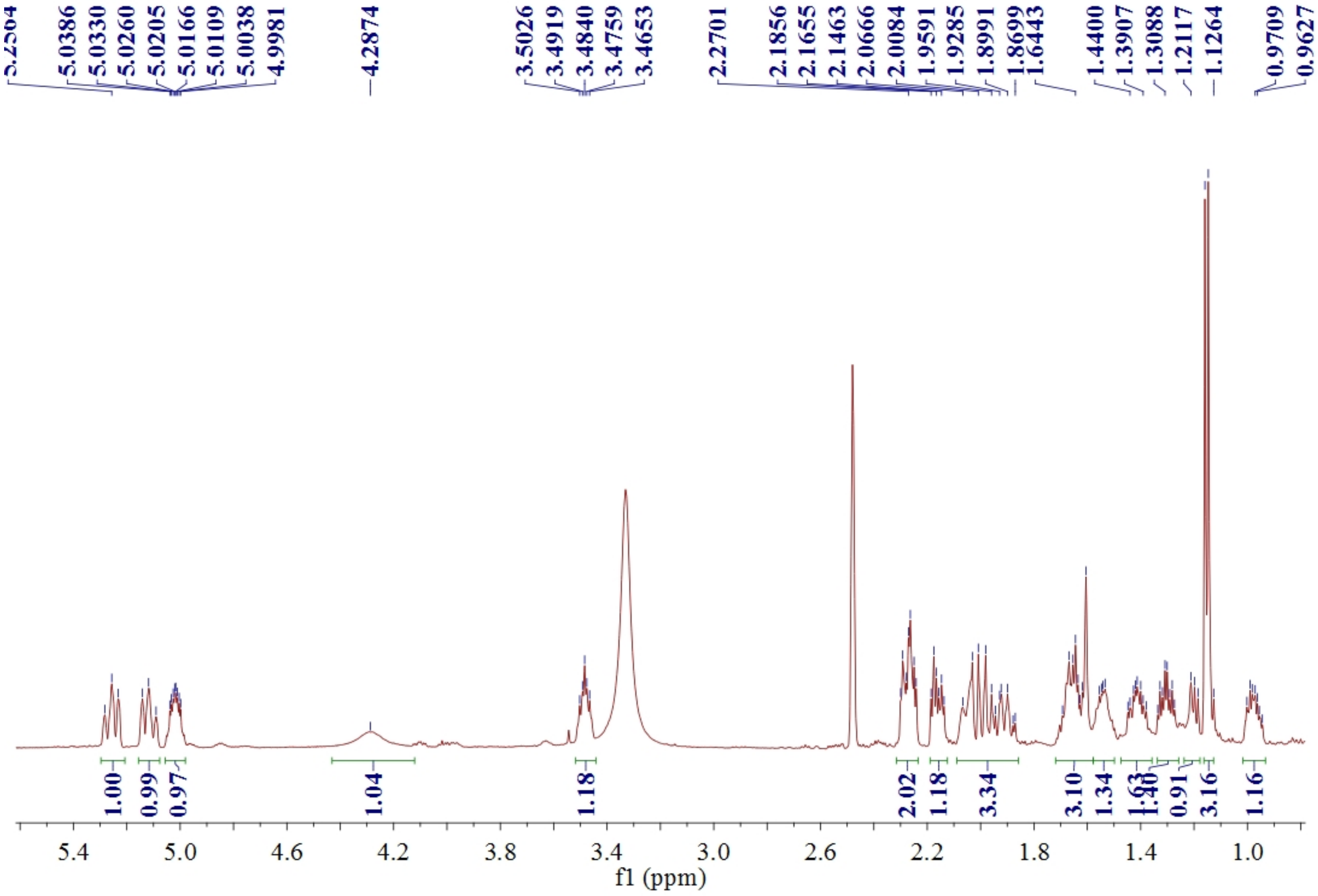

Figure S13. ^{13}C NMR (125 MHz, DMSO-d_6) and DEPT spectra of compound **2**;

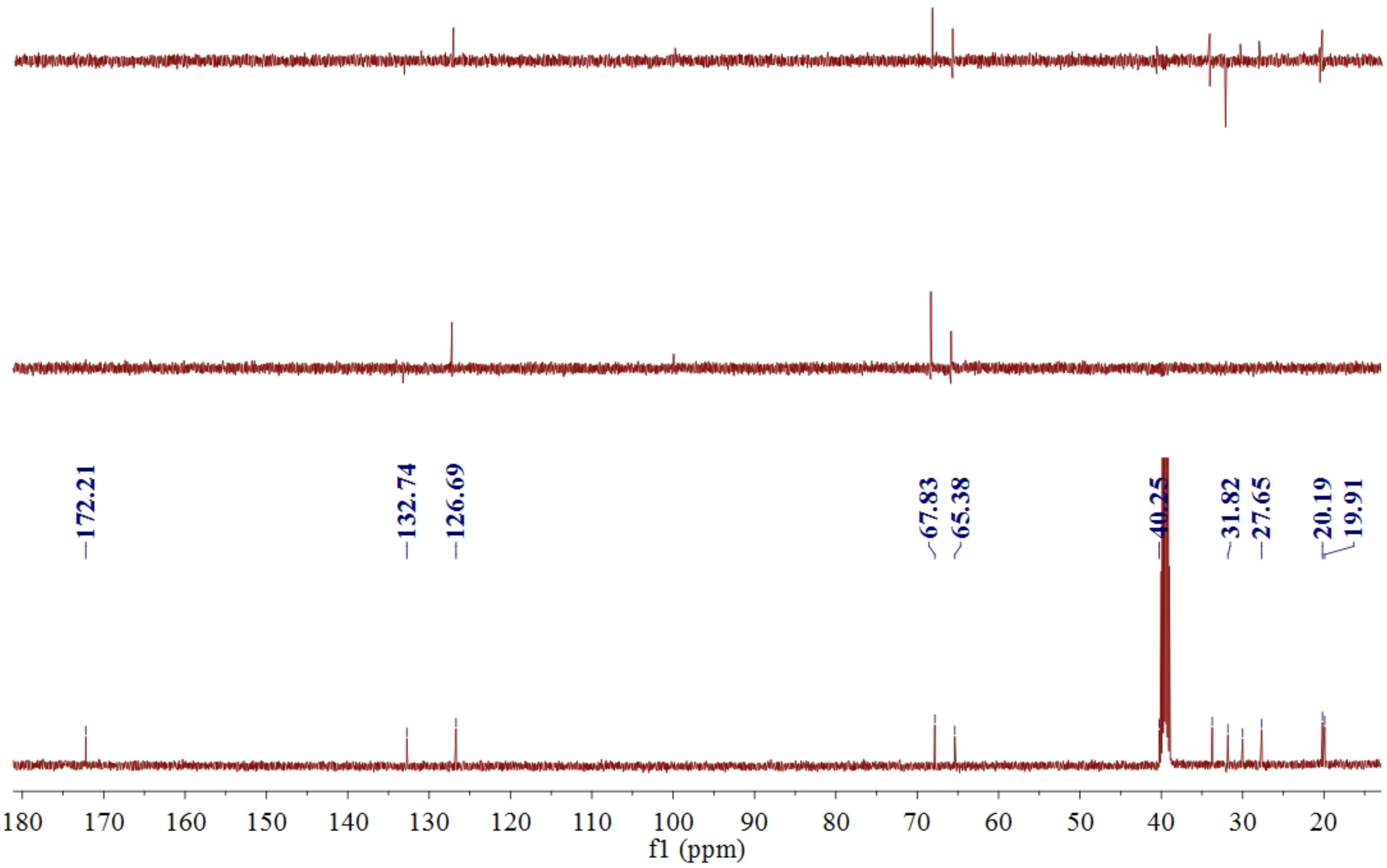

Figure S14. COSY spectrum of compound **2**;

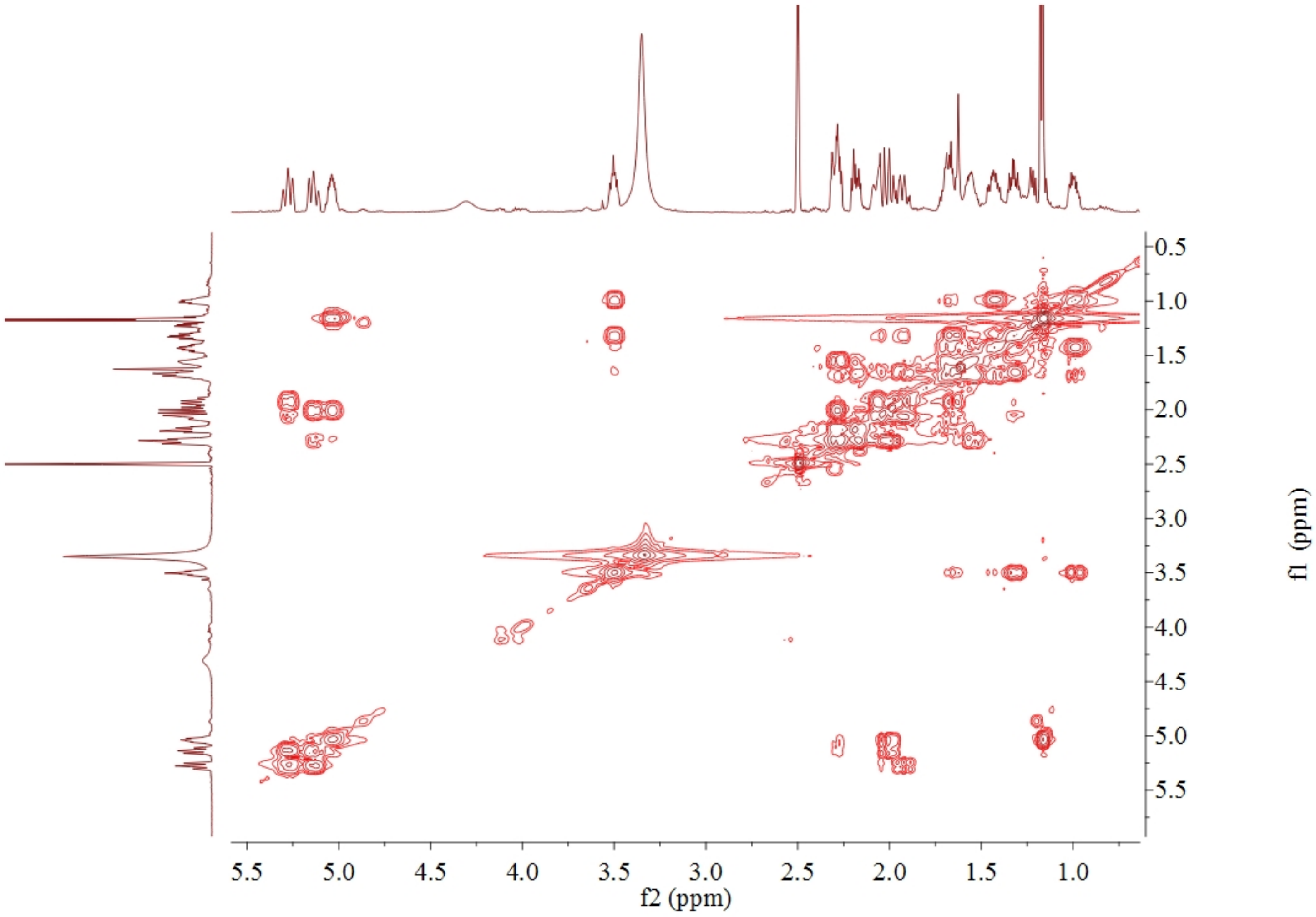

Figure S15. HSQC spectrum of compound **2**;

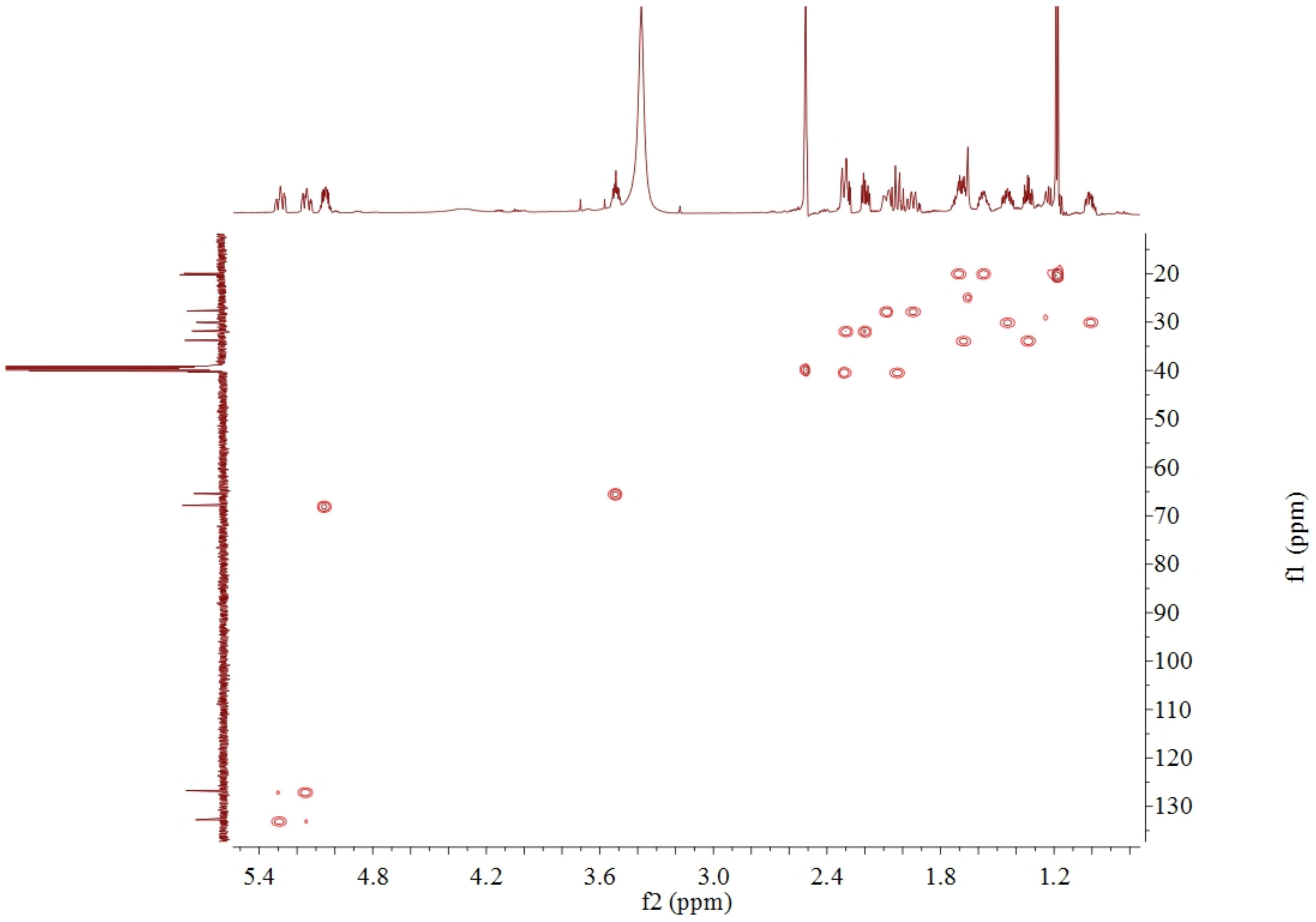

Figure S16. HMBC spectrum of compound **2**;

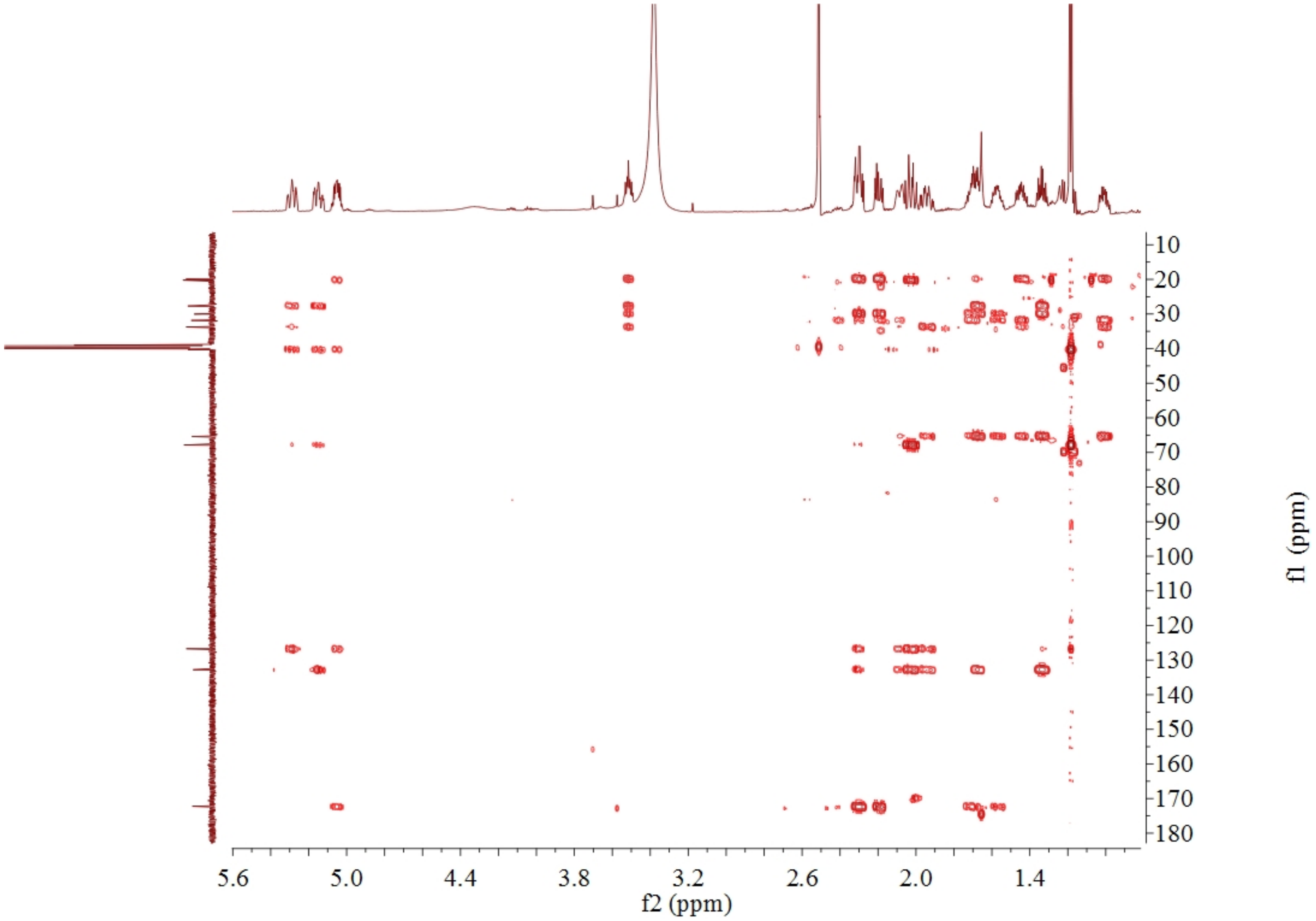

Figure S17. NOESY spectrum of compound **2**;

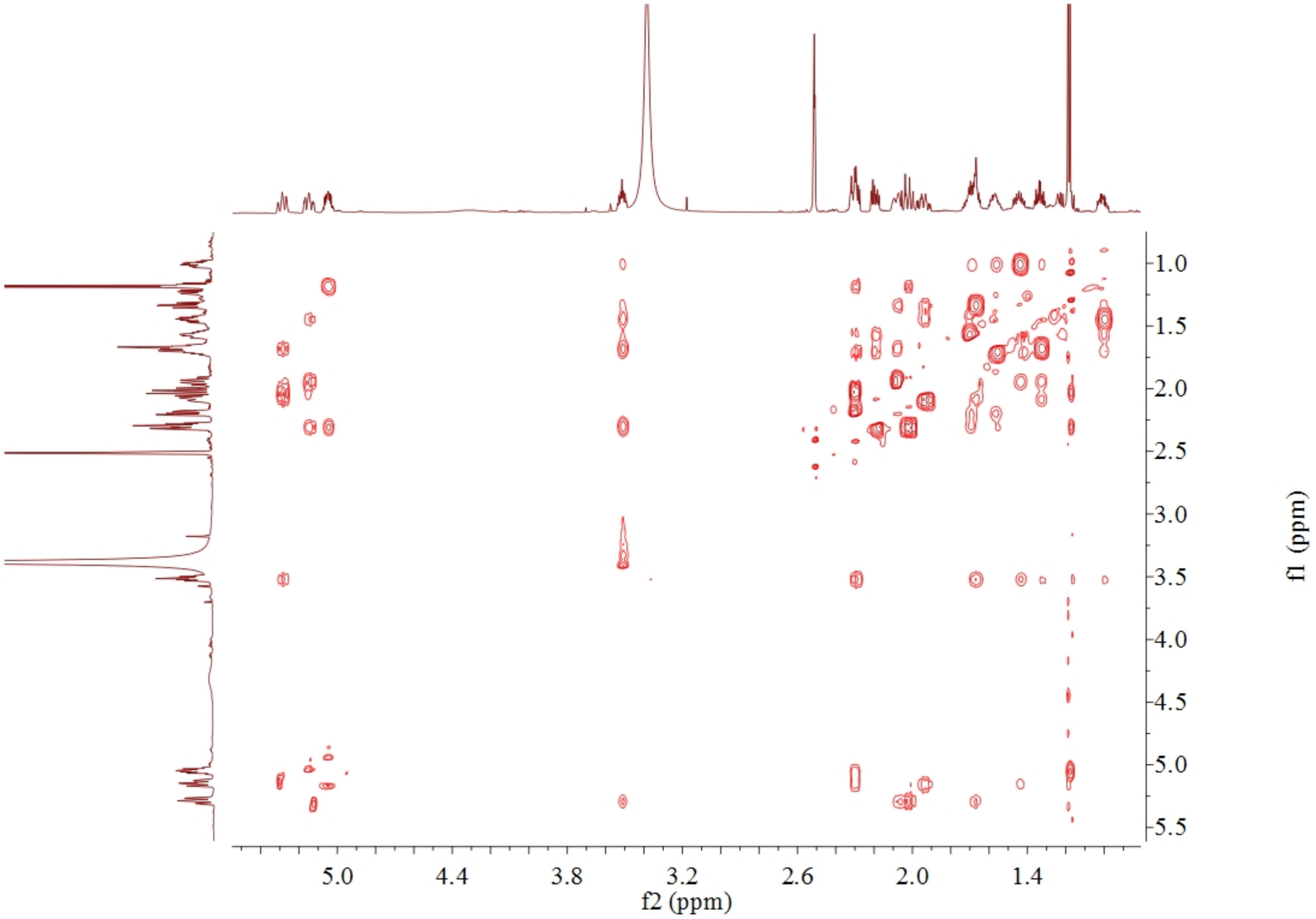

Figure S18. HRESI mass spectrum of compound **3**;

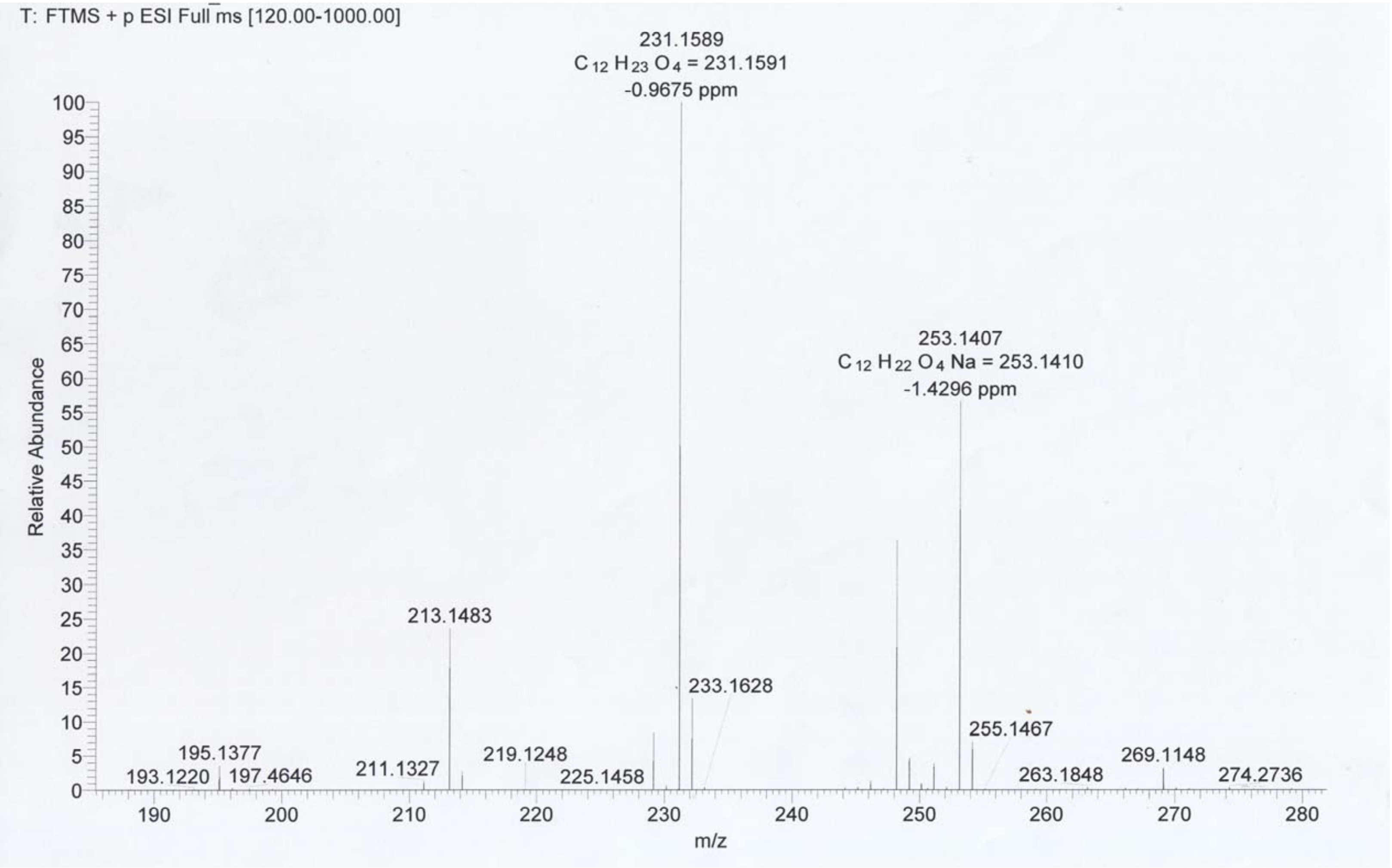

Figure S19. ^{1}H NMR (500 MHz, DMSO-d_6) spectrum of compound **3**;

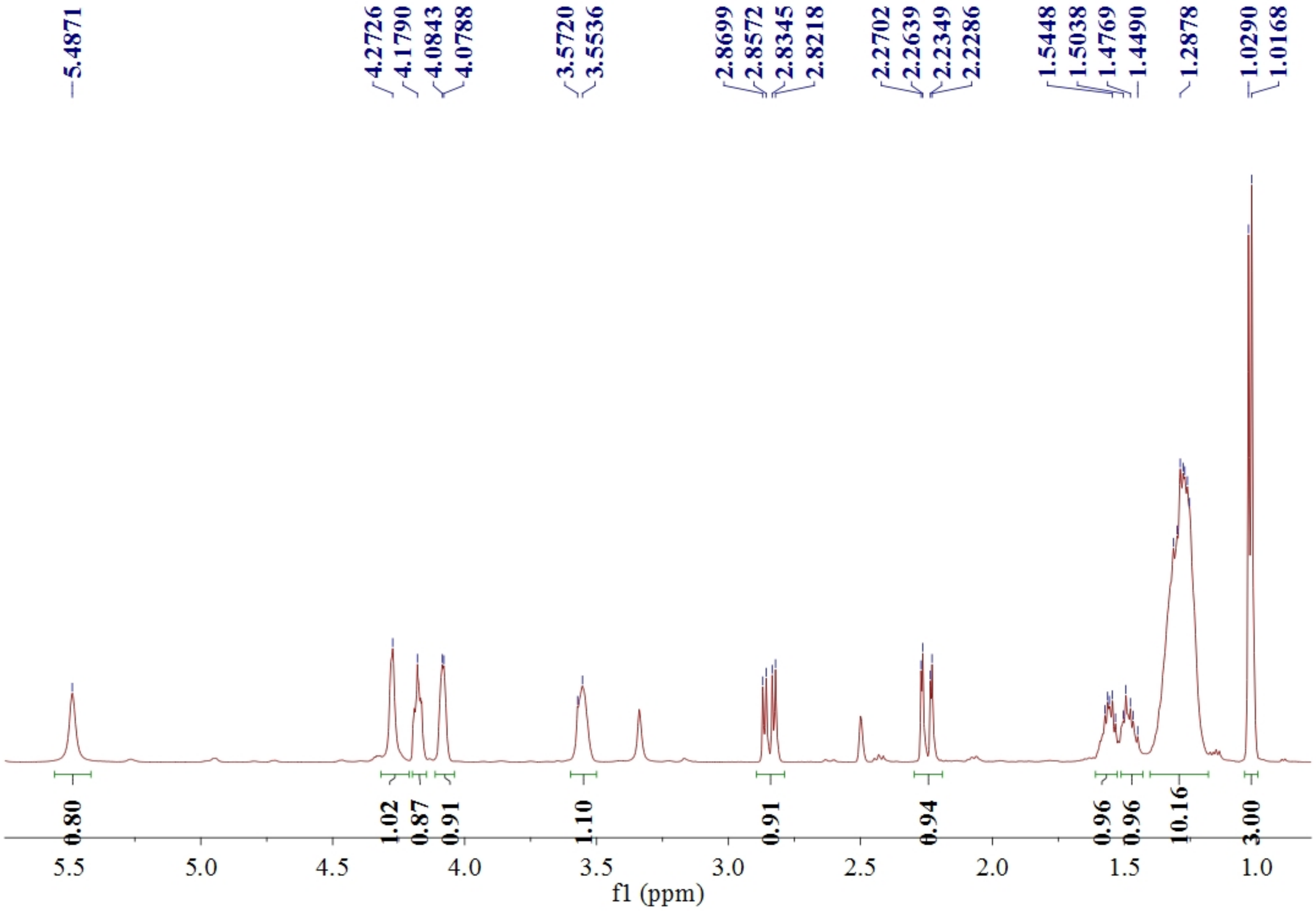

Figure S20. ^{1}H NMR (500 MHz, $CDCl_3$) spectrum of compound **3**;

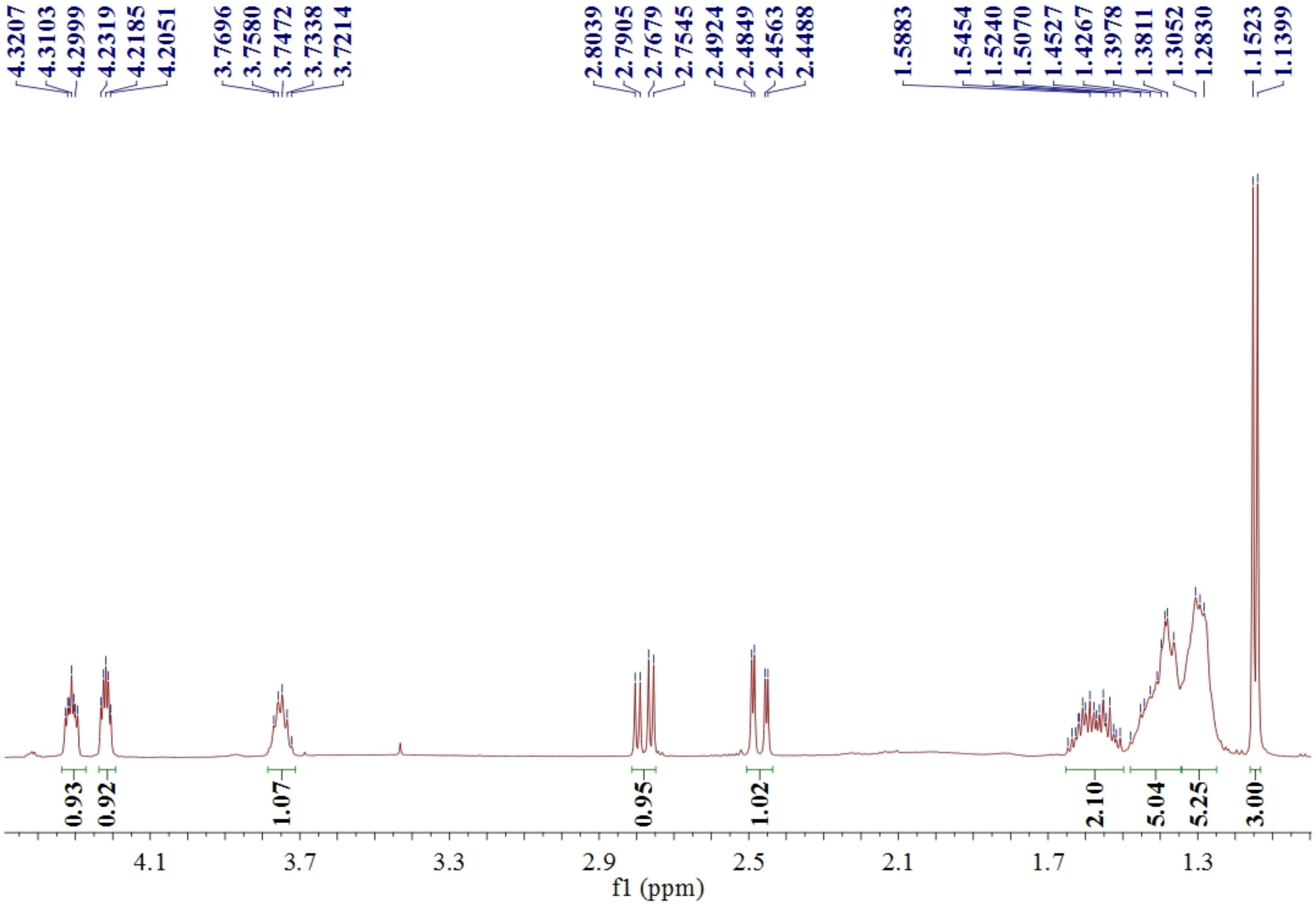

Figure S21. ^{13}C NMR (125 MHz, DMSO-d_6) and DEPT spectra of compound **3**;

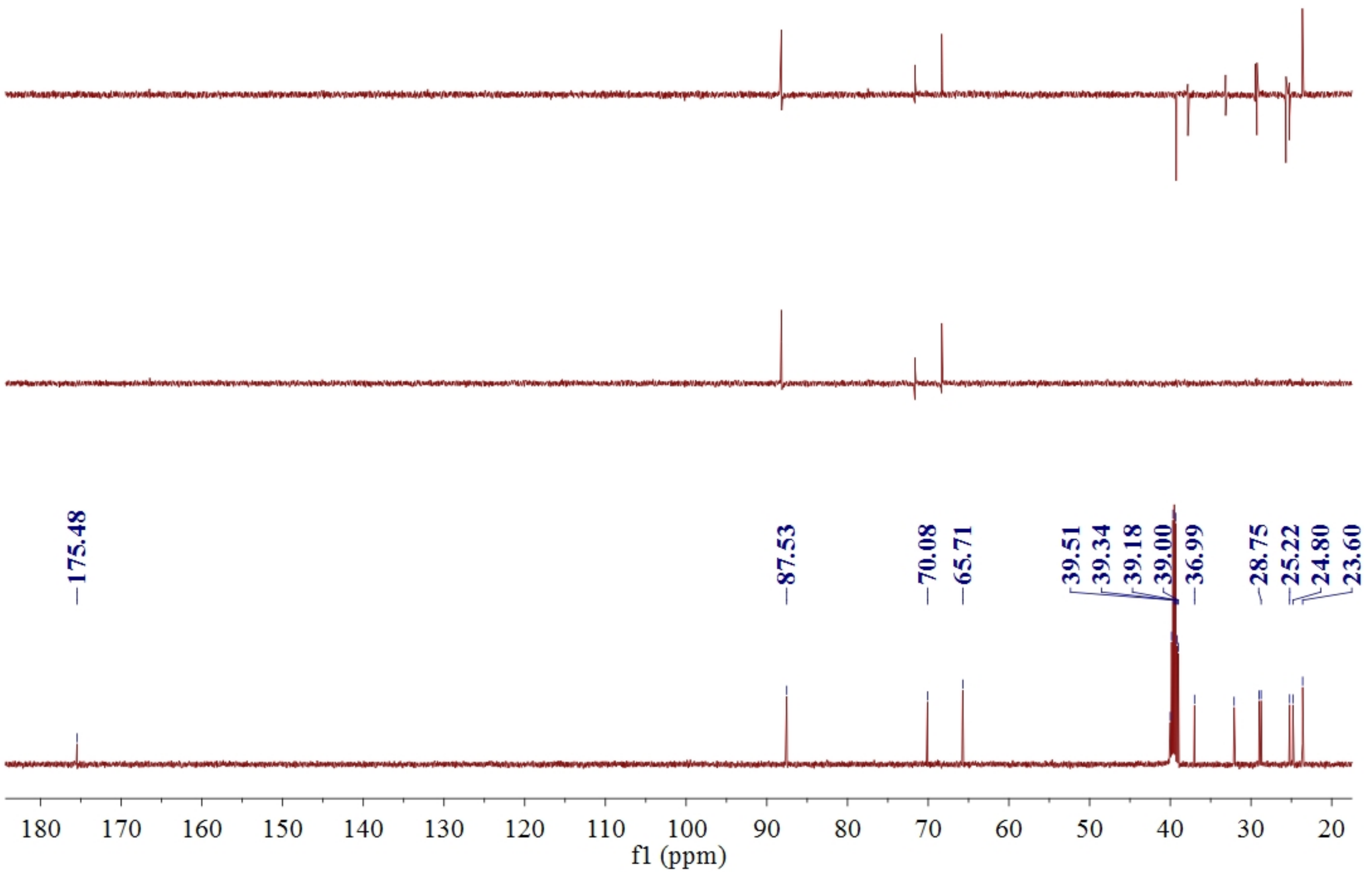

Figure S22. ^{13}C NMR (125 MHz, $CDCl_3$) and DEPT spectra of compound **3**;

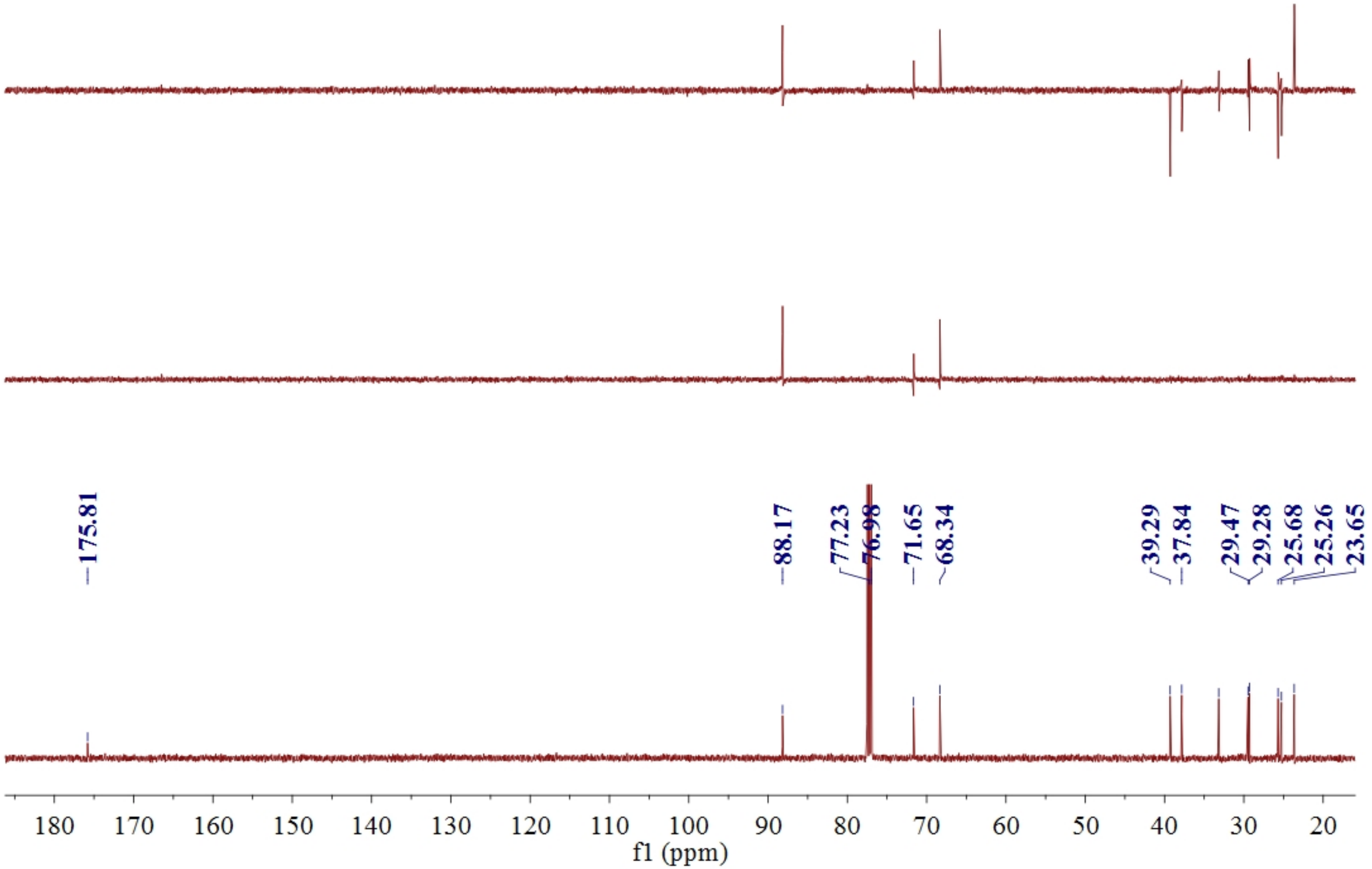

Figure S23. COSY spectrum of compound **3** ($CDCl_3$);

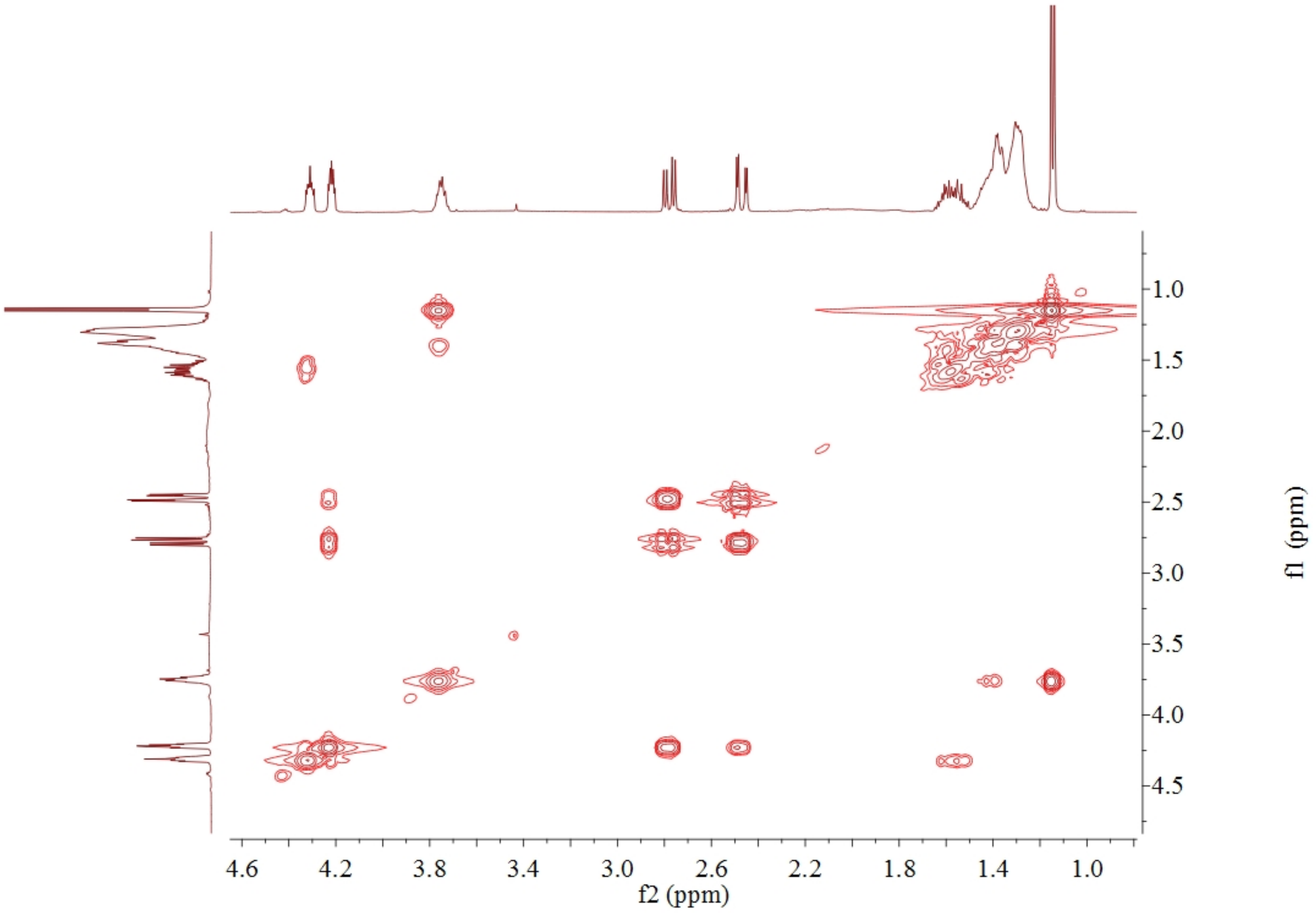

Figure S24. HSQC spectrum of compound **3** (DMSO-d_6);

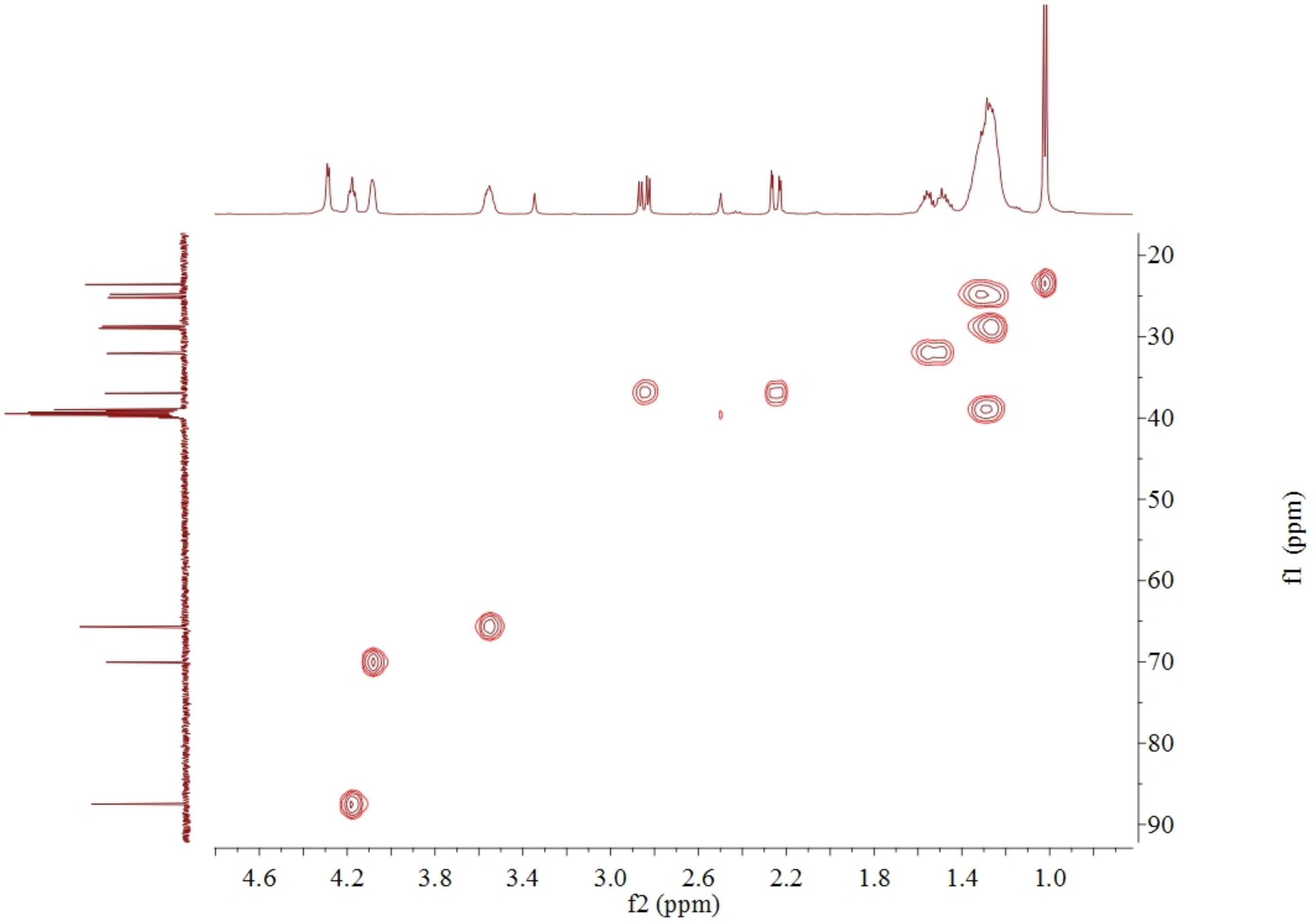

Figure S25. HMBC spectrum of compound **3** ($CDCl_3$);

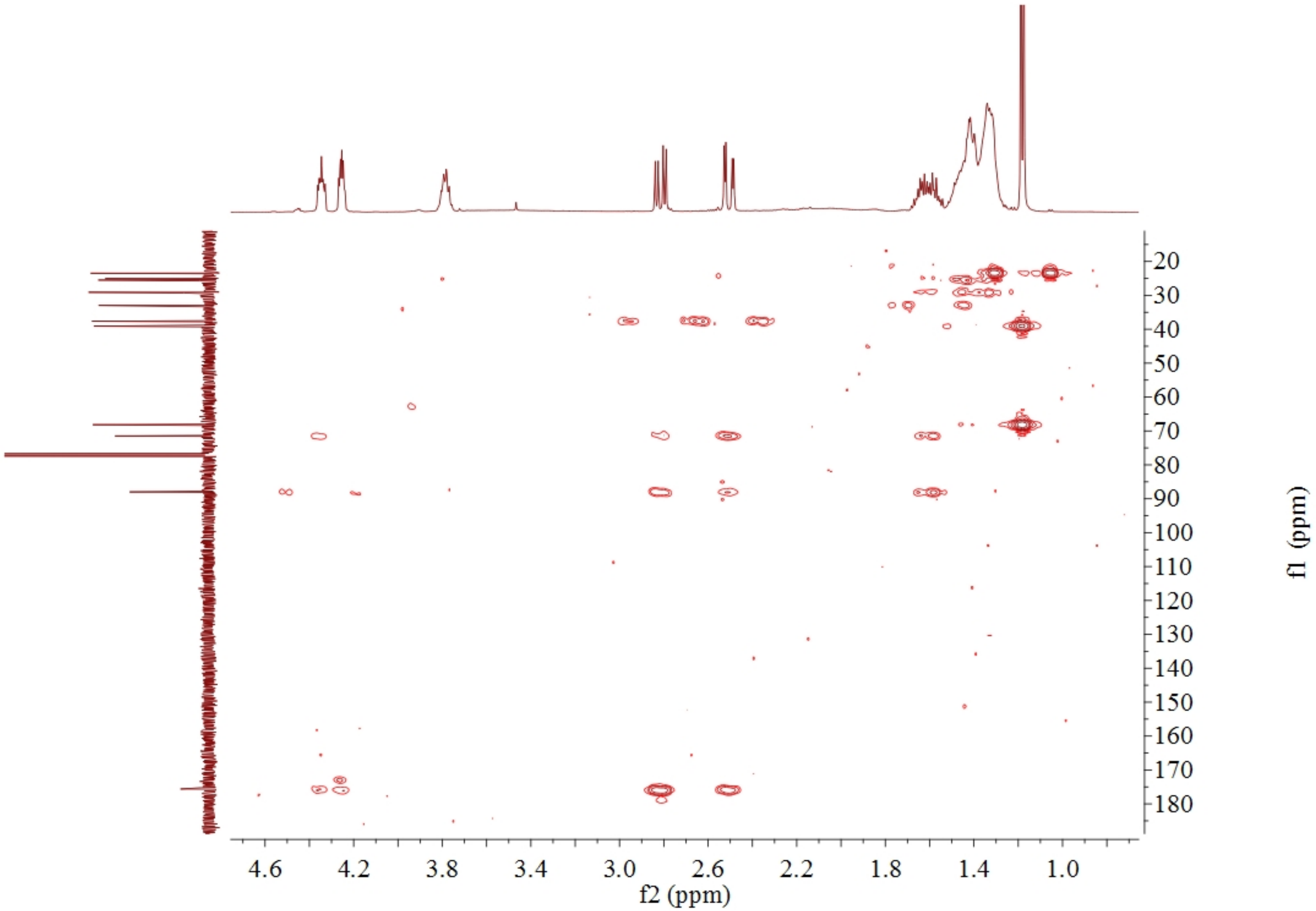

Figure S26. ECD spectrum of compound **3**;

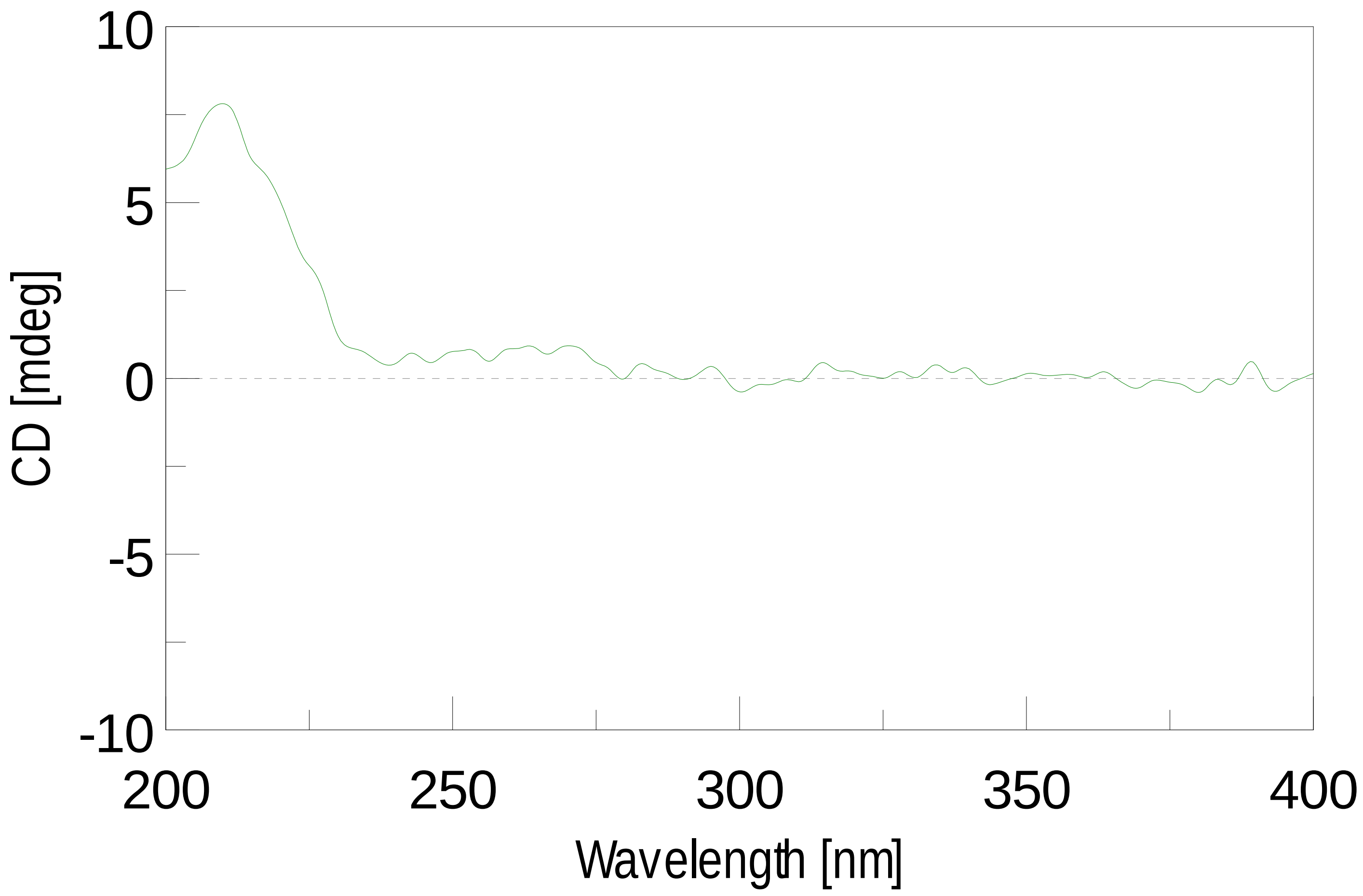

Figure S27. HRESI mass spectrum of compound **4**;

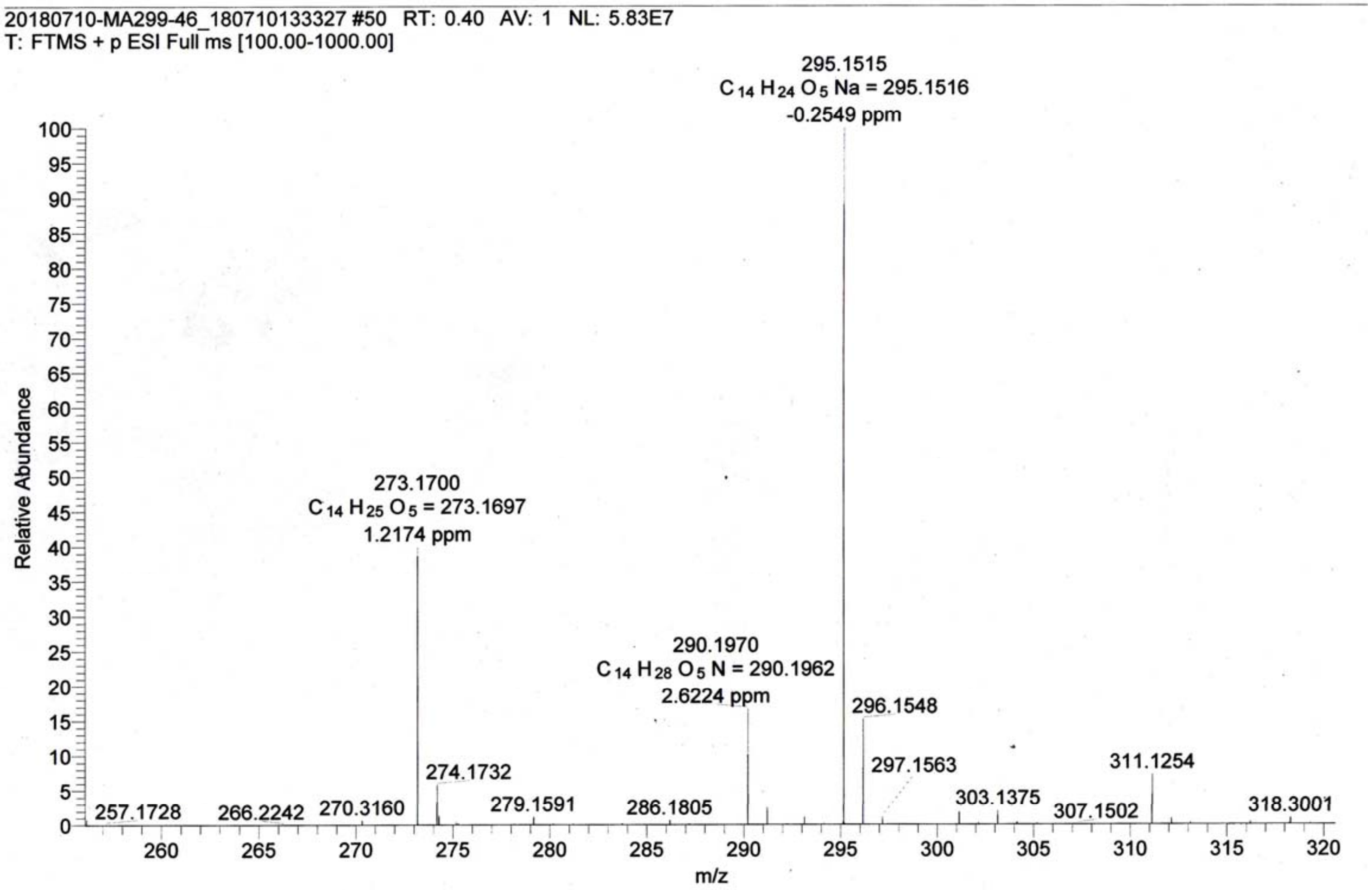

Figure S28. ^{1}H NMR (500 MHz, DMSO-d_6) spectrum of compound **4**;

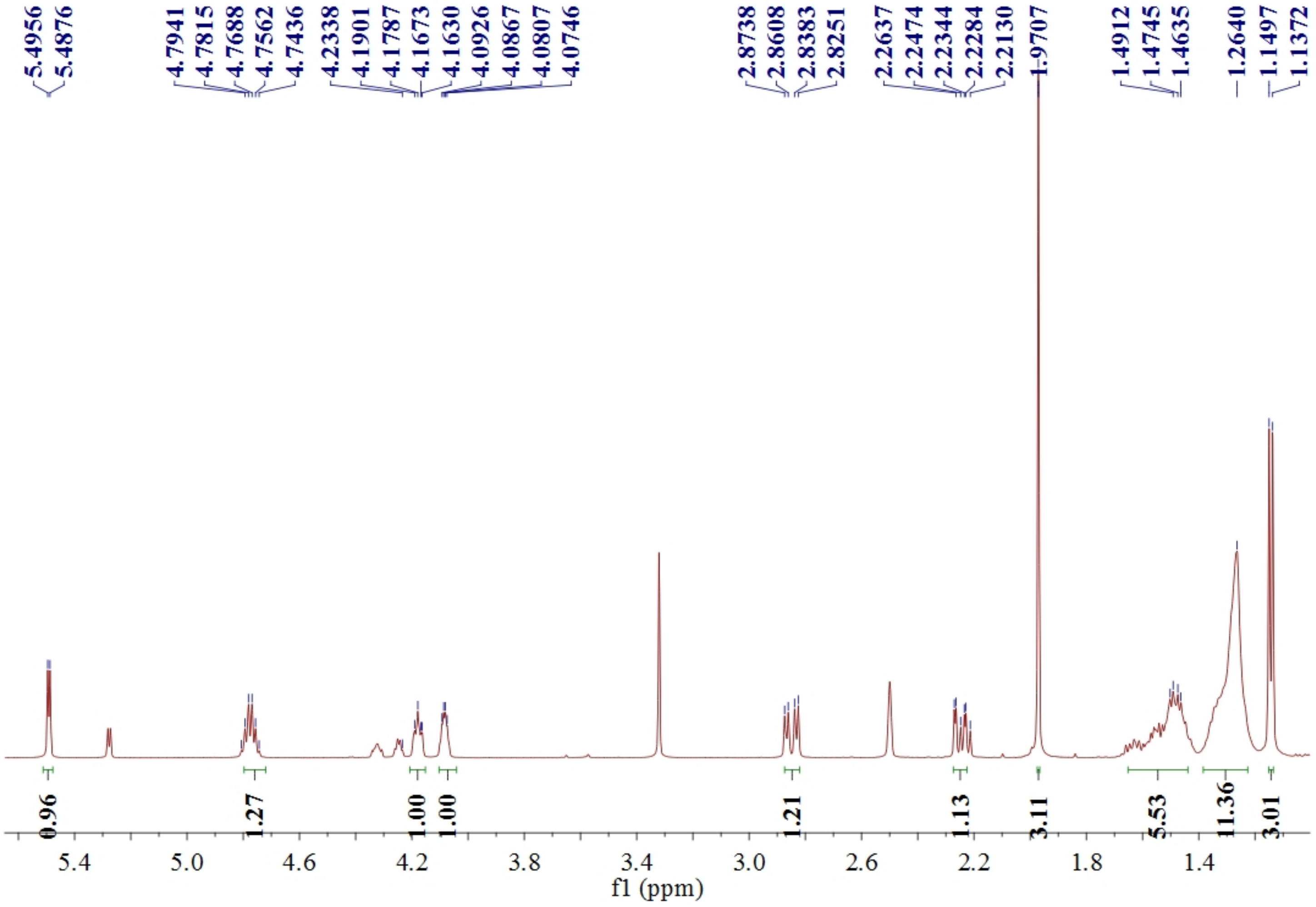

Figure S29. ^{13}C NMR (125 MHz, DMSO-d_6) and DEPT spectra of compound **4**;

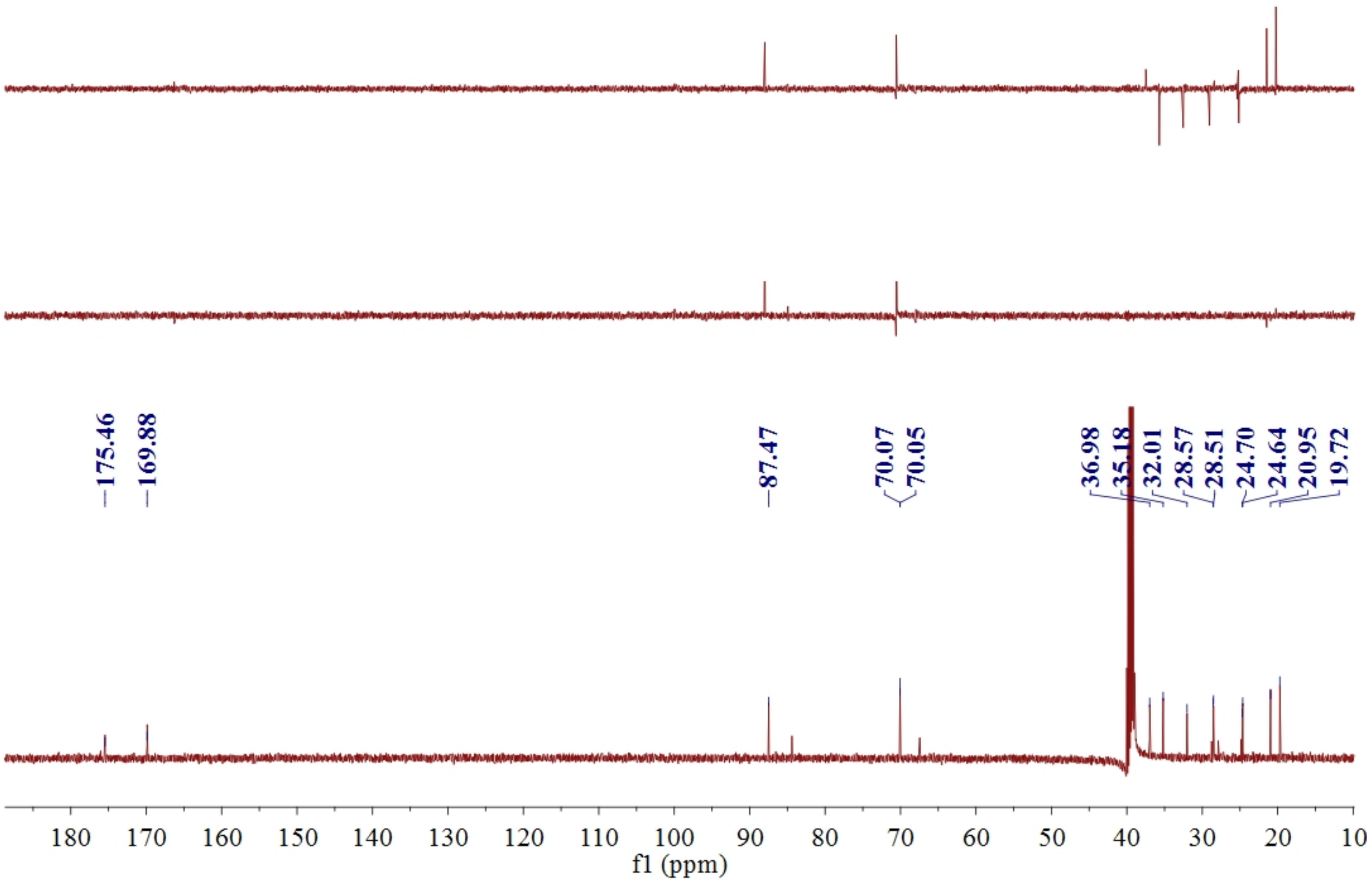

Figure S30. COSY spectrum of compound **4**;

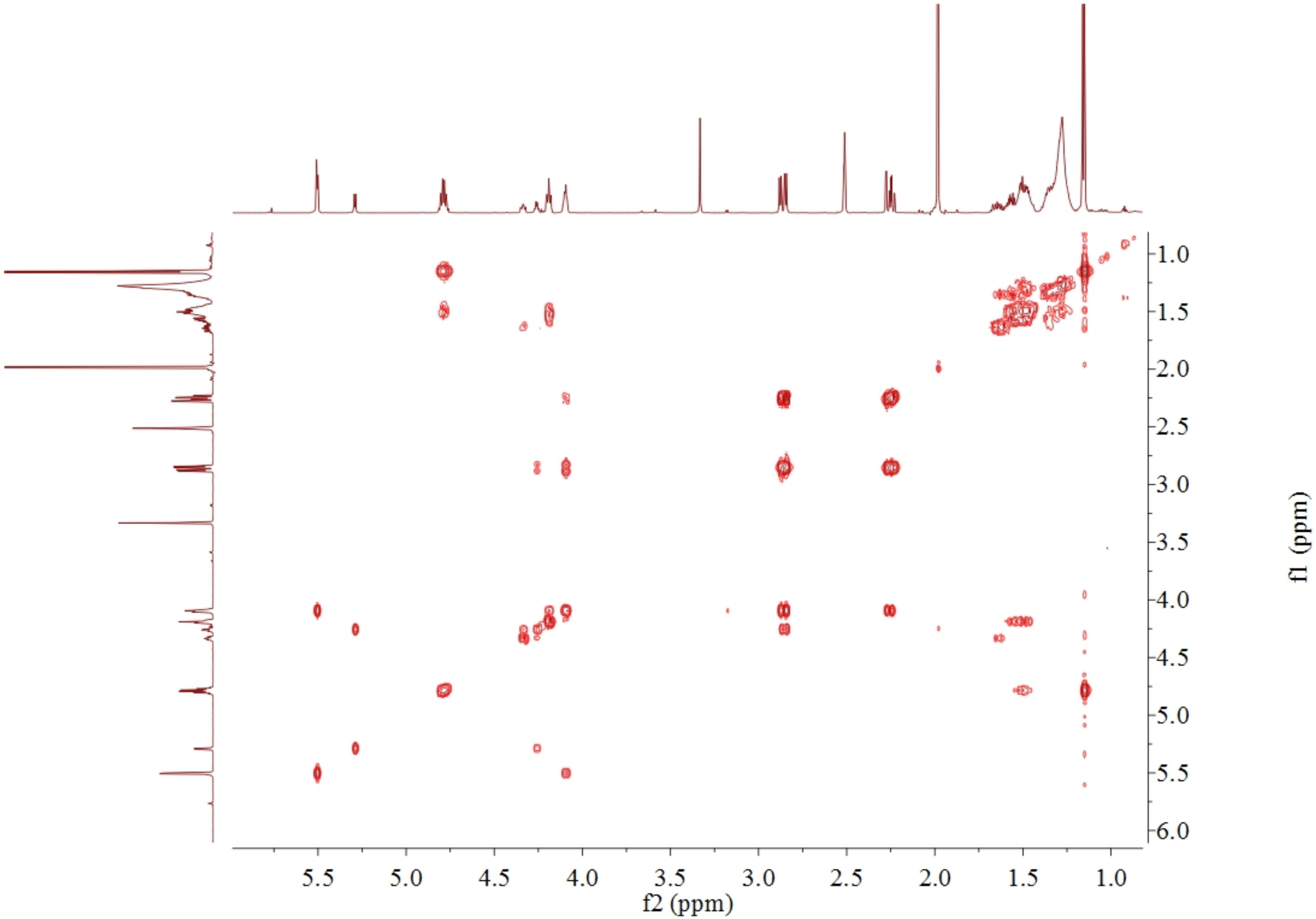

Figure S31. HSQC spectrum of compound **4**;

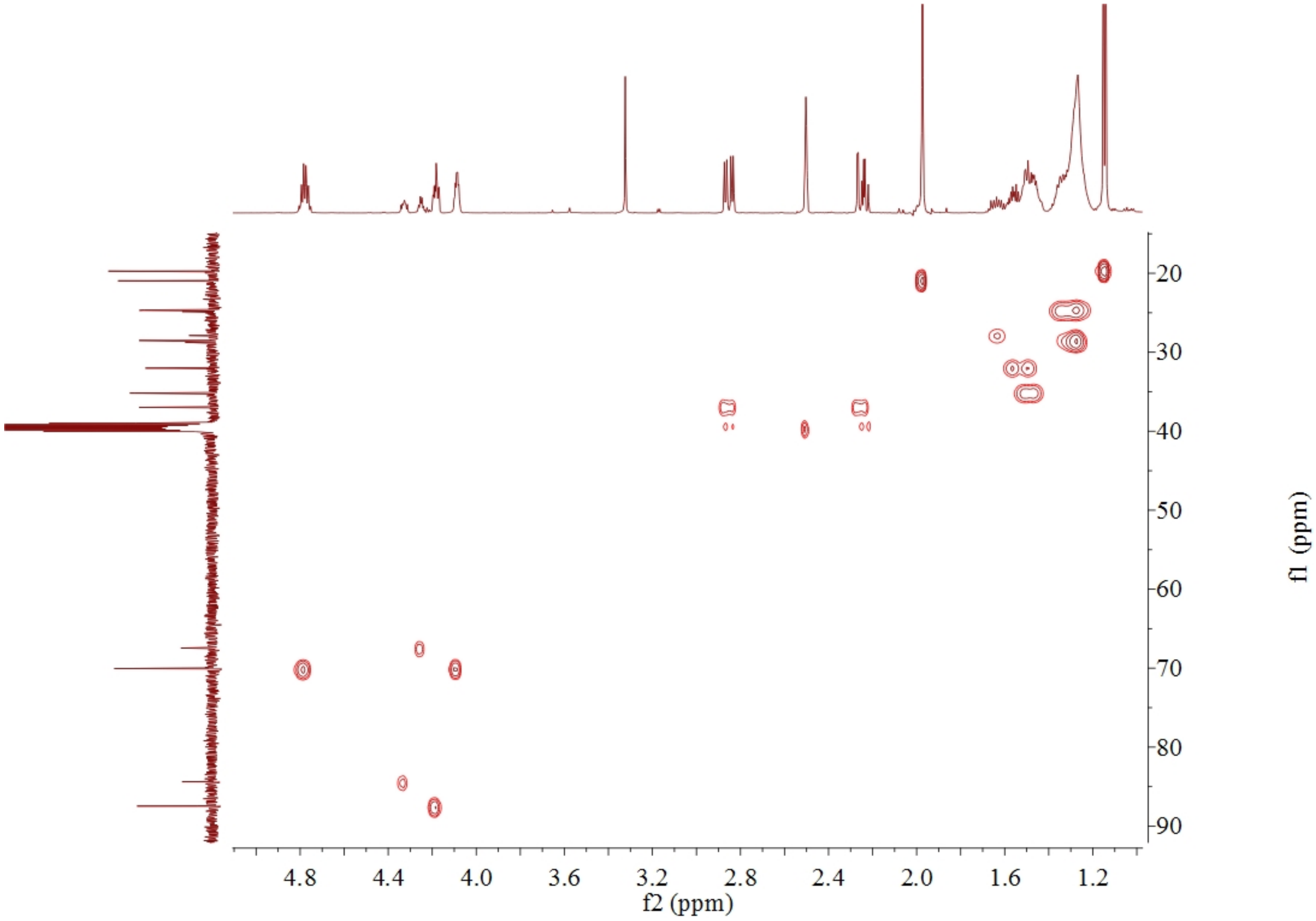

Figure S32. HMBC spectrum of compound **4**;

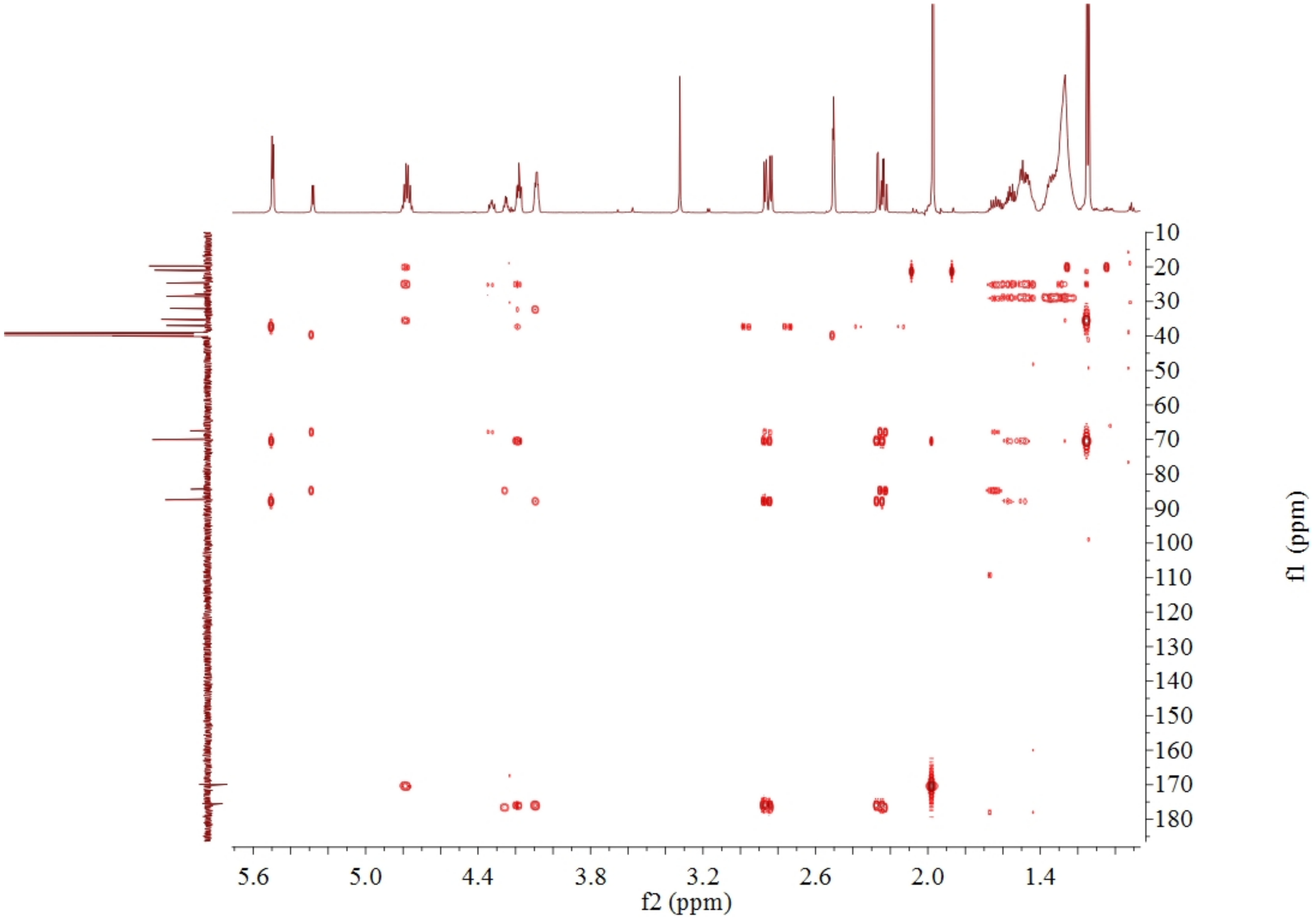

Figure S33. ECD spectrum of compound **4**;

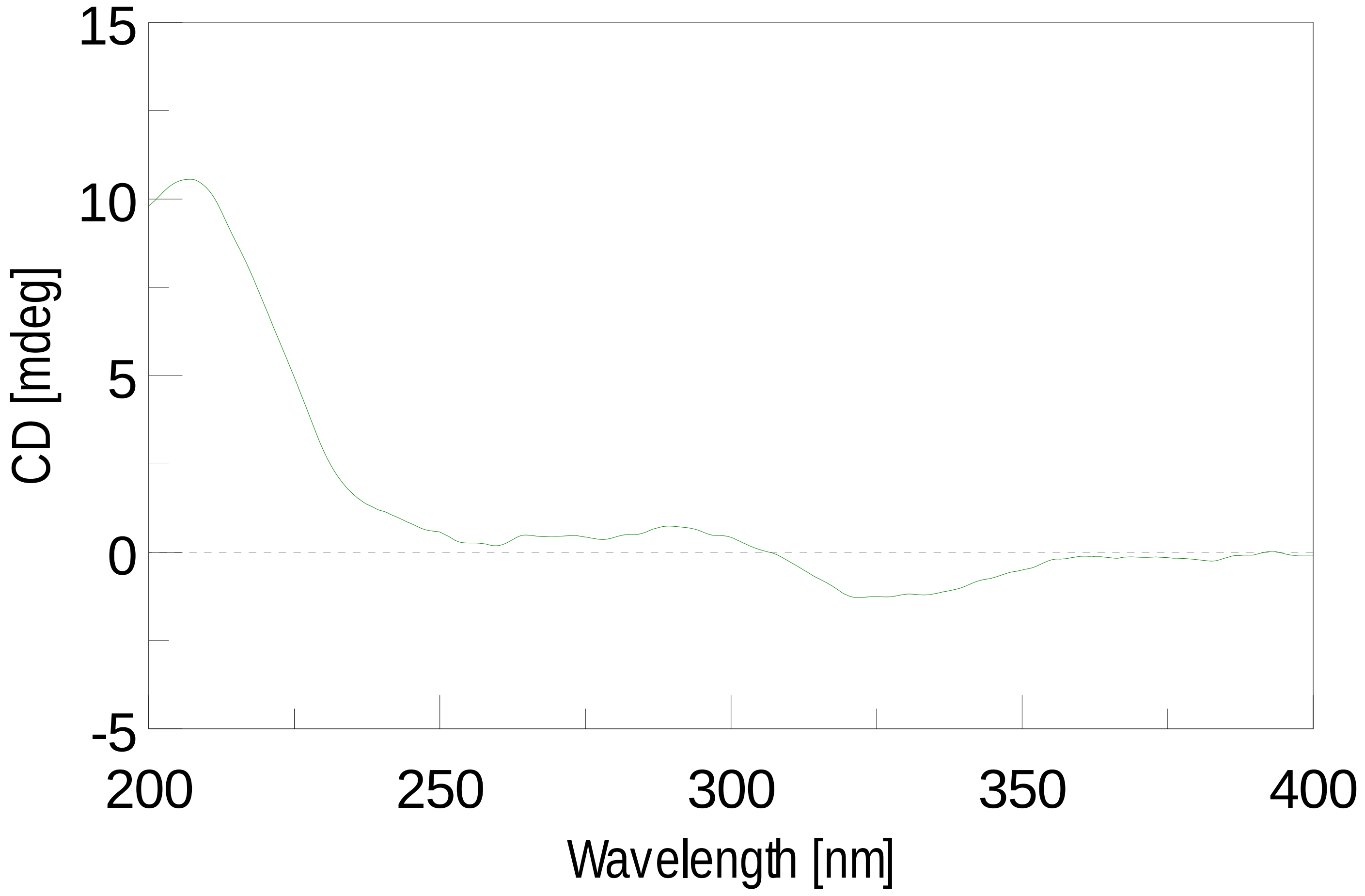

Figure S34. HRESI mass spectrum of compound **5**;

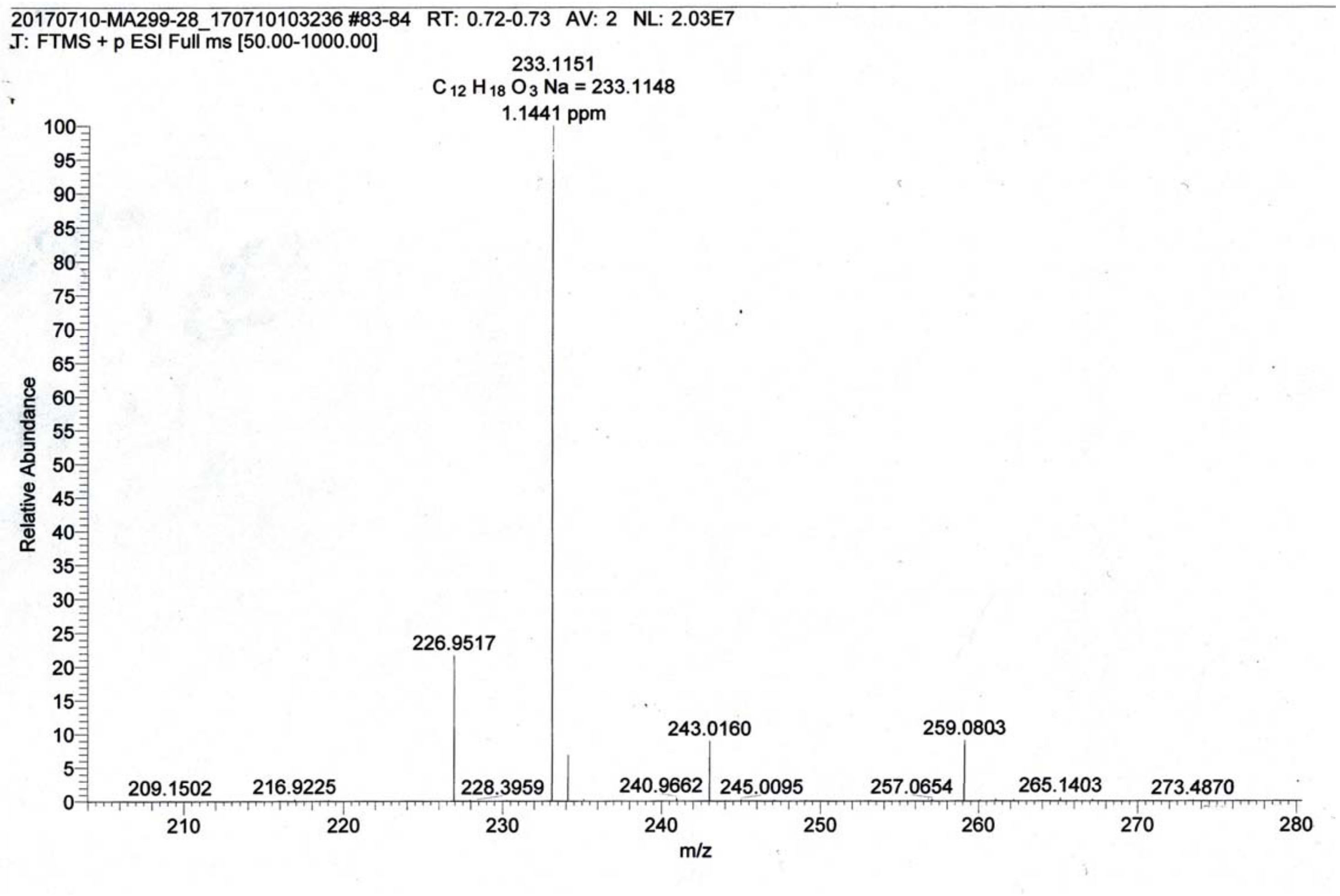

Figure S35. ^{1}H NMR (500 MHz, DMSO-d_6) spectrum of compound **5**;

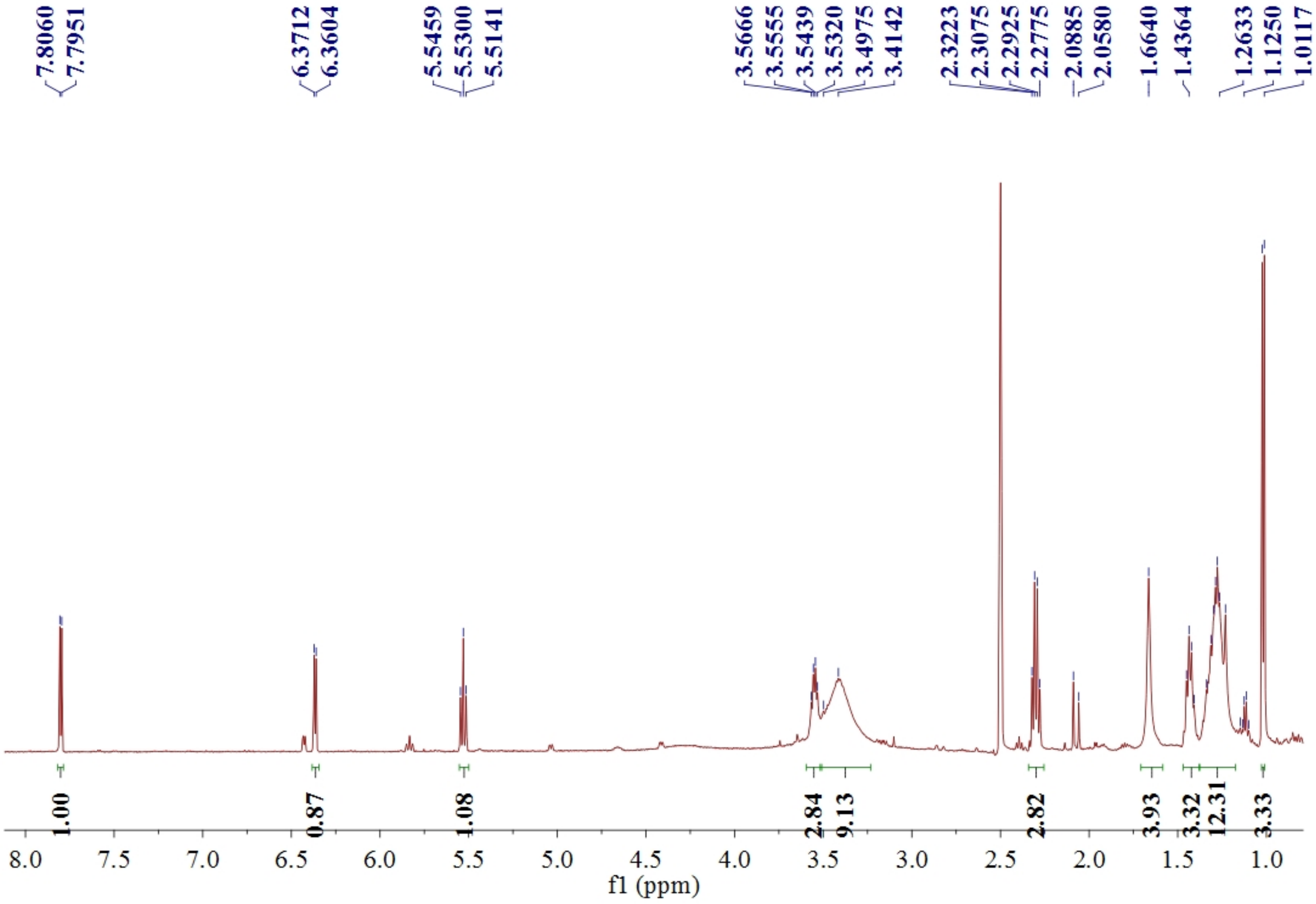

Figure S36. ^{13}C NMR (125 MHz, DMSO-d_6) and DEPT spectra of compound **5**;

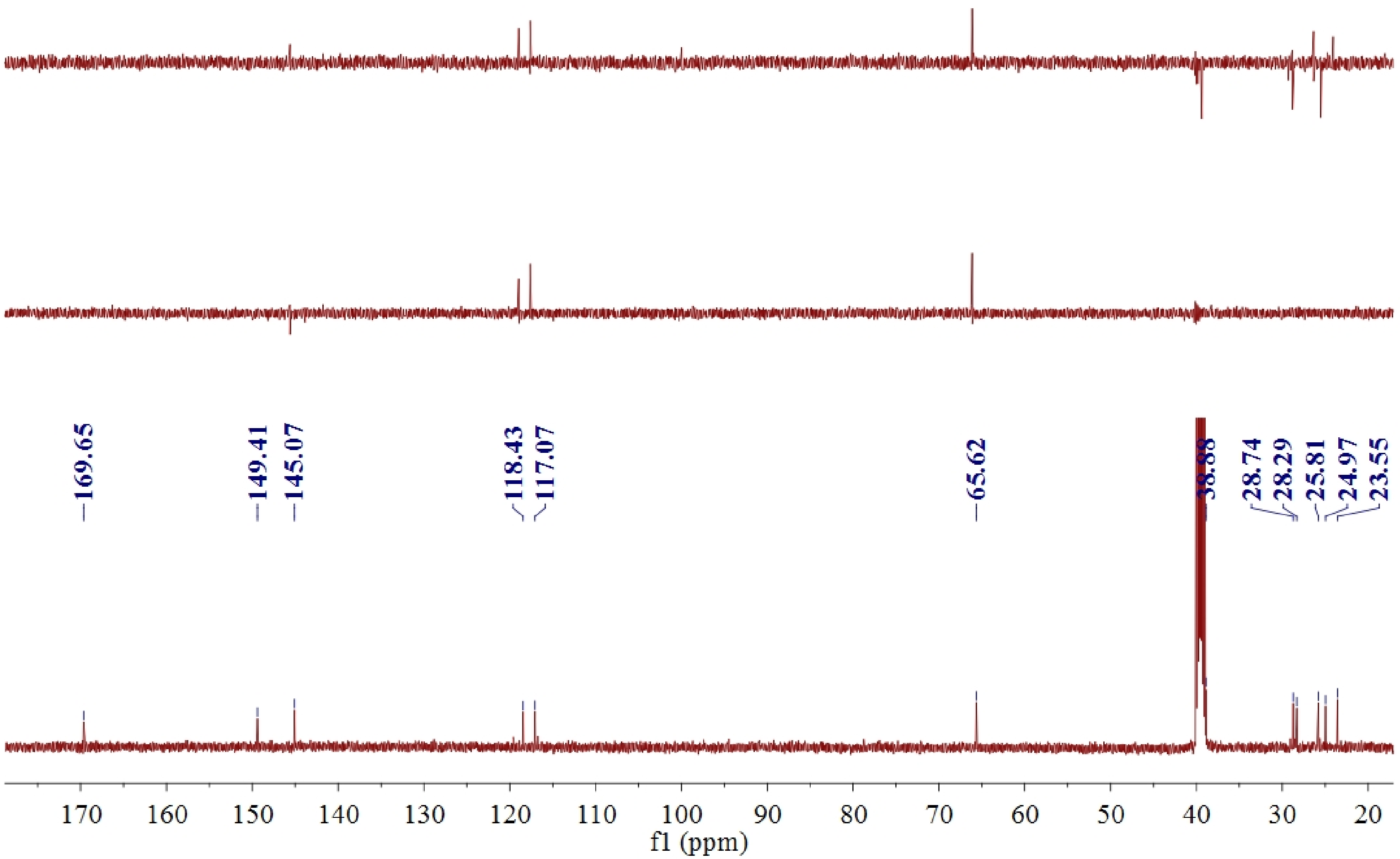

Figure S37. COSY spectrum of compound **5**;

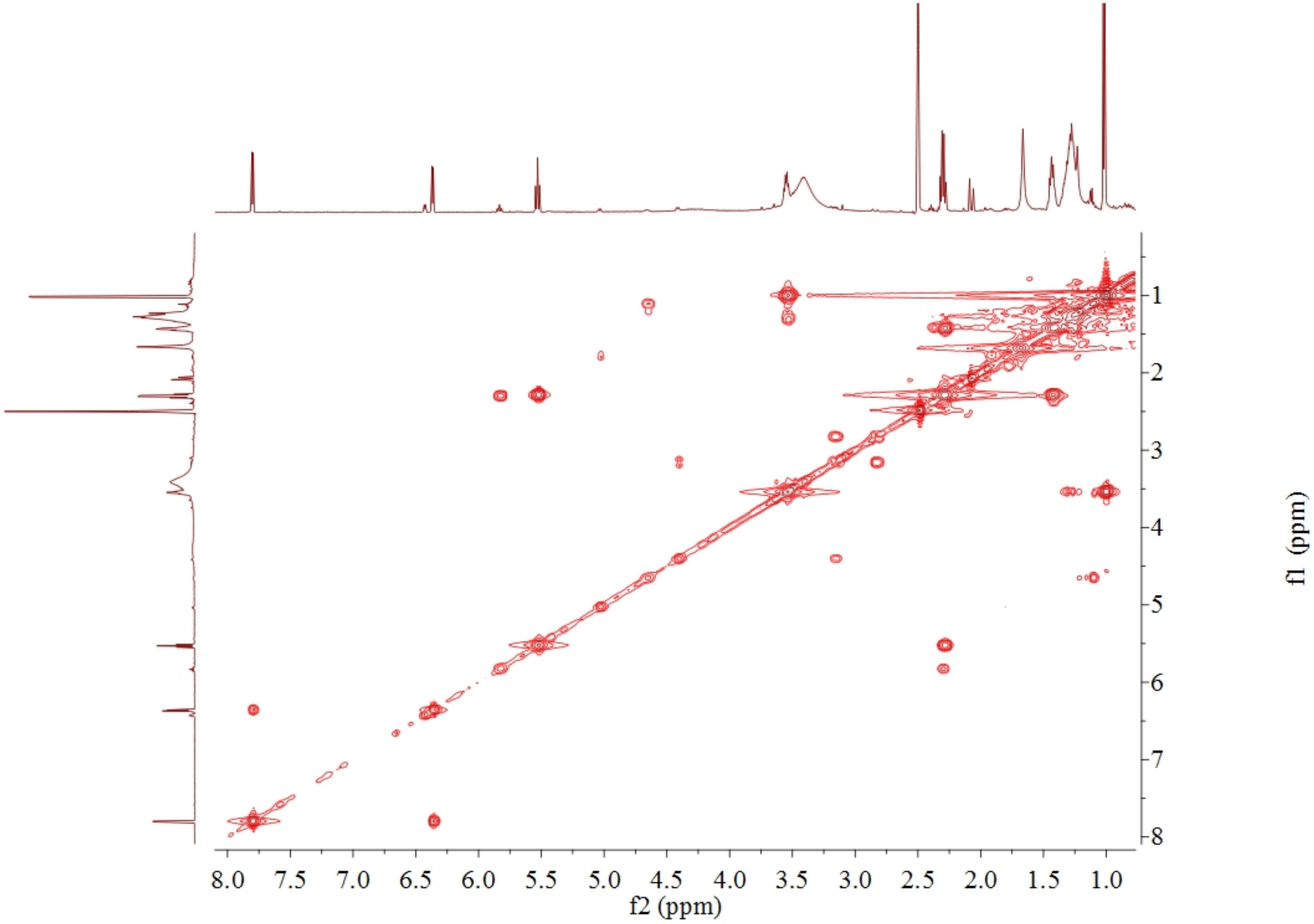

Figure S38. HSQC spectrum of compound **5**;

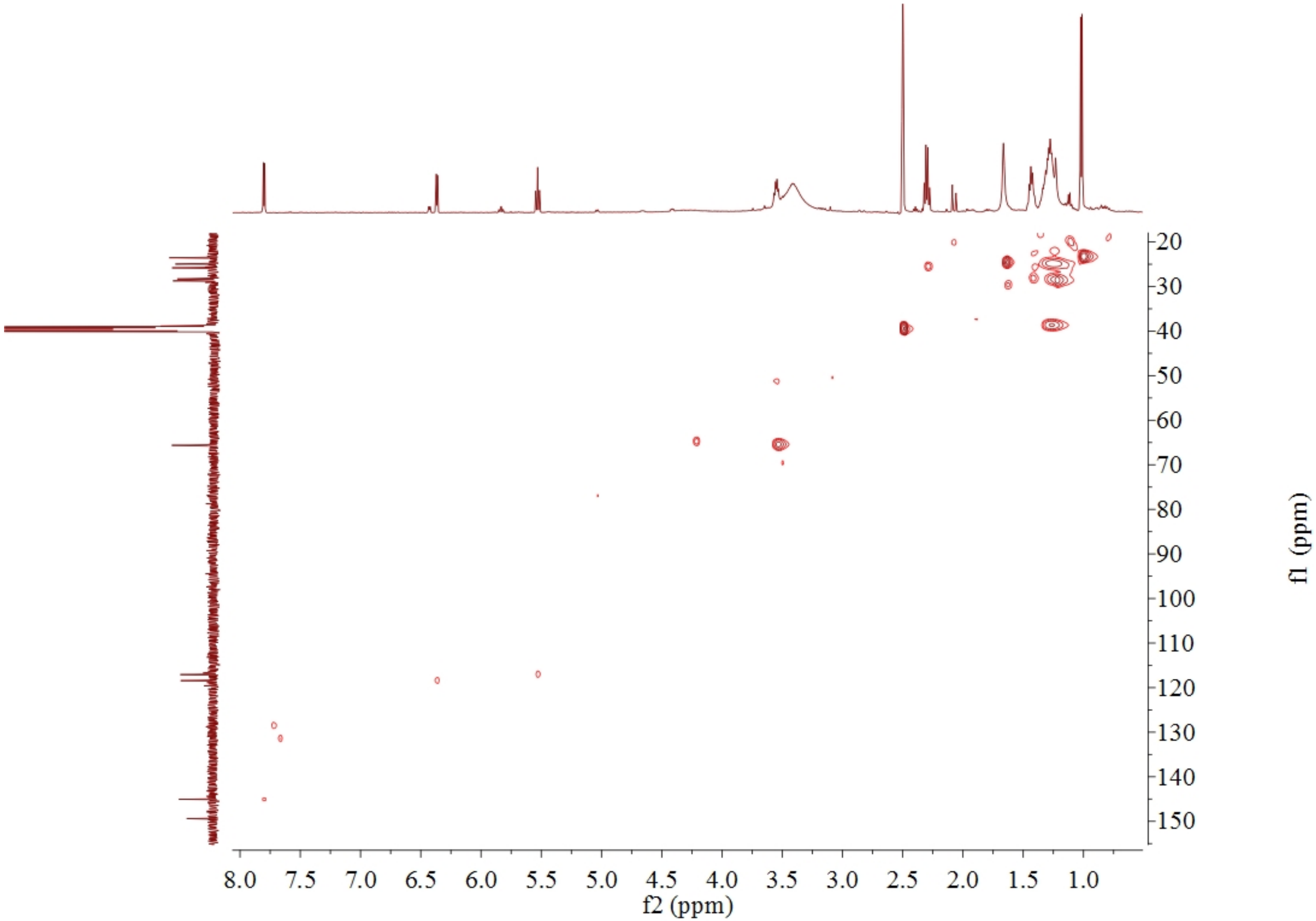

Figure S39. HMBC spectrum of compound **5**;

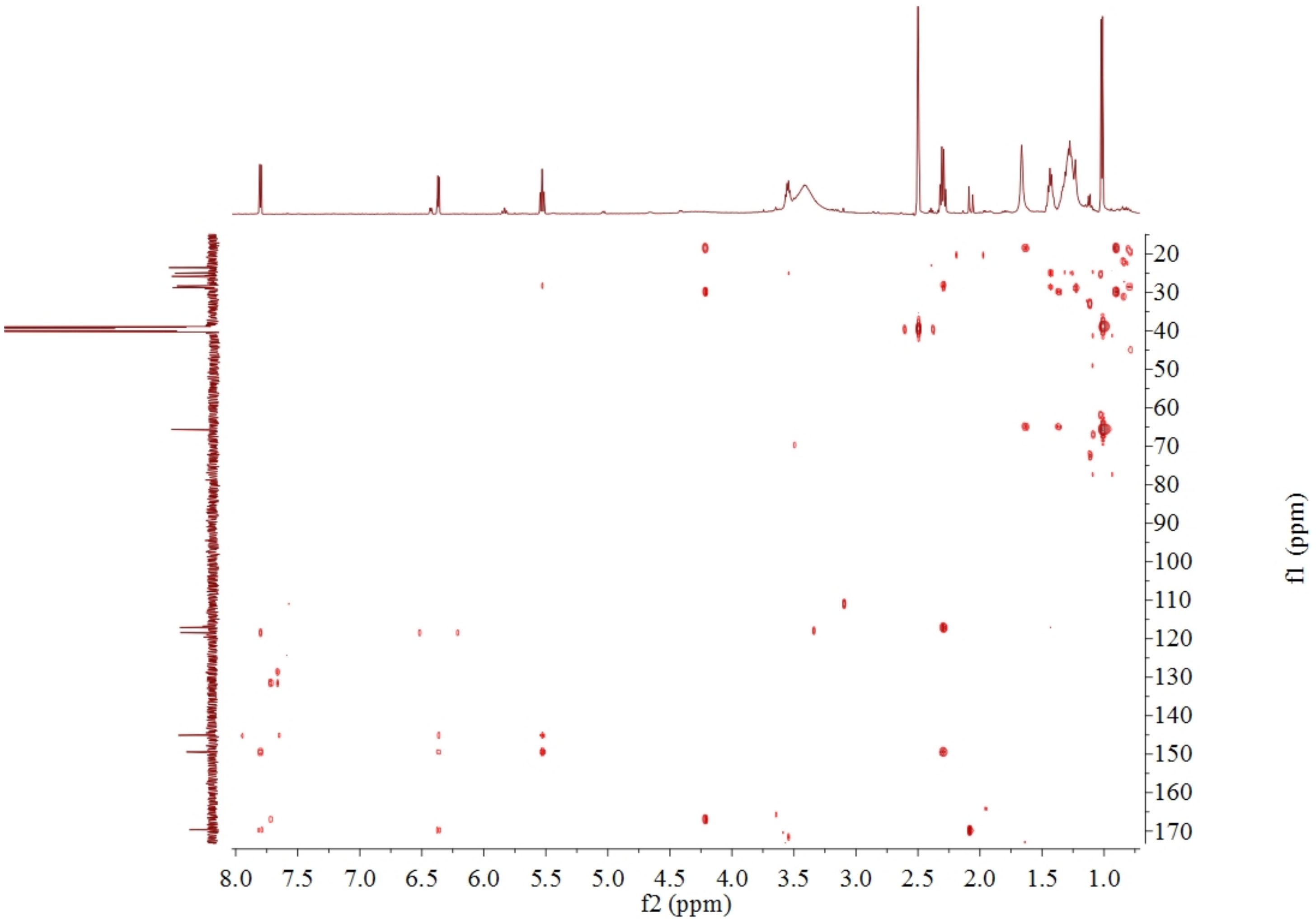

Figure S40. NOESY spectrum of compound **5**;

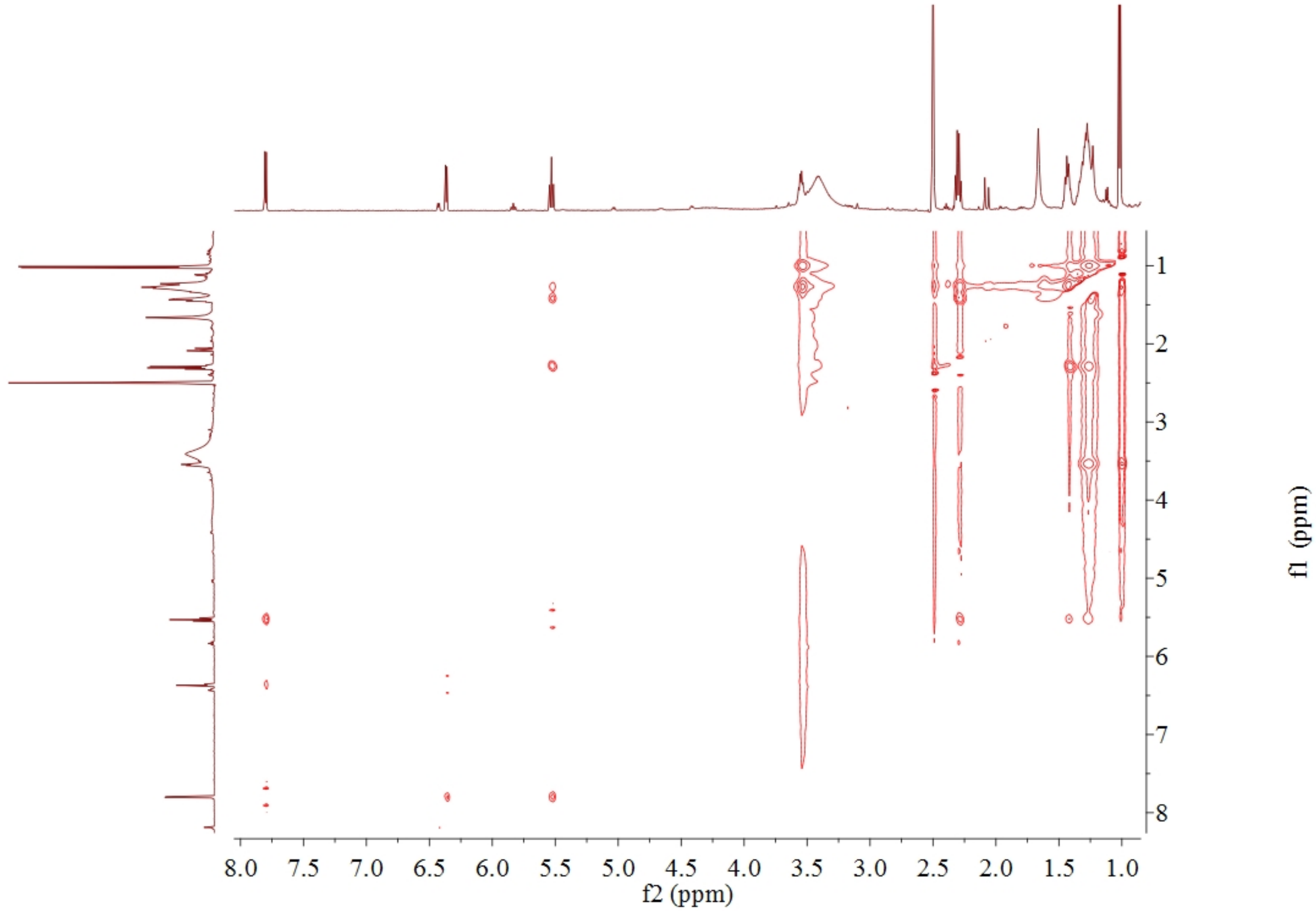

Figure S41. HRESI mass spectrum of compound **6**;

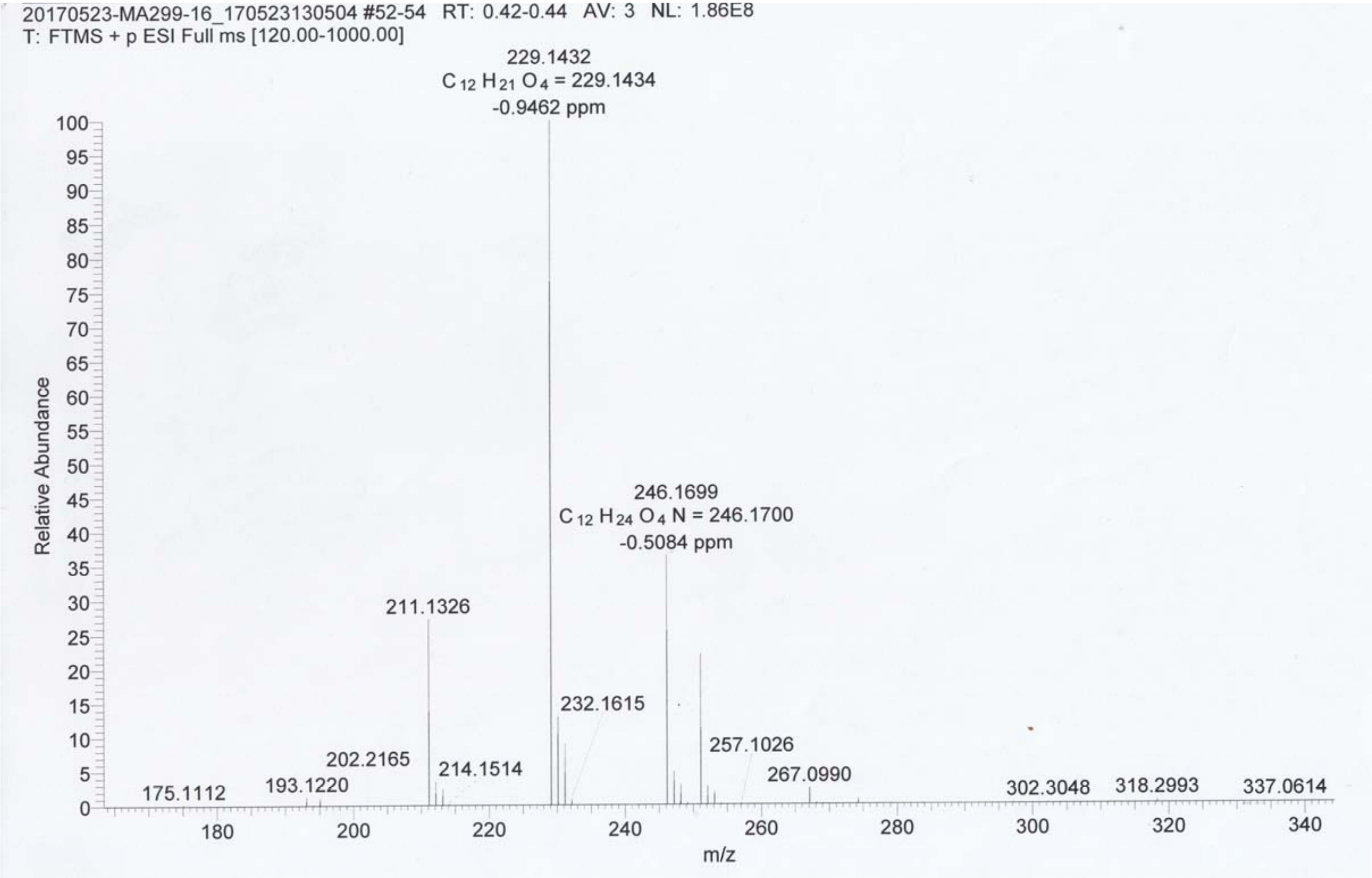

Figure S42. ^{1}H NMR (500 MHz, DMSO-d_6) spectrum of compound **6**;

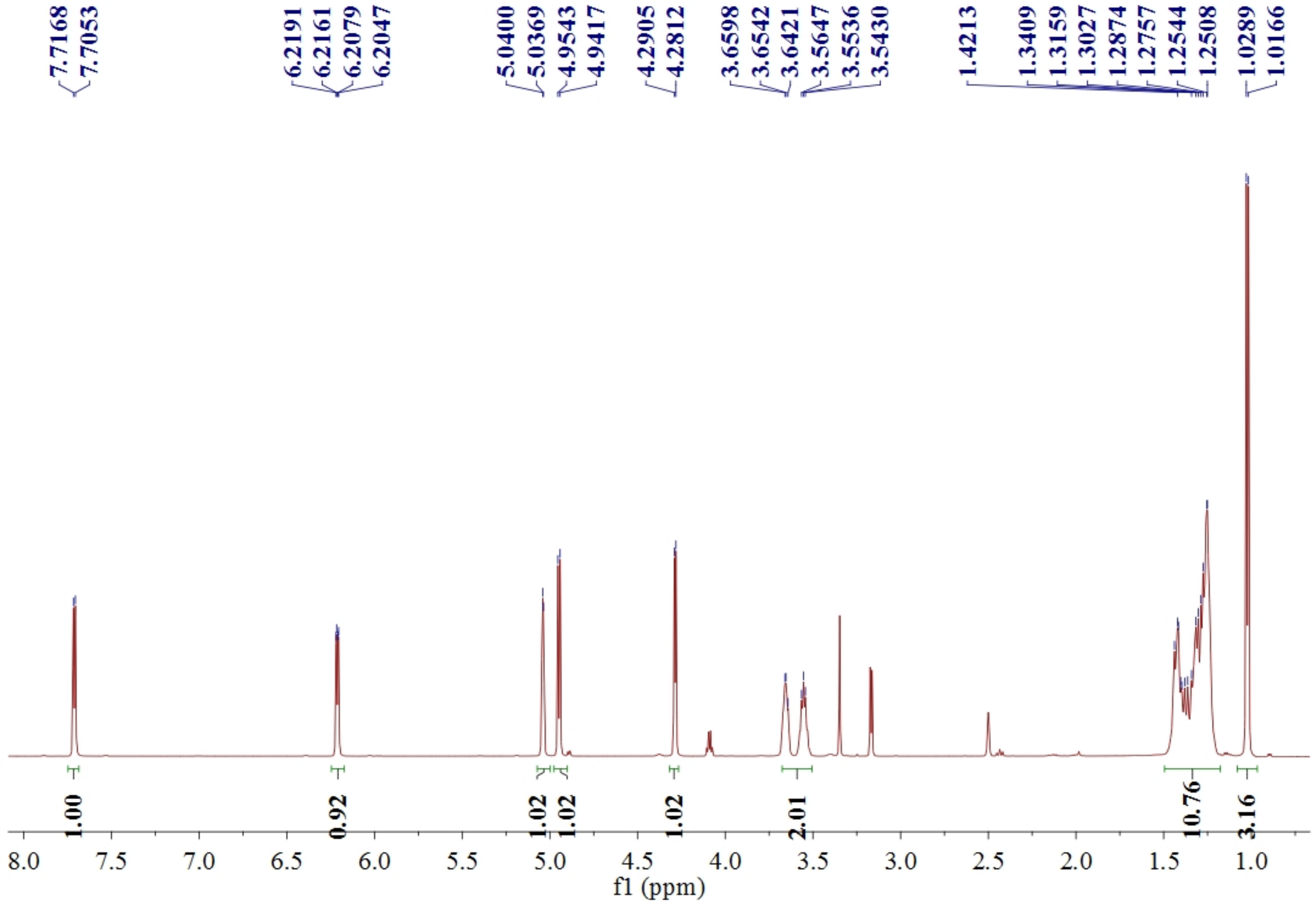

Figure S43. ^{13}C NMR (125 MHz, DMSO-d_6) and DEPT spectra of compound **6**;

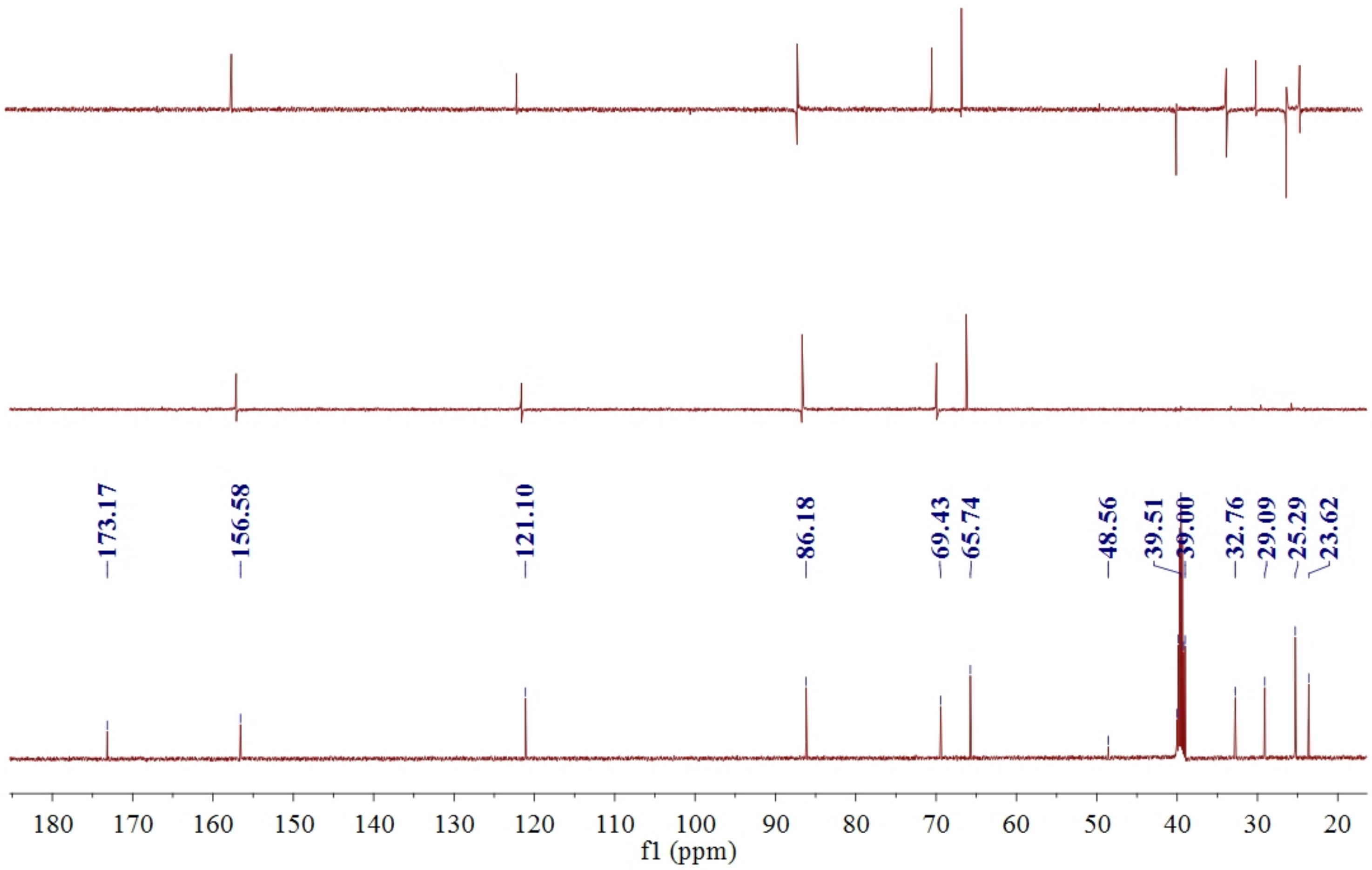

Figure S44. COSY spectrum of compound **6**;

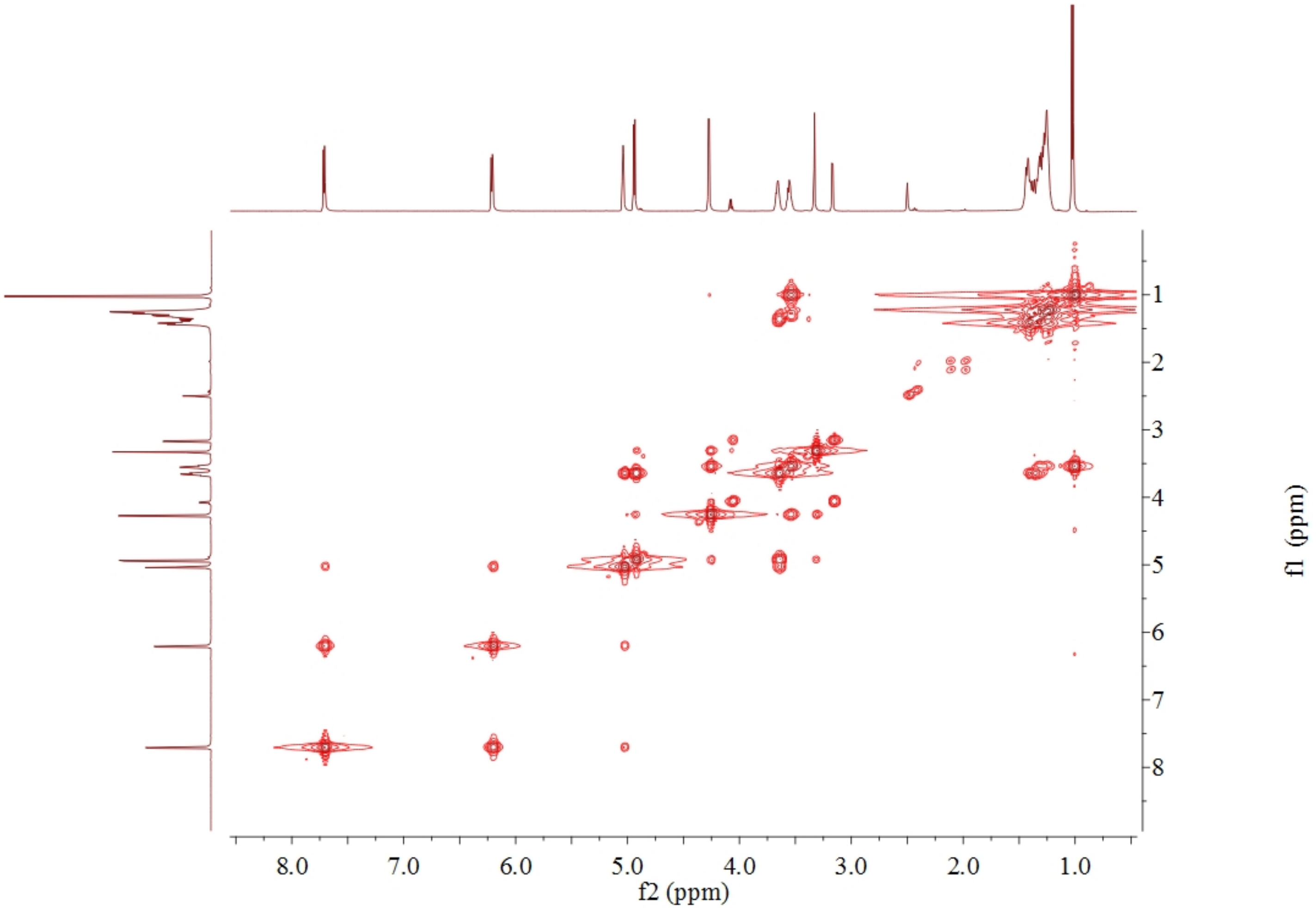

Figure S45. HMBC spectrum of compound **6**;

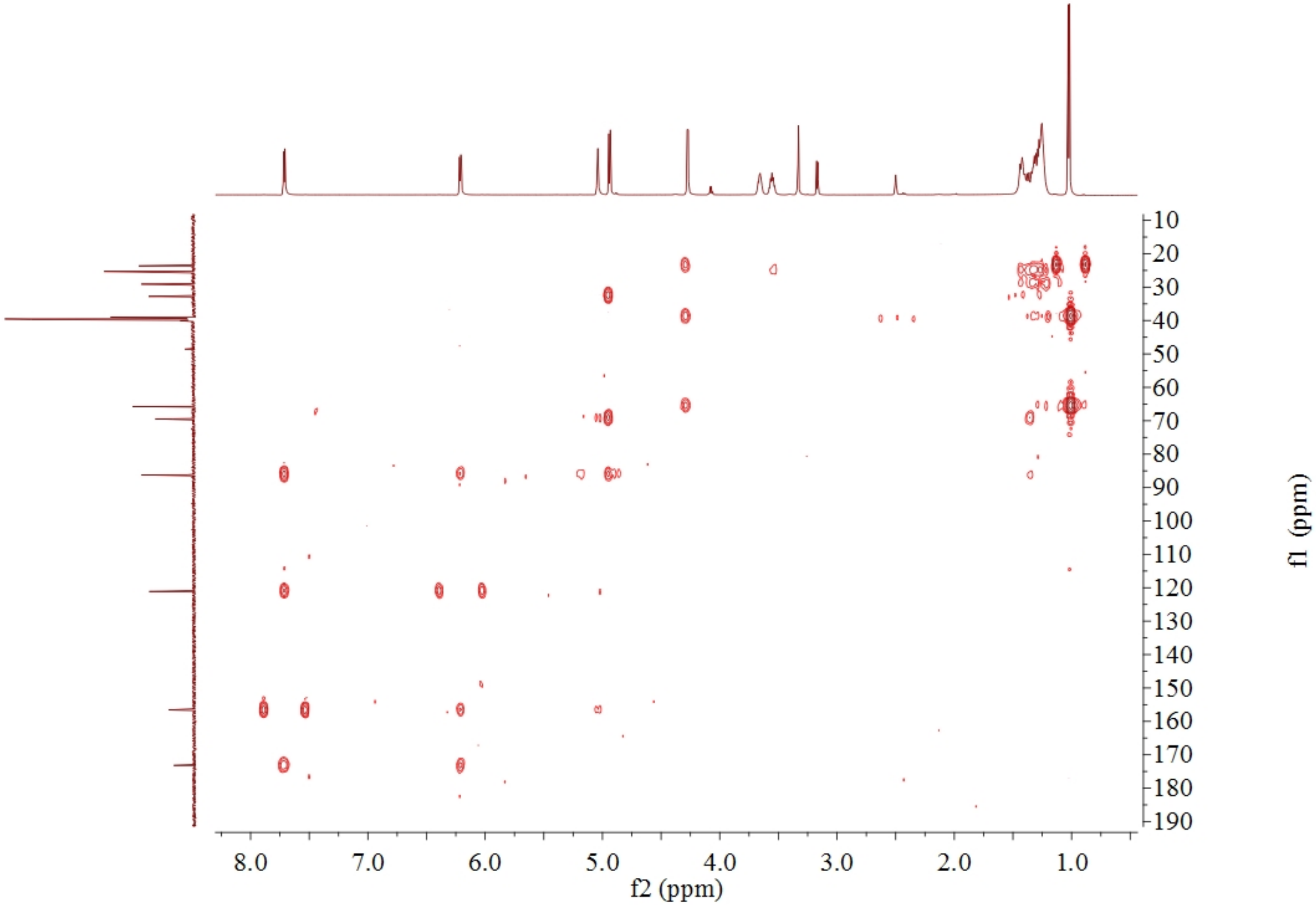

Figure S46. Crystal packing of compound **3**;

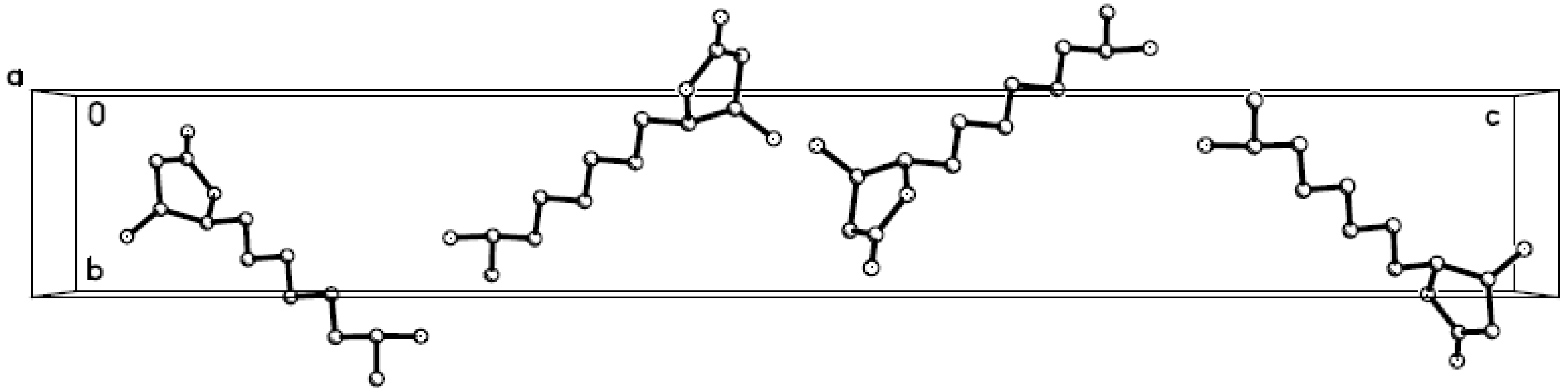

Figure S47. Crystal packing of compound **6**.

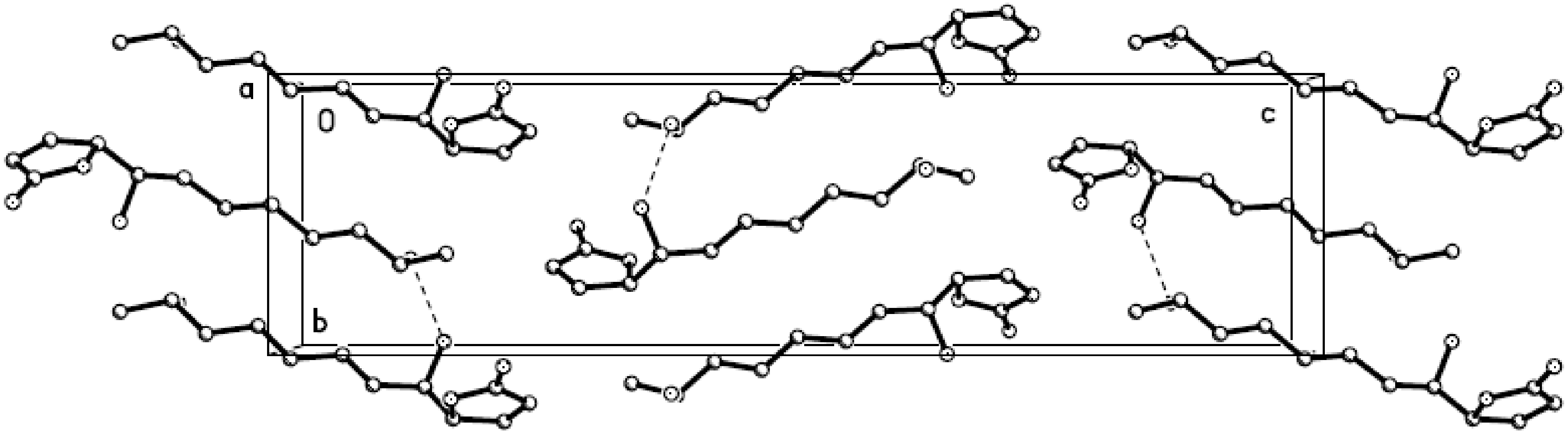

Supplement: Supplementary file 1 [file marinedrugs-17-00296-s001.pdf]
